# RPGeNet v2.0: expanding the universe of retinal disease gene interactions network

**DOI:** 10.1093/database/baz120

**Published:** 2019-11-11

**Authors:** Rodrigo Arenas-Galnares, Sergio Castillo-Lara, Vasileios Toulis, Daniel Boloc, Roser Gonzàlez-Duarte, Gemma Marfany, Josep F Abril

**Affiliations:** 1 Department of Genetics, Microbiology and Statistics, University of Barcelona, Barcelona, 08028, Catalonia, Spain; 2 Institute of Biomedicine (IBUB), University of Barcelona, Barcelona, 08028, Catalonia, Spain; 3 CIBERER, ISCIII, University of Barcelona, Barcelona, 08028, Catalonia, Spain; 4 Faculty of Medicine, University of Barcelona, Barcelona, 08036, Catalonia, Spain; 5 DBGen Ocular Genomics, Barcelona, 08028, Catalonia, Spain

## Abstract

RPGeNet offers researchers a user-friendly queriable tool to visualize the interactome network of visual disorder genes, thus enabling the identification of new potential causative genes and the assignment of novel candidates to specific retinal or cellular pathways. This can be highly relevant for clinical applications as retinal dystrophies affect 1:3000 people worldwide, and the causative genes are still unknown for 30% of the patients. RPGeNet is a refined interaction network interface that limits its skeleton network to the shortest paths between each and every known causative gene of inherited syndromic and non-syndromic retinal dystrophies. RPGeNet integrates interaction information from STRING, BioGRID and PPaxe, along with retina-specific expression data and associated genetic variants, over a Cytoscape.js web interface. For the new version, RPGeNet v2.0, the database engine was migrated to Neo4j graph database manager, which speeds up the initial queries and can handle whole interactome data for new ways to query the network. Further, user facilities have been introduced as the capability of saving and restoring a researcher customized network layout or as novel features to facilitate navigation and data projection on the network explorer interface. Responsiveness has been further improved by transferring some functionality to the client side.

## Introduction

Inherited retinal dystrophies (IRDs) comprise a highly heterogeneous group of disorders caused by over 200 causative genes (1). The prevalence of IRDs is 1:3000 worldwide, which make these blinding disorders a health relevant target. The implementation of massive sequencing approaches has greatly facilitated genetic testing and, as a result, the number of IRD genes and mutations is constantly increasing. Nonetheless, a substantial number of cases remain to be accurately diagnosed, as the average yield in IRD genetic diagnosis is roughly 50%.

Besides technical limitations, one of the bottlenecks in massive sequence-based molecular diagnosis is that most identified variants are previously unreported missense changes of unknown pathogenicity, either in known causative genes or in previously unreported candidates. These variants may be deemed as pathogenic or probably damaging by *in silico* predictive programs but end up classified as genetic variants of unknown significance, since there are not functional analyses to support their pathogenicity and their relationship to retinal physiology is yet to be determined.

Molecular medicine based on gene and protein networks is rapidly expanding since most disease-causing genes often work together, either forming a protein complex or participating in the same signalling pathways. In contrast to the analysis of isolated genes, finding the networks that link disease candidate genes provides supporting data for the identification of new causative genes, functional clues for assaying putative pathogenic novel variants, as well as opens new scenarios to identify key therapeutic targets.

Comprehensive tools to navigate through gene functions, cellular pathways and pathogenicity begin to emerge, particularly for cancer research. Although a considerable amount of genetic and functional data on IRDs genes and mutations has been gathered, there are not many user-friendly searchable tools to make a network map of gene/proteins interactions. To fill this gap, a web application, RPGeNet (2), was implemented that integrated all the physical and genetic interactions at that time for a subset of IRD genes (retinitis pigmentosa and Leber congenital amaurosis), obtained from different databases and including additional data such as tissue-specific expression. The expansion of genetic data, plus all the interactome and other omics information gathered of late, has prompted us to review and expand the initial 100 genes to more than 200 retinal dystrophy genes, to include distilled interaction databases and to implement an improved network generation, management, and visualization interface.

### Materials and Methods

**Figure 1:**
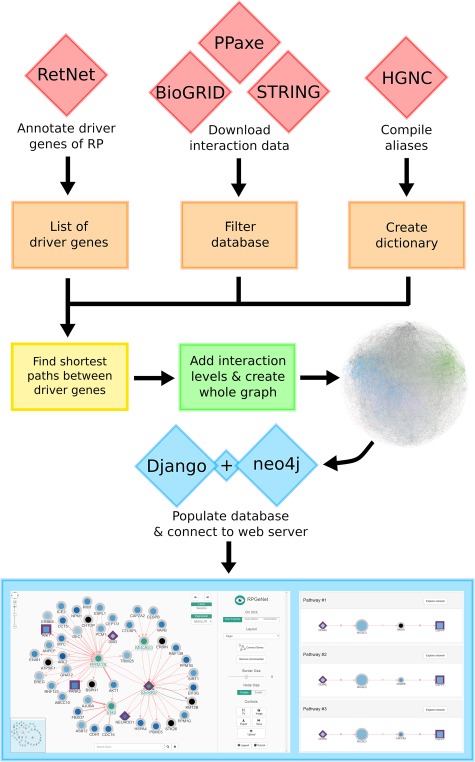
Data integration workflow to build the RPGeNet core database. The final graphical web interface (bottom panels) depends on a series of data integration steps that provide the interactions and nodes to the main neo4j database engine. Each component of the workflow is described in detail on the main text.

**Figure 2:**
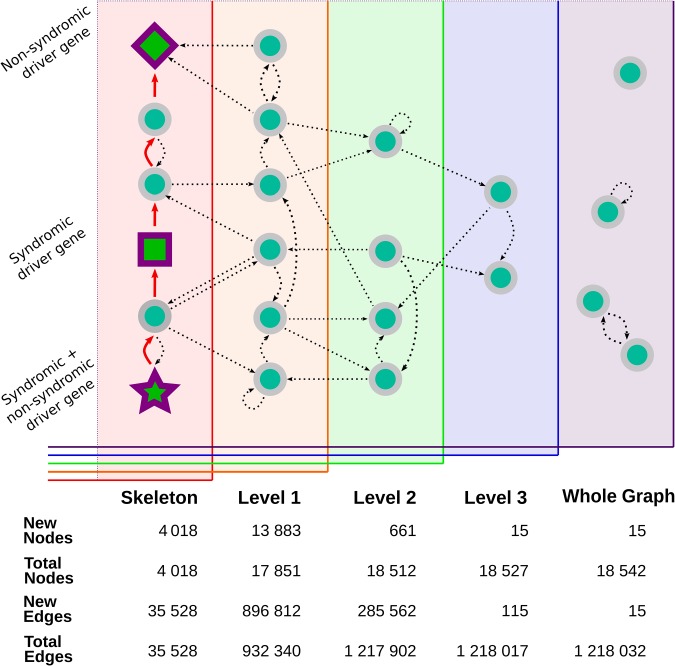
Visual representation of the expansion of the core interaction graph. The graph builder begins with the construction of the skeleton graph, represented by all the nodes on the leftmost panel. The skeleton is created by finding the shortest direct interaction path (the red arrows) between all the known driver genes—drawn here using the same shapes as in RPGeNet Network Explorer (star, square and diamond shapes, based on whether their mutations cause syndromic diseases or not). The graph is then expanded into level one (represented as the orange panel) by adding all of the parents and children (straight lines) of the nodes already found in the skeleton graph. Level-specific interactions are shown as curved connections linking nodes within a given graph level. The expansion is repeated until the highest level is reached and all known genes with known interactions have been connected to the interactions core graph. The remaining genes that do not have any known interaction that connects them with the core graph are included in the whole graph level. Some of those genes may have interactions with other genes found only in the whole graph level (and many of those interactions are self-references). The bottom table from this figure compares the number of nodes and edges added on each level (‘New Nodes’ and ‘New Edges’ rows), as well as the total number of nodes and edges accumulated.

RPGeNet is a tool to assist in the search for potential candidate genes and pathways associated with retinitis pigmentosa and intended to be used by both research and genetic diagnostic purposes. RPGeNet refines the vast interaction network by reducing it to the shortest paths between known driver genes of the disease (from now on the so-called skeleton network). The reduced network allows users to better identify genes interconnecting drivers that can be directly associated with retinal dystrophies by searching through the shortest paths in the skeleton graph (see workflow schema on Figure 1), instead of the immense number of interactions found in the complete whole network. RPGeNet does, however, allow users to expand beyond the skeleton subnetwork if needed. To make searches more practical, RPGeNet expands the network by levels until recreating the entire known interactions graph (whole graph). Each level is an expansion in the parents and children of the nodes in the previous level (see Figure 2), and its newly added nodes tend to be either less significant to the disease the higher the level you go or poorly studied genes with few known interactions.

**Figure 3:**
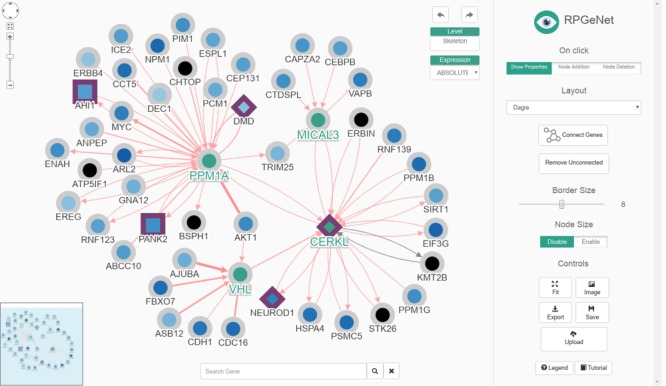
The renewed Network Explorer interface of RPGeNet. In this example, the Network Explorer interface shows a subgraph containing all the genes that directly interact with *CERKL* within the skeleton subnetwork, after expanding nodes for *PPM1A, VHL* and *MICAL3* (those selected four nodes highlighted in green, driver genes border in purple, node colors based on the ‘ABSOLUTE’ gene-expression data). Some improvements to the interface can be appreciated: a ‘search’ gene add-on at the mid-bottom, an expression data set selection drop-down menu at the right-top corner of the network visualization canvas, as well as the ‘undo’/‘redo’ buttons. A more dynamic ‘buttons panel’ on the right facilitates the interaction with the network data. Finally, the coloured-by-type interactions also provide directionality information with arrow heads and have reliability-score proportional widths adjusted to the number of evidences supporting them. This figure can be reproduced on the Network Explorer if users upload the Supplementary File 1.

**Figure 4:**
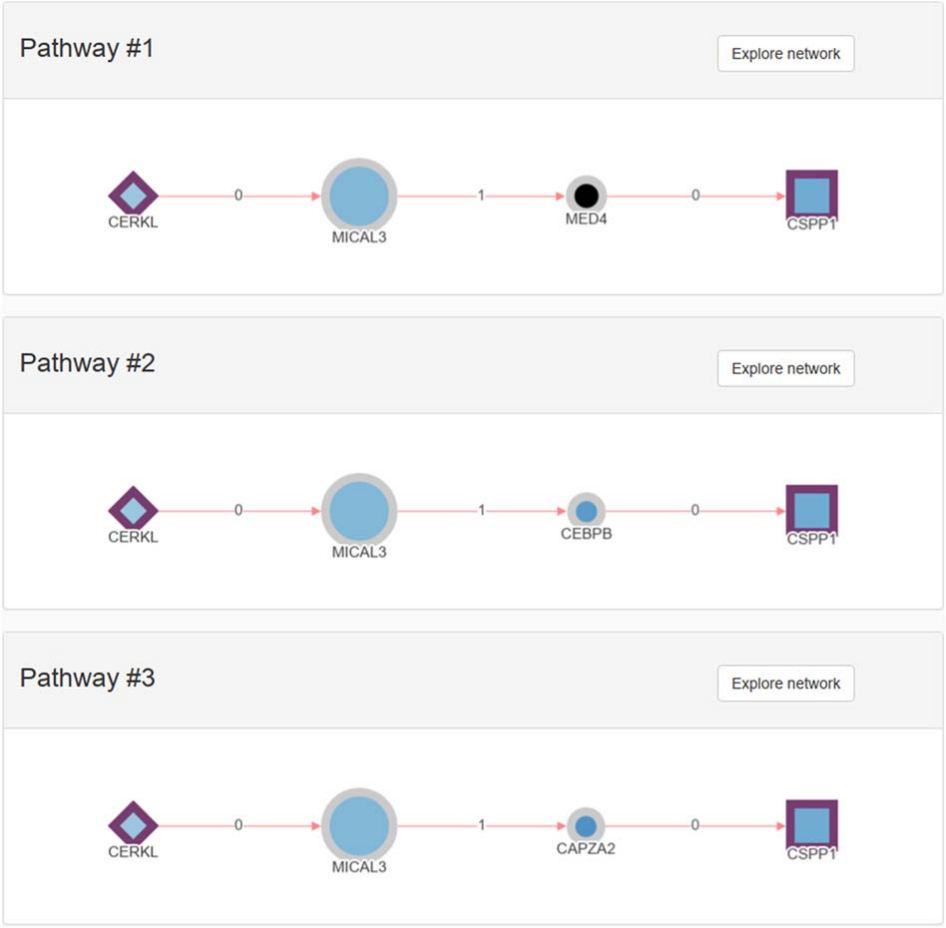
Example of a pathways list returned using the shortest pathway RPGeNet query. *CERKL* and *CSPP1* where used to start the pathway search on the main RPGeNet form. Only the first three pathways of the 28 retrieved by that query are shown on this figure, all of them at the shortest path length of three (three edges and two nodes between the chosen identifiers). By clicking on the corresponding ‘Explore Network’ button on any of the listed pathways, users can easily jump to the Network Explorer interface to work on the selected genes for that pathway.

RPGeNet now has three distinct types of queries available to undergo such disease-specific pathways characterization. The first query, as in the original RPGeNet, ‘is *what interacts directly with the gene of interest?*' The user sets one or more gene of interest, and the output is a graph with the gene or genes of interest connected with all the genes that interact directly. This graph can then be expanded, and its layout can be manually curated (Figure 3). The second query is ‘*what are all the shortest paths between two genes of interest?*' Two genes are provided, and a list of all the pathways between those two genes is returned (Figure 4). However, as edges in the pathway are directional, only pathways driving from the first requested gene to the second one are listed. To find possible pathways in the opposite direction, if any, the user should redo the query just switching the order of the query genes. A pathway can then be transferred to the Network Explorer where the user can further explore other nodes extending to the retrieved shortest pathway. A third query was included: *‘is a given gene connected to any disease associated gene?*' The idea is to characterize the shortest paths from a gene of interest, not yet related to a disease, to one of the network levels already defined over the driver genes. The new database engine facilitates that kind of query on the whole graph of interactions with respect to any of the nodes on the skeleton path and upper levels. Like in the previous query, a user can further explore a retrieved pathway from results listing on Network Explorer.

## Changes to database

### Driver genes

RPGeNet sources its driver genes from online database RetNet (1), which has a collection of over 300 mapped loci that are clinically validated with known Mendelian mutations that can cause a retinal disease in humans. Of the over 300 mapped loci, only the 276 identified genes, from now on referred to as driver genes, were chosen to build the skeleton network. That means that the database now handles 166 more driver genes than in the previous version. The increase of driver genes comes with many changes to the network including a more connected whole network and an earlier saturation of the interaction subnetworks when considering parent and child nodes growing from the skeleton network (see Supplementary Table 1 for a summary of graph statistics at different RPGeNet levels). Previously, the subnetworks saturated at level four and now saturate at level three. That posed some limitations to the previous implementation of the interface and the management of the network queries, which has been overcome with the new release of RPGeNet. All gene identifiers included on the whole network were unaliased to the official HUGO Gene Nomenclature Committee (HGNC) reference symbol (3).

### Data sources

*BioGRID*: (4) The RPGeNet v2.0 database has been updated with the interaction data from version 3.5.171 of BioGRID. This database contains a compilation of protein-to-protein interactions and genetic interaction data for about 61 species. BioGRID includes interactions of artificially induced trans-species. All non-human interactions, including interactions that had human proteins/genes that interacted with proteins/genes of another species, were filtered out. The protein-to-protein interactions are considered physical interactions, while the genetic interactions may refer to both physical and genetic interactions. Not all interactions had the physical/genetic label, but all interactions had an experiment type, hence it was possible to deduce the interaction type from the experiment in such cases. Examples of physical interaction experiments include affinity capture-luminescence, affinity capture-MS, co-crystal structure, FRET and two hybrid. Examples of genetic interaction experiments are dosage growth defect, dosage lethality and dosage rescue. All the filtering and curation were done by means of a Perl script that recovered for the whole network 15 139 nodes and 623 659 interactions from BioGRID (see Supplementary Tables 2, 3 and 4, for a comparison of the contribution made by each source database integrated into RPGeNet graph). The database, however, is filled with many undirected interactions. When direction of interaction is unknown or otherwise unstated, we assume bidirectionality. Our interactions graph building program took interactions with unknown direction and duplicate the interaction in the reverse direction (A**⇌**B will become A**⇀**B and B**⇀**A). For genetic interactions, the interactions were assumed to be unidirectional.

*STRING*: (5) The network was updated with interactions from version 11.0 of STRING. The top five sources for STRING were GRID, INTACT, KEGG, BIOCARTA and REACTOME (Supplementary Figure 1). Not all the interactions from this database have experimental evidence to back them up and many interactions are predictions of possible interactions. Because of this, any interaction not supported by evidence were discarded when building the RPGeNet core network. STRING database includes tags stating the directionality of the interaction; in the case that there is an interaction where the direction is not known, the interaction is assumed to be bidirectional. It also includes a large list of non-human protein interactions; those were filtered out as well. After the processing steps, 13 269 nodes and 629 271 interactions were included from this database (further details on Supplementary Tables 2, 3 and 4).

*PPaxe*: (6) This text-mining tool can sift through academic papers to find interactions for the user’s gene(s) of interest. PPaxe uses the random forest classifier algorithm, which is a machine learning method by which large collection of decorrelated decision trees are computed. PPaxe uses decision trees that combine different variables about the sentences it reads. One such variable that PPaxe considers is whether a verb describes the act of interaction or relationship. PPaxe was used to gather further interactions from scientific literature that were described and detected in published articles referred from PubMed. PPaxe replaces the sparser tool applied on the first release of RPGeNet with the added benefit of using machine learning to gather a larger number of interactions than possible by hand. PPaxe can work on abstracts and full-text articles; the first option gathers interactions solely from the abstract, but can process more articles because abstracts are generally free to read; the second option looks for interactions from entire articles, which implies a smaller set of articles. Each option was used in two separate searches: the first search was built to process PubMed papers that contained any of the 276 driver genes (65 820 abstracts and 29 819 full papers retrieved); the second search was scanning any interaction related to retinitis pigmentosa (1124 abstracts and 502 full papers retrieved). All four PPaxe outputs were combined into one set and were then filtered by score and by the putative gene/proteins found (Supplementary Figure 2). Any interactions not having a gene identifier on the HGNC official nomenclature database (3) were filtered out using a Perl script. PPaxe does not infer yet directionality on retrieved interactions, so that bidirectionality is assumed. A total of 3062 nodes and 13 584 interactions were collected for the RPGeNet core network (further details on Supplementary Tables 2, 3 and 4).

### Database manager

In order to handle a larger driver gene skeleton network and an increased amount of interactions, as well as to facilitate new ways of querying the data elements for the web interface, we had to resort to a more suitable database manager. Neo4j (community 3.1.7) was chosen for that purpose because it is a graph-based database manager that uses the property graph model to store and access the network data efficiently using a set of graph function instead of simply storing information in tables, like those used in traditional relational database managers as MySQL, etc (7). This database manager is used by other interaction database web applications, such as REACTOME and PlanNET, for storing and managing large interaction data (8, 9). The property graph model uses nodes (the elements to store attributes/data of an entity) and relationships (relevant connections between nodes). Its native use of graph functions to query graph data makes neo4j an ideal system to store and manage information for all the network levels of RPGeNet, speeding up searches either complex or taking larger numbers of nodes into account.

## Web server improvements

### Queries and performance

As mentioned in the tool description, RPGeNet now has three distinct queries available to help users in finding genes or pathways of interest. Previously, RPGeNet would break when trying to access interactions at higher subnetwork levels, but the new RPGeNet engine can now handle searching and visualizing interactions from genes in the highest level with respect to the gene interest. The new database manager not only optimizes the searches and access of higher levels but also makes possible that the new queries implemented in the new RPGeNet web interface were feasible and can be done in a reasonable amount of time.

### Data management and visualization

**Figure 5:**
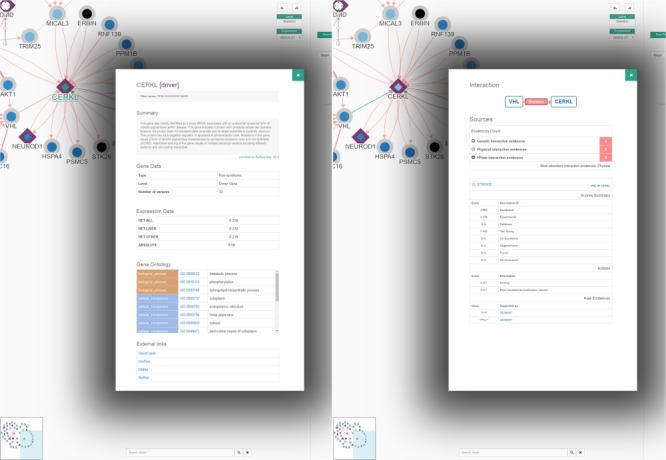
An example of node (left) and interaction (right) information panels from the Network Explorer interface. The default behaviour ‘*On click’* of the Network Explorer interface is to show ‘*node properties’* (see topmost controls on the right panel of that interface on previous figure). From the network example of Figure 3, when clicking at the *CERKL* node the gene/protein information panel pops up to display a description of the gene, known aliases, a summary of its expression levels and functional annotation and links to external references. On the other hand, by clicking on an edge, *VHL* to *CERKL* in this example, the interaction panel pops up, providing information about the type of possible interactions (genetic, blue; physical, red; or ‘unknown’, black), as well as a series of tables containing details about the supporting evidences from the distinct sources, along with the corresponding external links to the reference databases and to the supporting evidences when possible.

The current RPGeNet upgrade facilitates navigation through all data available, making it more accessible and producing more informative results. Cytoscape.js (10) was used to display the interactive graphs in RPGeNet (see Figure 3). The interactions between genes are now colour coded depending on what type of interaction exists between them (blue, for genetic; red, for physical; or black, for unknown edges). If multiple interaction types exist between two genes, multiple arrows with the corresponding interaction type colour will be drawn between the two genes. The driver genes also have distinctive shapes depending on whether they are associated with syndromic or non-syndromic retinitis pigmentosa, which is particularly useful for genetic diagnosis. Finally, clicking on a gene of interest provides users with further information about it from a pop-up panel like the one shown in Figure 5 (left panel). A basic summary of the gene is given: all of the known aliases, related expression data, functional annotation in Gene Ontology, the subnetwork level at which the gene is found within the RPGeNet network, the number of known variants and external links to its GeneCards, UniProt, OMIM and RetNet pages to access further information if needed. When users click on a given interaction, a complete information panel is also provided that summarizes all the evidences supporting that edge as well as links to the external references if available (see Figure 5, right panel).

### Gene expression layer

RPGeNet continues to use the NCBI GEO (11) entry GSE7905 (12) as an example of expression data that can be projected into the network. For this expression set there are several precomputed analyses available, like retina only absolute expression, retina fold-change versus all other tissues and fold-change with respect to liver on the same microarray experiment. However, the new implementation facilitates the integration of further expression datasets, some of them are already in progress, and we expect to make them available soon. Another improvement made on the network explorer interface is that charging expression data on the current visualized network can be done on the fly, without having to repeat the query as it happened in the previous version.

### Other improvements on the web interface

RPGeNet network explorer now has an ‘undo’ and a ‘redo’ buttons, recording up to five changes made to the graph. The ‘undo/redo’ buttons facilitate exploring the network with the already available ‘add/remove’ buttons too. Another add-on to the visualization interface is a ‘search’ bar that can look for any gene(s) and highlight them in the displayed graph, which is especially helpful when working on large graphs in the network explorer. There were also improvements made to the ‘save image’ and ‘save graph’ buttons to reduce the number of steps required, now the user is asked for the saving directory directly. One main improvement has been introduced to the ‘save graph’ button, which was initially only saving the nodes identity but not the nodes distinct locations within the graph so, when reuploading the graph to RPGeNet, the nodes were not necessarily laid out in the same way as in the previous session. When importing a graph, now the nodes are laid out exactly as in the previous session, facilitating the storage of manual rearrangements made by users across different work sessions.

## Discussion

Using open-source databases, like BioGRID and STRING, has the advantage that they are free and commonly used within the scientific community. The problem with these large databases is that they are usually too large to serve the community for specific necessities and need to be further curated by researchers to distil the relevant biological network data from noise. BioGRID has experimental evidence backing every interaction in their database. STRING, on the other hand, has many predicted interactions, which can be a good start for researchers interested in finding novel evidences for them. STRING and BioGRID share many of the same interactions; although the raw STRING database does have more interactions due to the predictions and the larger number of species in comparison to BioGRID. Despite STRING not having all of their interactions experimentally backed up, they do offer a much wider range of information for each interaction than does BioGRID. Once the STRING dataset was processed, all interactions that did not have experimental evidence were removed. Since the interactions without evidences were excluded, the new RPGeNet has a smaller whole graph than the previous version of RPGeNet that was also considering the predictions. We now have 18 542 nodes and 1 218 032 edges—defining 613 319 non-redundant interactions—versus the 63 139 nodes and 1 688 656 edges in the core network of the past version of RPGeNet.

On the other hand, using the PPaxe machine learning software, we managed to find multiple interactions but still required some post-filtering to ensure that all the interactions found were indeed protein/genetic interactions. PPaxe cannot distinguish yet between a genetic and a protein interaction, so all PPaxe derived interactions in the network were labelled as ‘unknown’ interactions (and coloured in black to distinguish from the other interactions). PPaxe retrieves the PubMed ID (PMID) of the article from which the interaction was found; those PMIDs are now available on RPGeNet, so that those who may be interested can figure out whether the ‘unknown’ interaction of interest describes a protein or a genetic interaction by jumping to the corresponding PubMed entry. Regardless of the cons, PPaxe is simpler to use and is able to retrieve interactions without defining any syntactic pattern, unlike the previous sparser method.

**Figure 6:**
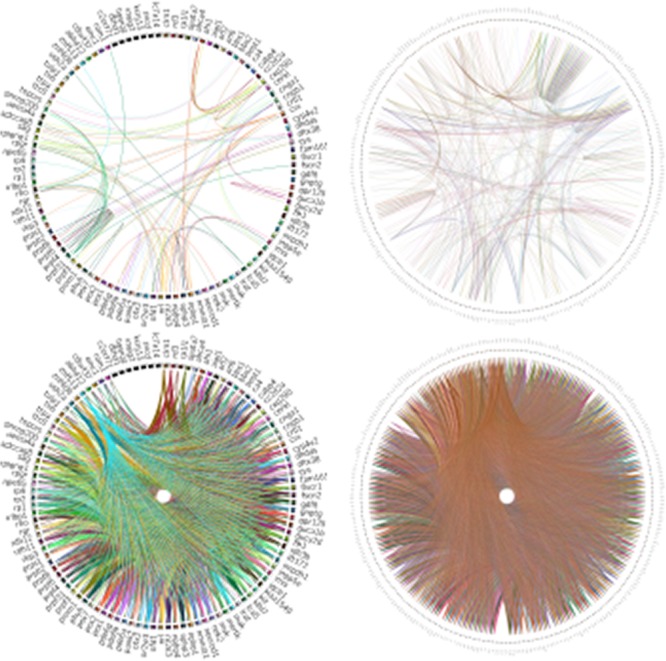
Analysis of RPGeNet v1 and v2 networks connectivity with Circos (19). The figure compares the connectivity of the driver genes of the old RPGeNet v1.0 (left) and the updated RPGeNet v2.0 (right). The top pair of the plots shows all the shortest paths between driver genes at distance one, meaning direct interaction between each pair of driver genes. The bottom part of the plots provide the comparison at distance three, meaning there are two genes in the shortest path between a pair of driver genes of interest. It is clear from the visualized Circos plots that the updated RPGeNet database has a highly connected network.

In relation to the updated core whole network, shortest paths between two retinitis pigmentosa driver genes had distances from one to seven, where a distance of one means that there is a direct interaction between two driver genes and any number above one is the number of genes in between the two driver genes that made up the shortest pathway. The shortest path distance for all 276 driver genes fall within three to four edges; in other words, there are two to three connecting genes between them (Figure 6). The subnetworks can be compared by the topology of each of the level’s own graph (see Supplementary Table 1). The average degree is 17.68 in the skeleton (~8.84 in-/out-degree), 104.458 in level one (~52.23 in−/out-) and 131.49 in level three (~65.74 in-/out-). The large increase of average degree for level one mainly results from both adding new nodes and basically much more interactions; obviously, network saturates faster at nodes than at interactions (about 4.44-fold and 26.24-fold increase from skeleton, respectively, but accounting for 96.27% of nodes and 76.54% of edges from whole graph). Such trend can be observed on the corresponding in-/out-degree density graphs (see Supplementary Figure 3, as well as on the aforementioned Supplementary Table 1).

With an increased number of driver genes, it was expected for the RPGeNet core whole network to swell immensely. Previously, the network saturated at subnetworks of level four meaning that there were no more interactions within the network above that level. The only genes not found within these four subnetwork levels were genes that were unconnected to the rest of the network. The new RPGeNet network saturates earlier at level three. There is a decrease in the number of total nodes and interactions in relation to the previous version, but there is also a large number of supporting evidences for interactions that did not exist in the previous network. Most of the nodes and interactions no longer included are the result of stricter filtering of the data from the STRING database and improved anti-aliasing of node identifiers over HGNC standard symbols; only interactions with evidence were added to the network and predictions were left out. It is possible that these new nodes and interactions filled up missing gaps in the network, sketching new pathways to be discovered.

**Figure 7:**
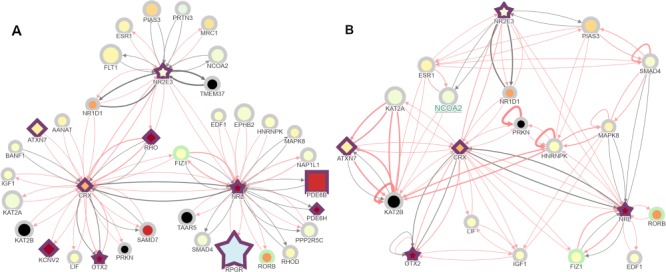
A control case interaction visualized on RPGeNet showing the connections between *NRL*, *NR2E3* and *CRX* retinal transcription factors. (A) RPGeNet was queried to display the subnetwork among *NRL*, *NR2E3* for the nodes at distance one at level one; then nodes connected to *CRX* were added with the ‘*Node Addition’* button activated on click. *CRX*, *NRL* and *NR2E3* are three well-known transcription factors that co-regulate retinal-specific genes, among them *RHO*. Interestingly, several other genes that cause retinal dystrophies also appear in the network as target genes or other transcriptional regulators (border shown in purple). Nodes color-fill defined by the ‘RET-ALL’ gene-expression data. (B) Further trimming of the subnetwork obtained by omitting the nodes that are not chromatin remodelers or transcription factors provide an overview of relevant co-regulators of retinal genes. After deleting the corresponding nodes, further edges—out of the shortest paths that link the genes left—were shown by clicking on the ‘*Connect Genes’* button at the control panel. This visualization allows pinpointing and exploring alternate pathways that connect the initial seeds; for instance, *CRX* and *NRL* were already connected, but longer paths are now evident like *CRX*⇌*PRKN*⇌*HNRNPK*⇌*NRL*, when *PRKN* and *HNRNPK* are linked. Another example can be the pathway found between *NRL* and *NR2E3* on the path *NRL*⇌*SMAD4*⇌*PIAS3*⇌*NR2E3*, when *SMAD4* and *PIAS3* are linked. Both panels from this figure can be reproduced on the Network Explorer if users upload the Supplementary Files 2 and 3, respectively.

Even with an earlier saturation at level three, there were still driver genes that did not connect to any other member of the network. Some of the unconnected driver genes within the network were mitochondrial genes, like *MT-ND4*, *MT-TP* and *MT-TS2*. This may be because there is not enough research on interactions between mitochondrial genes/proteins and autosomal genes/proteins despite the fact that clear genetic communication between the nucleus and the mitochondria is known (13), and that most proteins of the mitochondria are, in fact, encoded in the autosomal DNA (14). Yet the proteins that are encoded in mitochondrial DNA are all important for the electron transport chain and connect well with each other (15,16). There were also few autosomal genes that did not connect to the network but that may simply be because they do not have any known interactions at the moment or they have not been characterized at an experimental level in depth (see Supplementary Tables 5 and 6 for a list of driver and non-driver genes, respectively, not connected to the core interactions network).

The RPGeNet network was curated by reducing the network to the shortest paths between known driver genes, allowing users to better identify genes and pathways important in the development of retinitis pigmentosa. Using RPGeNet, many potential candidate genes have been identified by inspecting the shortest paths found in the skeleton graph. One of the candidate genes identified in the skeleton, *SIRT1*, has recently been experimentally confirmed to interact with *CERKL*. More importantly, it was found that *CERKL* regulates autophagy via *SIRT1* (17). This discovery supports *SIRT1* as a new driver gene of retinitis pigmentosa and confirms the utility of the RPGeNet model to identify potential IRD candidate genes. Furthermore, RPGeNet allows to visualize and to highlight new connections even in known interaction networks. The subnetwork retrieved after querying for three retinal-specific transcription factors (shortest path between *NRL* and *NR2E3*, plus addition of *CRX*) allows showing their connection to other causative retinal dystrophy genes (Figure 7A). In addition, such subnetwork can be easily trimmed by omitting ‘noisy’ nodes to focus on particular interactors. In this case, deletion of the nodes unrelated to transcriptional regulation and chromatin remodelers unveils new regulatory loops between these transcription factors that may be relevant for retinal development and maintenance (Figure 7B).

There are plans on the way to create a mouse and zebrafish RPGeNet specific interaction networks, as they are the two most used model organisms for research on retinal dystrophies, and later on to integrate them with the human network currently available. The newer RPGeNet graph engine will facilitate clustering gene nodes against a separate network layer based on disease nodes; for instance, as described in Lázaro-Guevara *et al*. (18). New retinal differential gene-expression data from new RNA-seq and proteomic experiments is under analysis and will be added soon. We are also working on an automated pipeline to automate the protocol used to create RPGeNet, so it would be easier to keep it up-to-date as well as to expand the procedure to generate specific interaction networks for other rare diseases.

## Availability

RPGeNet is an open-source refined interaction network for retinitis pigmentosa. The RPGeNet website provides data description and a complete tutorial. Visit RPGeNet at https://compgen.bio.ub.edu/RPGeNet.

## Supplementary Material

Supplementary_data_baz120
