# Supplementary material for: RPGeNet v2.0: expanding the universe of retinal disease gene interactions network
Source: Database (Oxford). 2019 Nov 11;2019:baz120. doi: 10.1093/database/baz120 (PMC6846243; doi:10.1093/database/baz120)
Supplement: Supplementary_data_baz120 [file supplementary_data_baz120.zip › DATABASE-2019-0032.R2_Proof_hi_maindocwithoutfigures_baz120.pdf]

OXFORD UNIVERSITY PRESS | DATABASE

RPGeNet v2.0: expanding the universe of retinal disease gene interactions network.

Journal:	*DATABASE*
Manuscript ID	DATABASE-2019-0032.R2
Manuscript Type:	Database Update
Date Submitted by the Author:	n/a
Complete List of Authors:	Arenas-Galnares, Rodrigo; Universitat de Barcelona, Genetics, Microbiology and Statistics; Institut de Biomedicina Universitat de Barcelona<br>Castillo-Lara, Sergio; Universitat de Barcelona, Genetics, Microbiology and Statistics; Institut de Biomedicina Universitat de Barcelona<br>Toulis, Vasileios; Universitat de Barcelona, Genetics, Microbiology and Statistics; Institut de Biomedicina Universitat de Barcelona; CIBERER ISCII<br>Boloc, Daniel; Universitat de Barcelona Facultat de Medicina, Medicine<br>González-Duarte, Roser; DBGen Ocular Genomics<br>Marfany, Gemma; Universitat de Barcelona, Genetics, Microbiology and Statistics; Institut de Biomedicina Universitat de Barcelona; CIBERER ISCII; DBGen Ocular Genomics<br>Abril, Josep; Universitat de Barcelona, Genetics, Microbiology and Statistics; Institut de Biomedicina Universitat de Barcelona
Keywords:	retinal diseases, interactome network, browser, pathway analysis and curation

Note: The following files were submitted by the author for peer review, but cannot be converted to PDF. You must view these files (e.g. movies) online.

SFile1_RPGeNet_Upgrade_2019_Database_Figure3.json
SFile2_RPGeNet_Upgrade_2019_Database_Figure7A.json
SFile3_RPGeNet_Upgrade_2019_Database_Figure7B.json

SCHOLARONE™ Manuscripts

RPGeNet v2.0: expanding the universe of retinal disease gene interactions network

Rodrigo Arenas-Galnares[1,2,✓], Sergio Castillo-Lara[1,2,✓], Vasileios Toulis[1,2,3], Daniel Boloc[4], Roser Gonzàlez-Duarte[5], Gemma Marfany[1,2,3,5,*,+] Josep F. Abril[1,2,*,+]

[1] Department of Genetics, Microbiology and Statistics, University of Barcelona, Barcelona, 08028, Catalonia, Spain
[2] Institute of Biomedicine (IBUB), University of Barcelona, Barcelona, 08028, Catalonia, Spain
[3] CIBERER, ISCIII, University of Barcelona, Barcelona, 08028, Catalonia, Spain
[4] Faculty of Medicine, University of Barcelona, Barcelona, 08036, Catalonia, Spain
[5] DBGen Ocular Genomics, Barcelona, 08028, Catalonia, Spain

✓ The authors wish it to be known that, in their opinion, the first two authors should be regarded as joint First Authors.
* To whom correspondence should be addressed. Telf: +34 93 403 1305. Email: jabril@ub.edu
+ The authors wish it to be known that last two authors should be regarded as joint Senior Authors.

ABSTRACT

RPGeNet offers researchers a user-friendly queriable tool to visualize the interactome network of visual disorder genes, thus enabling the identification of new potential causative genes and the assignment of novel candidates to specific retinal or cellular pathways. This can be highly relevant for clinical applications as retinal dystrophies affect 1:3000 people worldwide and the causative genes are still unknown for 30% of the patients. RPGeNet is a refined interaction network interface that limits its skeleton network to the shortest paths between each and every known causative gene of inherited syndromic and non-syndromic retinal dystrophies. RPGeNet integrates interaction information from STRING, BioGRID, and PPaxe, along with retina-specific expression data and associated genetic variants, over a Cytoscape.js web interface. For the new version, RPGeNet v2.0, the database engine was migrated to Neo4j graph database manager, which speeds up the initial queries and can handle whole interactome data for new ways to query the network. Further user facilities have been introduced, as the capability of saving and restoring a researcher customized network layout, or as novel features to facilitate navigation and data projection on the network explorer interface. Responsiveness has been further improved by transferring some functionality to the client-side.

INTRODUCTION

Inherited retinal dystrophies (IRDs) comprise a highly heterogeneous group of disorders caused by over 200 causative genes (1). The prevalence of IRDs is 1:3000 worldwide, which make these blinding disorders a health relevant target. The implementation of massive sequencing approaches has greatly facilitated genetic testing and, as a result, the number of IRD genes and mutations is constantly increasing. Nonetheless, a substantial number of cases remain to be accurately diagnosed, as the average yield in IRD genetic diagnosis is roughly 50%.

Besides technical limitations, one of the bottlenecks in massive sequence-based molecular diagnosis is that most identified variants are previously unreported missense changes of unknown pathogenicity, either in known causative genes or in previously unreported candidates. These variants may be deemed as pathogenic or probably damaging by *in silico* predictive programs but end up classified as VUS (genetic variants of unknown significance), since there are not functional analyses to support their pathogenicity and their relationship to retinal physiology is yet to be determined.

Molecular medicine based on gene and protein networks is rapidly expanding since most disease-causing genes often work together, either forming a protein complex or participating in the same signalling pathways. In contrast to the analysis of isolated genes, finding the networks that link disease candidate genes provides supporting data for the identification of new causative genes, functional clues for assaying putative pathogenic novel variants, as well as opens new scenarios to identify key therapeutical targets.

Comprehensive tools to navigate through gene functions, cellular pathways and pathogenicity begin to emerge, particularly for cancer research. Although a considerable amount of genetic and functional data on IRDs genes and mutations has been gathered, there are not many user-friendly searchable tools to make a network map of gene/proteins interactions. To fill this gap, a web application, `RPGeNet` (2), was implemented that integrated all the physical and genetic interactions at that time for a subset of IRD genes (retinitis pigmentosa and Leber congenital amaurosis), obtained from different databases and including additional data such as tissue-specific expression. The expansion of genetic data, plus all the interactome and other omics information gathered of late, has prompted us to review and expand the initial 100 genes to more than 200 retinal dystrophy genes,

include distilled interaction databases and implement an improved network generation, management, and visualization interface.

TOOL DESCRIPTION

RPGeNet is a tool to assist in the search for potential candidate genes and pathways associated with retinitis pigmentosa and intended to be used by both research and genetic diagnostic purposes. RPGeNet refines the vast interaction network by reducing it to the shortest paths between known driver genes of the disease (from now on the so-called skeleton network). The reduced network allows users to better identify genes interconnecting drivers that can be directly associated with retinal dystrophies by searching through the shortest paths in the skeleton graph (see workflow schema on Figure 1), instead of the immense number of interactions found in the complete whole network. RPGeNet does, however, allow users to expand beyond the skeleton subnetwork if needed. To make searches more practical, RPGeNet expands the network by levels until recreating the entire known interactions graph (whole graph). Each level is an expansion in the parents and children of the nodes in the previous level (see Figure 2), and its newly added nodes tend to be either less significant to the disease the higher the level you go or poorly studied genes with few known interactions.

RPGeNet now has three distinct types of queries available to undergo such disease-specific pathways characterization. The first query, as in the original RPGeNet, is *what interacts directly with the gene of interest?* The user sets one or more gene of interest and the output is a graph with the gene or genes of interest connected with all the genes that interact directly. This graph can then be expanded and its layout manually curated (Figure 3). The second query is *what are all the shortest paths between two genes of interest?* Two genes are provided and a list of all the pathways between those two genes is returned (Figure 4). However, as edges in the pathway are directional, only pathways driving from the first requested gene to the second one are listed. To find possible pathways in the opposite direction, if any, the user should redo the query just switching the order of the query genes. A pathway can then be transferred to the Network Explorer where the user can further explore other nodes extending to the retrieved shortest pathway. A third query was included: *is a given gene connected to any disease associated gene?* The idea is to characterize the shortest paths from a gene of interest, not yet related to a disease, to one of the network levels already defined over the driver genes. The new database engine facilitates that kind of query on the whole graph of interactions

with respect to any of the nodes on the skeleton path and upper levels. Like in the previous query, a user can further explore a retrieved pathway from results listing on `Network Explorer`.

CHANGES TO DATABASE

Driver Genes

`RPGeNet` sources its driver genes from online database `RetNet` (1), which has a collection of over 300 mapped loci that are clinically validated with known Mendelian mutations that can cause a retinal disease in humans. Of the over 300 mapped loci only the 276 identified genes, from now on referred to as driver genes, were chosen to build the skeleton network. That means that the database now handles 166 more driver genes than in the previous version. The increase of driver genes comes with many changes to the network including a more connected whole network and an earlier saturation of the interaction subnetworks when considering parent and child nodes growing from the skeleton network (see Supplementary Table 1 for a summary of graph statistics at different `RPGeNet` levels). Previously, the subnetworks saturated at level four and now saturate at level three. That posed some limitations to the previous implementation of the interface and the management of the network queries, which has been overcome with the new release of `RPGeNet`. All gene identifiers included on the whole network were unaliased to the official HUGO Gene Nomenclature Committee (HGNC) reference symbol (3).

Data Sources

`BioGRID`: (4) The `RPGeNet` v2.0 database has been updated with the interaction data from version 3.5.171 of `BioGRID`. This database contains a compilation of protein-to-protein interactions and genetic interaction data for about sixty-one species. `BioGRID` includes interactions of artificially induced trans-species. All non-human interactions, including interactions that had human proteins/genes that interacted with proteins/genes of another species, were filtered out. The protein-to-protein interactions are considered physical interactions whilst the genetic interactions may refer to both physical and genetic interactions. Not all interactions had the physical/genetic label but all interactions had an experiment type, hence it was possible to deduce the interaction type from the experiment in such cases. Examples of physical interaction experiments include affinity capture-

luminescence, affinity capture-MS, co-crystal structure, FRET, and two-hybrid. Examples of genetic interaction experiments are dosage growth defect, dosage lethality, and dosage rescue. All the filtering and curation was done by means of a `Perl` script that recovered for the whole network 15,139 nodes and 623,659 interactions from `BioGRID` (see Supplementary Tables 2, 3, and 4, for a comparison of the contribution made by each source database integrated into `RPGeNet` graph). The database, however, is filled with many undirected interactions. When direction of interaction is unknown or otherwise unstated, we assume bidirectionality. Our interactions graph building program took interactions with unknown direction and duplicate the interaction in the reverse direction (A⇌B will become A⇀B and B⇀A). For genetic interactions, the interactions were assumed to be unidirectional.

STRING: (5) The network was updated with interactions from version 11.0 of `STRING`. The top five sources for `STRING` were `GRID`, `INTACT`, `KEGG`, `BIOCARTA` and `REACTOME` (Supplementary Figure 1). Not all the interactions from this database have experimental evidence to back them up and many interactions are predictions of possible interactions. Because of this, any interaction not supported by evidence were discarded when building the `RPGeNet` core network. `STRING` database includes tags stating the directionality of the interaction; in the case that there is an interaction where the direction is not known, the interaction is assumed to be bidirectional. It also includes a large list of non-human protein interactions; those were filtered out as well. After the processing steps, 13,269 nodes and 629,271 interactions were included from this database (further details on Supplementary Tables 2, 3, and 4).

PPaxe: (6) This text-mining tool can sift through academic papers to find interactions for the user's gene(s) of interest. `PPaxe` uses the random forest classifier algorithm, which is a machine learning method by which large collection of decorrelated decision trees are computed. `PPaxe` uses decision trees that combine different variables about the sentences it reads. One such variable that `PPaxe` considers is whether a verb describes the act of interaction or relationship. `PPaxe` was used to gather further interactions from scientific literature that were described and detected in published articles referred from `PubMed`. `PPaxe` replaces the `sparser` tool applied on the first release of `RPGeNet` with the added benefit of using machine learning to gather a larger number of interactions than possible by hand. `PPaxe` can work on abstracts and full text articles; the first option gathers interactions solely from the abstract, but can process more articles because abstracts are generally free to read; the

second option looks for interactions from entire articles, which implies a smaller set of articles. Each option was used in two separate searches: the first search was built to process PubMed papers that contained any of the 276 driver genes (65,820 abstracts and 29,819 full papers retrieved); the second search was scanning any interaction related to retinitis pigmentosa (1,124 abstracts and 502 full papers retrieved). All four PPaxe outputs were combined into one set and were then filtered by score and by the putative gene/proteins found (Supplementary Figure 2). Any interactions not having a gene identifier on the HGNC official nomenclature database (3) were filtered out using a Perl script. PPaxe does not infer yet directionality on retrieved interactions, so that bidirectionality is assumed. 3,062 nodes and 13,584 interactions were collected for the RPGeNet core network (further details on Supplementary Tables 2, 3, and 4).

Database Manager

In order to handle a larger driver gene skeleton network and an increased amount of interactions, as well as to facilitate new ways of querying the data elements for the web interface, we had to resort to a more suitable database manager. Neo4j (community 3.1.7) was chosen for that purpose because it is a graph-based database manager that uses the property graph model to store and access the network data efficiently using a set of graph function instead of simply storing information in tables, like those used in traditional relational database managers as MySQL, etc (7). This database manager is used by other interaction database web applications, such as REACTOME and PlanNET, for storing and managing large interaction data (8,9). The property graph model uses nodes (the elements to store attributes/data of an entity) and relationships (relevant connections between nodes). Its native use of graph functions to query graph data makes neo4j an ideal system to store and manage information for all the network levels of RPGeNet, speeding up searches either complex or taking larger numbers of nodes into account.

WEB SERVER IMPROVEMENTS

Queries and Performance

As mentioned in the tool description, RPGeNet now has three distinct queries available to help users in finding genes or pathways of interest. Previously, RPGeNet would break when trying to access

interactions at higher subnetwork levels, but the new RPGeNet engine can now handle searching and visualizing interactions from genes in the highest level with respect to the gene interest. The new database manager not only optimizes the searches and access of higher levels, but also makes possible that the new queries implemented in the new RPGeNet web interface were feasible and can be done in a reasonable amount of time.

Data Management and Visualization

The current RPGeNet upgrade facilitates navigation through all data available, making it more accessible and producing more informative results. Cytoscape.js (10) was used to display the interactive graphs in RPGeNet (see Figure 3). The interactions between genes are now colour coded depending on what type of interaction exists between them (in blue for genetic, red for physical or black for unknown edges, respectively). If multiple interaction types exist between two genes, multiple arrows with the corresponding interaction type colour will be drawn between the two genes. The driver genes also have distinctive shapes depending on whether they are associated with syndromic or non-syndromic retinitis pigmentosa, which is particularly useful for genetic diagnosis. Finally, clicking on a gene of interest provides users with further information about it from a pop-up panel like the one shown in Figure 5 (left panel).. A basic summary of the gene is given: all of the known aliases, related expression data, functional annotation in Gene Ontology (GO), the subnetwork level at which the gene is found within the RPGeNet network, the number of known variants, and external links to its GeneCards, UniProt, OMIM, and RetNet pages to access further information if needed. When users click on a given interaction, a complete information panel is also provided that summarizes all the evidences supporting that edge as well as links to the external references if available (see Figure 5, right panel)

Gene Expression Layer

RPGeNet continues to use the NCBI GEO (11) entry GSE7905 (12) as an example of expression data that can be projected into the network. For this expression set there are several precomputed analyses available, like retina only absolute expression, retina fold-change versus all other tissues, and fold-change with respect to liver on the same microarray experiment. However, the new

implementation facilitates the integration of further expression datasets, some of them are already in progress and we expect to make them available soon. Another improvement made on the network explorer interface is that charging expression data on the current visualized network can be done on the fly, without having to repeat the query as it happened in the previous version.

Other Improvements on the Web Interface

RPGeNet network explorer now has an “undo” and a “redo” buttons, recording up to five changes made to the graph. The “undo/redo” buttons facilitate exploring the network with the already available “add/remove” buttons too. Another add-on to the visualization interface is a “search” bar that can look for any gene(s) and highlight them in the displayed graph; which is especially helpful when working on large graphs in the network explorer. There were also improvements made to the “save image” and “save graph” buttons to reduce the number of steps required; now the user is asked for the saving directory directly. One main improvement has been introduced to the “save graph” button, which was initially only saving the nodes identity but not the nodes distinct locations within the graph so, when reuploading the graph to RPGeNet, the nodes were not necessarily laid out in the same way as in the previous session. When importing a graph, now the nodes are laid out exactly as in the previous session, facilitating the storage of manual rearrangements made by users across different work sessions.

DISCUSSION

Using open-source databases, like BioGRID and STRING, has the advantage that they are free and commonly used within the scientific community. The problem with these large databases is that they are usually too large to serve the community for specific necessities and need to be further curated by researchers to distil the relevant biological network data from noise. BioGRID, has experimental evidence backing every interaction in their database. STRING, on the other hand, has many predicted interactions, which can be a good start for researchers interested in finding novel evidences for them. STRING and BioGRID share many of the same interactions, although the raw STRING database does have more interactions due to the predictions and the larger number of species in comparison to BioGRID. Despite STRING not having all of their interactions experimentally

backed up, they do offer a much wider range of information for each interaction than does BioGRID. Once the STRING dataset was processed, all interactions that did not have experimental evidence were removed. Since the interactions without evidences were excluded, the new RPGeNet has a smaller whole graph than the previous version of RPGeNet that was also considering the predictions. We now have 18,542 nodes and 1,218,032 edges—defining 613,319 non-redundant interactions—versus the 63,139 nodes and 1,688,656 edges in the core network of the past version of RPGeNet.

On the other hand, using the PPaxe machine learning software, we managed to find multiple interactions, but still required some post-filtering to ensure that all the interactions found were indeed protein/genetic interactions. PPaxe cannot distinguish yet between a genetic and a protein interaction, so all PPaxe derived interactions in the network were labelled as "unknown" interactions (and coloured in black to distinguish from the other interactions). PPaxe retrieves the PubMed ID (PMID) of the article from which the interaction was found; those PMIDs are now available on RPGeNet, so that those who may be interested can figure out whether the “unknown” interaction of interest describes a protein or a genetic interaction by jumping to the corresponding PubMed entry. Regardless of the cons, PPaxe is simpler to use and is able to retrieve interactions without defining any syntactic pattern, unlike the previous sparser method.

In relation to the updated core whole network, shortest paths between two retinitis pigmentosa driver genes had distances from one to seven, where a distance of one means that there is a direct interaction between two driver genes and any number above one is the number of genes in between the two driver genes that made up the shortest pathway. The shortest paths distances for all 276 driver genes fall within three to four edges; in other words there are two to three connecting genes between them (Figure 6). The subnetworks can be compared by the topology of each of the level’s own graph (see Supplementary Table 1). The average degree is 17.68 in the skeleton (~8.84 in-/out-degree), 104.458 in level one (~52.23 in-/out-), and 131.49 in level three (~65.74 in-/out-). The large increase of average degree for level one mainly results from both adding new nodes and basically much more interactions; obviously, network saturates faster at nodes than at interactions (about 4.44 fold and 26.24 fold increase from skeleton, respectively, but accounting for 96.27% of nodes and 76.54% of edges from wholegraph). Such trend can be observed on the corresponding in-/out-degree

density graphs (see Supplementary Figure 3, as well as on the aforementioned Supplementary Table 1).

With an increased number of driver genes, it was expected for the RPGeNet core whole network to swell immensely. Previously, the network saturated at subnetworks of level four meaning that there were no more interactions within the network above that level. The only genes not found within these four subnetwork levels were genes that were unconnected to the rest of the network. The new RPGeNet network saturates earlier at level three. There is a decrease in the number of total nodes and interactions in relation to the previous version, but there is also a large number of supporting evidences for interactions that did not exist in the previous network. Most of the nodes and interactions no longer included are the result of stricter filtering of the data from the STRING database and improved anti-aliasing of node identifiers over HGNC standard symbols; only interactions with evidence were added to the network and predictions were left out. It is possible that these new nodes and interactions filled up missing gaps in the network, sketching new pathways to be discovered.

Even with an earlier saturation at level three, there were still driver genes that did not connect to any other member of the network. Some of the unconnected driver genes within the network were mitochondrial genes, like *MT-ND4*, *MT-TP*, *MT-TS2*. This may be because there is not enough research on interactions between mitochondrial genes/proteins and autosomal genes/proteins despite the fact that clear genetic communication between the nucleus and the mitochondria is known (13) and that most proteins of the mitochondria are, in fact, encoded in the autosomal DNA (14). Yet the proteins that are encoded in mitochondrial DNA are all important for the electron transport chain and connect well with each other (15,16). There were also few autosomal genes that did not connect to the network but that may simply be because they do not have any known interactions at the moment or they have not been characterized at an experimental level in depth (see Supplementary Tables 5 and 6 for a list of driver and non-driver genes, respectively, not connected to the core interactions network).

The RPGeNet network was curated by reducing the network to the shortest paths between known driver genes, allowing users to better identify genes and pathways important in the development of retinitis pigmentosa. Using RPGeNet many potential candidate genes have been identified by inspecting the shortest paths found in the skeleton graph. One of the candidate genes identified in the

skeleton, *SIRT1*, has recently been experimentally confirmed to interact with *CERKL*. More importantly, it was found that *CERKL* regulates autophagy via *SIRT1* (17). This discovery supports *SIRT1* as a new driver gene of retinitis pigmentosa and confirms the utility of the `RPGeNet` model to identify potential IRD candidate genes. Furthermore, `RPGeNet` allows to visualize and to highlight new connections even in known interaction networks. The subnetwork retrieved after querying for three retinal-specific transcription factors (shortest path between *NRL* and *NR2E3*, plus addition of *CRX*), allows showing their connection to other causative retinal dystrophy genes (Figure 7A). In addition, such subnetwork can be easily trimmed by omitting "noisy" nodes to focus on particular interactors. In this case, deletion of the nodes unrelated to transcriptional regulation and chromatin remodelers unveils new regulatory loops between these transcription factors that may be relevant for retinal development and maintenance (Figure 7B).

There are plans on the way to create a mouse and zebrafish `RPGeNet` specific interaction networks, as they are the two most used model organisms for research on retinal dystrophies, and later on to integrate them with the human network currently available. The newer `RPGeNet` graph engine will facilitate clustering gene nodes against a separate network layer based on disease nodes; for instance, as described in Lázaro-Guevara *et al* (18). New retinal differential gene-expression data from new RNA-seq and proteomic experiments is under analysis and will be added soon. We are also working on an automated pipeline to automate the protocol used to create `RPGeNet`, so it would be easier to keep it up-to-date as well as to expand the procedure to generate specific interaction networks for other rare diseases.

AVAILABILITY

`RPGeNet` is an open-source refined interaction network for retinitis pigmentosa. The `RPGeNet` website provides data description and a complete tutorial. Visit `RPGeNet` at https://compgen.bio.ub.edu/RPGeNet

SUPPLEMENTARY DATA

Supplementary Data are available at “`Database`” online.

ACKNOWLEDGEMENTS

This work was supported by research grants from BFU2017-83755P (Spanish Ministry of Economy), and 2017-SGR-1455 (Generalitat de Catalunya) to JFA; and SAF2016-80937-R (Ministerio de

Economía y Competitividad/FEDER), 2017 SGR 738 (Generalitat de Catalunya), and La Marató TV3 (Project Marató 201417-30-31-32) to GM and RGD. S.C.-L. is a fellow of the Catalan Government 'AGAUR' (FI- FDR, 2017FI_B_00191). V.T. is fellow of the MINECO (BES-2014-068639, Ministerio de Economía, Industria y Competitividad).

CONFLICT OF INTEREST

G.M. and R.G-D. are co-founders and assessors of DBGen, a spin-off of the Universitat de Barcelona dedicated to the genetic diagnosis of visual disorders.

The authors declare there is not a competing interest.

REFERENCES

1. Daiger, S. P.; Sullivan, L. S. and Bowne, S. J. RetNet, the Retinal Information Network https://sph.uth.edu/retnet/
2. Boloc, D.; Castillo-Lara, S.; Marfany, G.; Gonzàlez-Duarte, R. and Abril, J. F. (2015) Distilling a Visual Network of Retinitis Pigmentosa Gene-Protein Interactions to Uncover New Disease Candidates, *PLoS One*, **10**, e0135307. PMID:25952370
3. Braschi, B.; Denny, P.; Gray, K.; Jones, T.; Seal, R.; Tweedie, S.; Yates, B. and Bruford, E. (2019) Genenames.org: the HGNC and VGNC resources in 2019., *Nucleic Acids Res.*, **47**, D786–D792. PMID:30304474
4. Chatr-Aryamontri, A.; Oughtred, R.; Boucher, L.; Rust, J.; Chang, C.; Kolas, N. K.; O'Donnell, L.; Oster, S.; Theesfeld, C.; Sellam, A.; Stark, C.; Breitkreutz, B. J.; Dolinski, K. and Tyers, M. (2017) The BioGRID interaction database: 2017 update, *Nucleic Acids Res.*, **45**, D369–D379. PMID:27980099
5. Szklarczyk, D.; Morris, J. H.; Cook, H.; Kuhn, M.; Wyder, S.; Simonovic, M.; Santos, A.; Doncheva, N. T.; Roth, A.; Bork, P.; Jensen, L. J. and von Mering, C. (2017) The STRING database in 2017: quality-controlled protein-protein association networks, made broadly accessible., *Nucleic Acids Res.*, **45**, D362–D368. PMID:27924014
6. Castillo-Lara, S. and Abril, J. F. (2018) PPaxe: easy extraction of protein occurrence and interactions from the scientific literature., *Bioinformatics*, bty988 [Epub ahead of print]. PMID:30500875
7. Robinson, I.; Webber, J. and Eifrem, E. *Graph Databases: New opportunities for connected data.*; O'Reilly Media, Inc., 2014
8. Fabregat, A.; Jupe, S.; Matthews, L.; Sidiropoulos, K.; Gillespie, M.; Garapati, P.; Haw, R.; Jassal, B.; Korninger, F.; May, B.; Milacic, M.; Roca, C. D.; Rothfels, K.; Sevilla, C.; Shamovsky, V.; Shorser, S.; Varusai, T.; Viteri, G.; Weiser, J.; Wu, G.; Stein, L.; Hermjakob, H. and D'Eustachio, P. (2018) The Reactome Pathway Knowledgebase, *Nucleic Acids Res.*, **46**, D649–D655. PMID:29145629
9. Castillo-Lara, S. and Abril, J. F. (2018) PlanNET: homology-based predicted interactome for multiple planarian transcriptomes., *Bioinformatics*, **34**, 1016–1023. PMID:29186384
10. Franz, M.; Lopes, C. T.; Huck, G.; Dong, Y.; Sumer, O. and Bader, G. D. (2016) Cytoscape.js: a graph theory library for visualisation and analysis., *Bioinformatics*, **32**, 309–11. PMID:26415722
11. Barrett, T.; Wilhite, S. E.; Ledoux, P.; Evangelista, C.; Kim, I. F.; Tomashevsky, M.; Marshall, K. A.; Phillippy, K. H.; Sherman, P. M.; Holko, M.; Yefanov, A.; Lee, H.; Zhang, N.; Robertson, C. L.; Serova, N.; Davis, S. and Soboleva, A. (2013) NCBI GEO: Archive for functional genomics data sets - Update, *Nucleic Acids Res.*, **41** PMID:21097893
12. Dezső, Z.; Nikolsky, Y.; Sviridov, E.; Shi, W.; Serebriyskaya, T.; Dosymbekov, D.; Bugrim, A.; Rakhmatulin, E.; Brennan, R. J.; Guryanov, A.; Li, K.; Blake, J.; Samaha, R. R. and Nikolskaya, T. (2008) A comprehensive functional analysis of tissue specificity of human gene expression,

BMC Biol., **6**, 49. PMID:19014478

13. Brandvain, Y. and Wade, M. J. (2009) The Functional Transfer of Genes From the Mitochondria to the Nucleus: The Effects of Selection, Mutation, Population Size and Rate of Self-Fertilization, *Genetics*, **182**, 1129–1139. PMID:19448273
14. Berg, O. G. and Kurland, C. G. (2000) Why Mitochondrial Genes are Most Often Found in Nuclei, *Mol. Biol. Evol.*, **17**, 951–961. PMID:10833202
15. Anderson, S.; Bankier, A. T.; Barrell, B. G.; de Bruijn, M. H. L.; Coulson, A. R.; Drouin, J.; Eperon, I. C.; Nierlich, D. P.; Roe, B. A.; Sanger, F.; Schreier, P. H.; Smith, A. J. H.; Staden, R. and Young, I. G. (1981) Sequence and organization of the human mitochondrial genome, *Nature*, **290**, 457–465. PMID:7219534
16. Satoh, M. (1991) Organization of multiple nucleoids and DNA molecules in mitochondria of a human cell, *Exp. Cell Res.*, **196**, 137–140. PMID:1715276
17. Hu, X.; Lu, Z.; Yu, S.; Reilly, J.; Liu, F.; Jia, D.; Qin, Y.; Han, S.; Liu, X.; Qu, Z.; Lv, Y.; Li, J.; Huang, Y.; Jiang, T.; Jia, H.; Wang, Q.; Liu, J.; Shu, X.; Tang, Z. and Liu, M. (2019) *CERKL* regulates autophagy via the NAD-dependent deacetylase *SIRT1*, *Autophagy*, **15**, 453–465. PMID:30205735
18. Lázaro-Guevara, J. M.; Flores-Robles, B. J.; Garrido, K.; Pinillos-Aransay, V.; Elena-Ibáñez, A.; Merino-Meléndez, L.; López-Martínez, J. A. and Victoriano-Lacalle, R. (2018) Gene's hubs in retinal diseases: A retinal disease network, *Heliyon*, **4**, e00867. PMID:30417144
19. Krzywinski, M.; Schein, J.; Birol, I.; Connors, J.; Gascoyne, R.; Horsman, D.; Jones, S. J. and Marra, M. A. (2009) Circos: An information aesthetic for comparative genomics, *Genome Res.*, **19**, 1639–1645. PMID:19541911
20. Newman MEJ, Barabási A-L, Watts DJ. The structure and dynamics of networks. Princeton University Press, 2006.

FIGURES

Figure 1.- Data integration workflow to build the `RPGeNet` core database.

The final graphical web interface (bottom panels) depends on a series of data integration steps that provide the interactions and nodes to the main `neo4j` database engine. Each component of the workflow is described in detail on the main text.

Figure 2.- Visual representation of the expansion of the core interactions graph.

The graph builder begins with the construction of the skeleton graph, represented by all the nodes on the leftmost panel. The skeleton is created by finding the shortest direct interaction paths (the red arrows) between all the known driver genes—drawn here using the same shapes as in `RPGeNet Network Explorer` (star, square, and diamond shapes, based on whether their mutations cause or not syndromic diseases)—. The graph is then expanded into level 1 (represented as the orange panel) by adding all of the parents and children (straight lines) of the nodes already found in the skeleton graph. Level-specific interactions are shown as curved connections linking nodes within a given graph level. The expansion is repeated until the highest level is reached and all known genes with known interactions have been connected to the interactions core graph. The remaining genes that do not have any known interaction that connects them with the core graph are included in the whole graph level. Some of those genes may have interactions with other genes found only in the whole graph level (and many of those interactions are self-references). The bottom table from this figure compares the number of nodes and edges added on each level ("New Nodes" and "New Edges" rows), as well as the total number of nodes and edges accumulated.

Figure 3.- The renewed `Network Explorer` interface of `RPGeNet`.

In this example, the `Network Explorer` interface shows a subgraph containing all the genes that directly interact with *CERKL* within the skeleton subnetwork, after expanding nodes for *PPM1A, VHL,* and *MICAL3* (those selected four nodes highlighted in green, driver genes border in purple, node colors based on the "ABSOLUTE" gene-expression data). Some improvements to the interface can be appreciated: a "search" gene add-on at the mid-bottom, an expression data set selection drop-down menu at right-top corner of the network visualization canvas, as well as the "`undo`"/"`redo`" buttons. A more dynamic "buttons panel" on the right facilitates the interaction with the network data. Finally, the coloured-by-type interactions also provide directionality information with arrow heads, and have reliability-score proportional widths adjusted to the number of evidences supporting them. This figure can be reproduced on the Network Explorer if users upload the Supplementary File 1.

Figure 4.- Example of a pathways list returned using the shortest pathways `RPGeNet` query.

CERKL and *CSPP1* where used to start the pathway search on the main `RPGeNet` form. Only the first three pathways of the 28 retrieved by that query are shown on this figure, all of them at the shortest path length of three (3 edges and 2 nodes between the chosen identifiers). By clicking on the corresponding "Explore Network" button on any of the listed pathways, users can easily jump to the Network Explorer interface to work on the selected genes for that pathway.

Figure 5.- An example of node (left) and interaction (right) information panels from the `Network Explorer` interface.

The default behaviour "*On click*" of the `Network Explorer` interface is to show "*node properties*" (see topmost controls on the right panel of that interface on previous figure). From the network example of Figure 3, when clicking at the *CERKL* node the gene/protein information panel pops up to display a description of the gene, known aliases, a summary of its expression levels and functional annotation, and links to external references. On the other hand, by clicking on an edge, *VHL* to *CERKL* in this example, the interaction panel pops up, providing information about the type of possible interactions (genetic [blue], physical [red], or "unknown" [black]), as well as a series of tables containing details about the supporting evidences from the distinct sources, along with the corresponding external links to the reference databases and to the supporting evidences when possible.

Figure 6.- Analysis of `RPGeNet` v1 and v2 networks connectivity with `Circos` (19).

The figure compares the connectivity of the driver genes of the old `RPGeNet` v1.0 (left) and the updated `RPGeNet` v2.0 (right). The top pair of plots show all the shortest paths between driver genes at distance one, meaning direct interaction between each pair of driver genes. The bottom par of plots provide the comparison at distance 3, meaning there are two genes in the shortest path between a pair of driver genes of interest. It is clear from the visualized `Circos` plots that the updated `RPGeNet` database has a highly connected network.

Figure 7.- A control case interaction visualized on `RPGeNet` showing the connections between *NRL*, *NR2E3* and *CRX* retinal transcription factors.

A) `RPGeNet` was queried to display the subnetwork among *NRL*, *NR2E3* for the nodes at distance one at level 1; then nodes connected to *CRX* were added with the "*Node Addition*" button activated on click. *CRX*, *NRL*, and *NR2E3* are three well-known transcription factors that co-regulate retinal-specific genes, among them *RHO*. Interestingly, several other genes that cause retinal dystrophies also appear in the network as target genes or other transcriptional regulators (border shown in purple). Nodes color-fill defined by the "RET-ALL" gene-expression data.

B) Further trimming of the subnetwork obtained by omitting the nodes that are not chromatin remodelers or transcription factors provide an overview of relevant co-regulators of retinal genes. After deleting the corresponding nodes, further edges—out of the shortest paths that link the genes left—were shown by clicking on the "*Connect Genes*" button at the control panel. This visualization allows pinpointing and exploring alternate pathways that connect the initial seeds; for instance, *CRX* and *NRL* were already connected, but longer paths are now evident like *CRX⇌PRKN⇌HNRNPK⇌NRL*, when *PRKN* and *HNRNPK* are linked. Another example can be the pathway found between *NRL* and *NR2E3* on the path *NRL⇌SMAD4⇌PIAS3⇌NR2E3*, when *SMAD4* and *PIAS3* are linked. Both panels from this figure can be reproduced on the Network Explorer if users upload the Supplementary Files 2 and 3, respectively.

SUPPLEMENTARY MATERIAL

Supplementary Figure 1.- Bar plot with distribution of the different evidences used by `STRING`.

`STRING` is a protein-to-protein interactions database that includes predictions and experimentally validated interactions. The experimentally validated interactions include evidences from different sources. The proportion of such evidences is shown in this bar-plot. `GRID`, `INTACT`, `KEGG`, `BIOCARTA` and `REACTOME` are the most common sources of experimentally-validated interactions from `STRING`.

Supplementary Figure 2.- Choosing the optimal votes cut-off for `PPaxe`.

The graph shows the distribution of the un-normalized confidence score given to the interactions detected by `PPaxe` with respect to the number of `PPaxe` interactions (counts), and the average score depending on the cut-off score chosen (vertical line). In an attempt to optimize the number interactions while reducing false positive interactions and increasing false-negatives, the cut-off score chosen was 0.65, which was the value that yielded a minimum precision of 90% in the validation assessment performed in the original PPaxe manuscript (6). “e” and “n” correspond to the number of edges and nodes respectively that are passing the cut-off threshold. Panels categorize by the percentage of votes by the random-forest classifier.

Supplementary Figure 3.- Comparing the number of counts between in and out degrees for each level of the RPGeNet core network.

In the skeleton graph the much smaller area between the in-/out-degree lines in comparison to higher level graphs denotes a smaller number of interactions. Comparing the skeleton with level one, there is a large increase in counts for lower degrees. However, as the graph is expanded to levels two and three, a decrease of counts in lower degrees can be observed. This can be explained by a larger jump in total number of interactions between skeleton and level one, followed by smaller increments in new interactions and a slower increase in degree from levels one to three, as new nodes and interactions are introduced.

Supplementary Table 1.- Topology of the `RPGeNet` interactions graph.

This table shows the total number of nodes (genes/proteins, where adjacent nodes can be calculated by substracting *isolated* from *total nodes*) and edges (interactions, including counts by the type of relation between pairs of adjacent nodes: mutual-, asymmetric- and self-interactions), for every level of the `RPGeNet` database graph (see “`RPGeNet v2` All Sets” block). For comparison purposes, graph stats are provided for the previous `RPGeNet v1` version, as well as those for the interaction networks that can be produced separately from each of the evidence sources (blocks named as “`BioGRID` Only”, “`STRING` Only”, and “`PPaxe` Only”, respectively). Most of the *isolated nodes* correspond to drivers without interaction evidences from the selected sources, and those numbers complement the values from the *adjacent drivers* row. Graph statistics were described in depth on Newman *et al* (20). In brief: *graph density* is the ratio between edges and the total number of possible vertices (nodes); *average clustering coefficient* (or the tendency of nodes to cluster together) is a measure of how complete the neighborhood of a node is, over all the nodes of the network; *diameter* is the maximum distance between two nodes; *reciprocity* is the; *average degree* is the average number of edges per node; *closeness* is the average length of shortest path between a node and every other node in the network; *betweenness* is a measure of the number of times a node is found within the shortest paths between two other nodes; *coreness* defines the shell index of the vertices of a network; *eccentricity* is the maximum of the shortest distances of a node with

respect all other nodes in the graph; finally, *average path length* is a measure of the average distance between two nodes. Graph stats produced with `python-igraph` library (v0.7.0, see further details at https://igraph.org/python/).

Supplementary Table 2.- Source origin and redundancy of evidences for `RPGeNet` core network interactions.

First block accounts for the number of nodes and edges supported by each input source at each graph level of the core network. Second block provides information about distinct evidences used to weight the interactions. Number of interactions on the core network with `STRING` scores is provided on the last block to complement evidences (also shown in the interaction information pop-up cards like the one shown in Figure 5 right panel). `RPGeNet` models interactions as directed edges between nodes, and thus, represents undirected interactions between genes as two separate interactions. The "Total Interactions" column shows the total number of edges stored in the database (without taking into account the number of evidences), counting reciprocal interactions twice (A⇀B is different from B⇀A). The "Non-redundant Interactions" column refers to the number of interactions in `RPGeNet` independently of the direction, and thus, the pair A⇀B and B⇀A is only counted once. The Non-redundant interactions count criteria is equivalent to the one used by the `BioGRID` database (https://wiki.thebiogrid.org/doku.php/build_3.5.171).

Supplementary Table 3.- Interaction sources overlap against the `RPGeNet` skeleton and wholegraph networks.

To provide an estimate of the overlap for the evidences gathered from each source, this table shows the intersection of total number of nodes and edges for the skeleton and wholegraph networks produced when the `RPGeNet` pipeline is run over each of the interactions sources separately. First row has the totals for the whole network that is produced when combining all the sources when running the full pipeline to create the graph levels integrated on the `RPGeNet v2` database, already shown on the previous Supplementary Tables. See also Supplementary Table 4 for further details about edges defined from the analysis of each separate interaction sources.

Supplementary Table 4.- Edges classification for the standalone pipeline analyses over each separate interaction sources versus `RPGeNet` core network.

The "Total Interactions" column shows the total number of edges stored in the database (without taking into account the number of evidences), counting reciprocal interactions twice (A⇀B is different from B⇀A). The "Non-redundant Interactions" column refers to the number of interactions in `RPGeNet` independently of the direction, and thus, the pair A⇀B and B⇀A is only counted once. Further details on all graph levels for the `RPGeNet` core network are provided on Supplementary Table 2.

Supplementary Table 5.- Predicted interactions for unconnected drivers genes on `RPGeNet` core network.

Driver genes can be unconnected to the core interactions whole-network graph because there are no known experimentally validated interactions that link them to the rest of nodes from the whole-network graph. Number of predicted interactions and putative interactors were retrieved from `STRING` database. The

predicted interactions were not included into the whole core network on this release. Bottom row shows a driver gene, *C1QTNF5* (formerly identified as *CTPR5*), which is aggregated by the protocol to the growing graph at level 1 expansion yet it only has a validated interaction to itself from that point to the whole-graph. From seven unconnected driver genes of the `RPGeNet` previous version (2), five are still listed on this table—those marked with * —, while the updated interaction evidences made possible to connect the other two (*HGSNAT* and *PCARE*, the latter formerly identified as *C2ORF71*).

Supplementary Table 6.- Predicted interactions for "unconnected" non-driver genes on `RPGeNet` core network.

Non-driver genes can be unconnected to the core interactions whole-network graph because there are no known experimentally validated interactions that link them from the whole-graph network level to the rest of nodes at any level of the `RPGeNet` core network. Number of predicted interactions and putative interactors were retrieved from `STRING` database. The predicted interactions were not included into the whole core network on this release. The last three genes have self-interactions, while the others are connected only among them in pairs at the whole-graph level (as for instance, *DEFB106A* and *DEFB106B*).

Supplementary File 1.- "`SFile1_RPGeNet_Upgrade_2019_Database_Figure3.json`"

`JavaScript JSON` file containing the graph data for the nodes and edges found by the query described on Figure 3, as well as the coordinates to place the nodes with the same layout as shown on this figure. To reproduce such figure, one can upload this file by clicking on the "*Upload*" button of the controls panel in the Network Explorer window, or from the "*Network Explorer*" web form section at the `RPGeNet` home page. If no gene identifier is provided when clicking on the "*Explore Network*" button from that form page, a pop up panel will appear requesting to upload a graph file, such the one provided.

Supplementary File 2.- "`SFile2_RPGeNet_Upgrade_2019_Database_Figure7A.json`"

`JavaScript JSON` file containing the graph data for the nodes and edges found by the query described on Figure 7A, as well as the coordinates to place the nodes with the same layout as shown on this figure. To reproduce such figure, one can upload this file by clicking on the "*Upload*" button of the controls panel in the Network Explorer window, or from the "*Network Explorer*" web form section at the `RPGeNet` home page. If no gene identifier is provided when clicking on the "*Explore Network*" button from that form page, a pop up panel will appear requesting to upload a graph file, such the one provided.

Supplementary File 3.- "`SFile3_RPGeNet_Upgrade_2019_Database_Figure7B.json`"

`JavaScript JSON` file containing the graph data for the nodes and edges found by the query described on Figure 7B, as well as the coordinates to place the nodes with the same layout as shown on this figure. To reproduce such figure, one can upload this file by clicking on the "*Upload*" button of the controls panel in the Network Explorer window, or from the "*Network Explorer*" web form section at the `RPGeNet` home page. If no gene identifier is provided when clicking on the "*Explore Network*" button from that form page, a pop up panel will appear requesting to upload a graph file, such the one provided.

RESPONSE TO REFEREES for Manuscript ID DATABASE-2019-0032.R1

General Comments:

When dealing with this second reviewing stage, we have detected two bugs: one in the `RPGeNet` building protocol and another one in the web interface. First one was due to an error dealing with `BioGrid` aliases that duplicated some nodes (one having the official symbol and another with the synonim appearing in the `BioGrid` record). We have fixed the unaliasing functions to solve this bug, which reduced the number of nodes on the wholegraph to 18,542. It diminished also the number of interactions to 1,201,066, when merging the evidences supporting the duplicated node interactions to the corresponding official node. The second bug was on the query to the database that is made to show the interactions available at a given graph level between each pair of selected nodes. It can happen that one interaction A⇀B appears in one level, like in the shortest paths computed to build the skeleton, and the reciprocal B⇀A is recovered from the next level expansion of parent/child interactions, level 1 here; however, to simplify the visualization and navigation at each level, the *Network explorer* should display only the interactions that are defined up to the initial query level (skeleton, level 1, etc..., see **Fig. R1** below that illustrates this). For this bug, starting a search over the skeleton graph from home page was showing the interactions at skeleton level but in some cases also those at level 1. After both bugs fixed, we had to update the screenshots for figures 3, 4, and 7 (former Figure 6, but we have added a new figure to show the new implementation of the gene and interaction pop-up information cards).

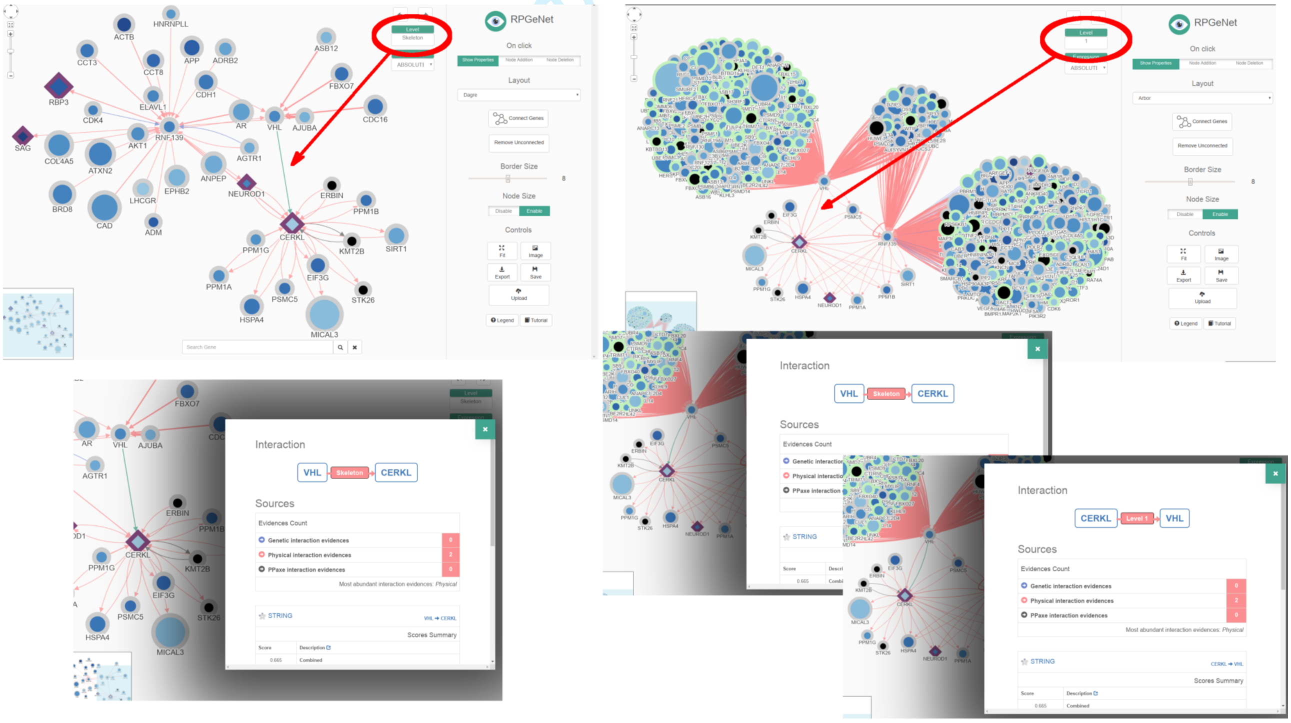

Fig. R1: Same identifiers were used for the initial query *CERKL*, *VHL*, and *RNF139*. Left panels show the resulting graph when selecting "*Skeleton*" level, whilst right panels display the outcome when choosing "*Level 1*" level on the *Network Explorer* form section at the `RPGeNet` main page. One can see on the right level one query example that two edges were retrieved between *CERKL* and *VHL*: first edge *VHL*⇀*CERKL* from the skeleton graph, as in the left example query, and the reciprocal edge *CERKL*⇀*VHL* that appears at level 1 (as it was not used in a shortest path to build the skeleton graph level for the core network). It is worth to mention here the improved interaction pop-up information cards that can be appreciated on the three bottom images from this figure.

Reviewers 2 and 3:

Reviewer 3 Comments to the Author*: I find the content of this manuscript merits publication. The comments/answers from the authors satisfactorily explain what Ii felt needed clarification. (I assume inconsistencies in the language will be addressed by journal editors).*

Reviewer 2 Comments to the Author*: After reading the revised version of the manuscript, I consider the authors have made a substantial effort to answer the several issues I raised in the first version of the article. In particular, I'd like to mention:*

- ***A static link to previous release of the database.***
- ***Side by side comparison of data from Figure2 in the old and the new releases.***
- ***Minor corrections on the order of links, total numbers and references were solved.***
- ***New discussion on the general purpose usage of this tool and another reference published in the field.***

Therefore, I think the manuscript now in its current form is ready to be accepted for publication in the Database journal.

We are grateful with the comments and suggestions requested by those two referees in the previous revision stages, both considering now the manuscript ready for publication. We also want to specially thank Reviewer 2, who has further detailed those improvements made to the manuscript he/she considered worth to mention.

For Peer Review

Reviewer: 1

Comments to the Author
One of my main criticisms on the original manuscript was that it was not clear how bidirectional edges were created. It is now much clearer in the manuscript and the rebuttal how these are created. Unfortunately, this also means that it is clearer that `RPGeNet` has a large problem with redundant edge information, as detailed below.

We would like to thank again referee 1 for his/her insightful comments and suggestions, yet we strongly disagree with the thesis that `RPGeNet` has a large problem of redundant edge information. We will try to address this misconception in the following sections below, specifically in the answers to 1, 2, 3 and 4. We also consider that we have tried to clearly describe the specific features that make `RPGeNet` worth publishing on our previous reply; we assume that we were able to answer appropriately to the other questions posed by referee 1 on the previous reviewing round too. Hereinafter, we will try to do our best to shed light on those aspects over the following answers and to provide the corresponding solutions and fixes on the main manuscript.

---

1) Edges that are duplicated by artificially creating bidirectional edges to represent a single undirected edge seem to be counted towards the total number of edges in the database. These are artificial because no experiment showed that the interactions are bidirectional in two opposing directions.
Please provide a count of how many interactions the total database contains not counting bidirectional edges created from undirected edges. i.e. an undirected edge in an original source like `BioGRID` should count as 1. A directed edge from an original source should count as 1.

We would like to note that, in our opinion, representing an undirected interaction as two directed interactions is not necessarily artificial, as it is just a way to model an undirected graph in a directed graph representation. Given a protein-protein interaction where there is contact between the two proteins, it can either be modeled as a single undirected interaction (A—B) or as two reciprocal directed edges (A⇀B and B⇀A), connecting two nodes of the graph in any case. None of these approaches can be considered artificial, they are equivalent for the purposes of graph traversal and shortest path computation. We chose the directed graph approach because it simplifies many issues, specifically it helps when computing the shortest paths between genes, to initialize the core of our network implementation (the skeleton graph), and when storing the interactions in `Neo4j` in a way that allow us to easily compute the shortest paths of a query gene from the whole graph against the set of driver genes. This later feature now allows users to look for relations of novel putative causative genes against the set of known ones that define the core network.

Nevertheless, we understand that this information could be useful for researchers, and thus we have added it to the supplementary materials (Supplementary Table 2, see below an excerpt of the first block that summarizes contribution of each data source to unique nodes and edges at all the graph levels). We have also included this table to the "Data" page on the `RPGeNet` website (https://compgen.bio.ub.edu/RPGeNet/data).

GRAPH SUMMARY	Skeleton	Level1	Level2	Level3	WholeGraph
**Total UNIQUE NODES**	4 018	17 851	18 512	18 527	18 542
Adjacent Nodes	4 002	17 836	18 497	18 512	18 527
Isolated Nodes	16	15	15	15	15
**NODES by Source**					
BioGRID	3 677	14 831	15 132	15 136	15 139
STRING	3 580	12 864	13 253	13 263	13 269
PPaxe	1 407	3 016	3 054	3 056	3 062
TOTAL	8 664	30 711	31 439	31 455	31 470
Nodes source "redundancy"	215.63%	172.04%	169.83%	169.78%	169.72%
**Total DIRECTED EDGES**	35 528	932 340	1 217 902	1 218 017	1 218 032
Mutual [A⇌B]	9 601	462 988	604 652	604 707	604 713
Assymetric [A⇀B]	16 326	5 074	5 931	5 931	5 931
Self-loop [A⇀A]	0	1 290	2 667	2 672	2 675
Total Non-Redundant	25 927	469 352	613 250	613 310	613 319
Directed edges "redundancy"	137.03%	198.64%	198.60%	198.60%	198.60%
**EDGES by Source**					
BioGRID all	22 914	518 478	623 643	623 656	623 659
BioGRID only	21 334	483 667	579 207	579 220	579 220
STRING all	12 563	440 111	629 167	629 265	629 271
STRING only	10 599	402 920	582 094	582 190	582 190
PPaxe all	2 277	12 282	13 572	13 578	13 584
PPaxe only	1 534	8 049	8 984	8 988	8 988
TOTAL	37 754	970 871	1 266 382	1 266 499	1 266 514
Edges source "redundancy"	145.62%	206.85%	206.50%	206.50%	206.50%

2) Additionally, redundant edges from two sources that collect information from the same paper must not be counted twice. If a paper describes an interaction, it should only be counted once. For example, given the genes CERKL VHL, `RPGeNet` displays these as having two artificially constructed directional arrows connecting them. Clicking on each edge lists 6 physical interactions from `BioGRID` and `STRING`. Note that all of the interactions come from one paper (PMID: 26296657). Actually, if you look at this paper, it provides evidence for a single interaction, which `BioGRID` captures as being supported by 3 evidence sources (2 affinity capture and one reconstituted complex experiment types). The `STRING` interactions seem to just duplicate these (likely because `STRING` imports `BioGRID`). This should be counted as 1 interaction (one paper), supported by 3 experiments.

For Peer Review

We do not count an interaction between a pair of nodes that is available from two different sources as two interactions but as an interaction having two supporting sources or evidences. Our interactions counts are independent of the number of evidences, sources or experiments each interaction has (see next answer below, where we try to address this issue). We consider physical (red edges), genetic (blue edges), and `PPaxe` (black edges), separately, thus two nodes may have as much as three distinct connections (either directional or bi-directional depending on the supporting evidences of course). We decided to store them in that way as the split visualization provides users with extra information on the network explorer about the kind of interactions described between nodes. However, the interaction panel that pops up when user access to an edge properties, shows the summary of all evidences available between the two nodes (two physical evidences in this example, both from `STRING`). To avoid confusion between evidences and edges, we have improved the information card design (as it can be also appreciated on **Fig. R1**), and reworded the sections on the interaction card, as it can be appreciated on the side **Fig. R2**.

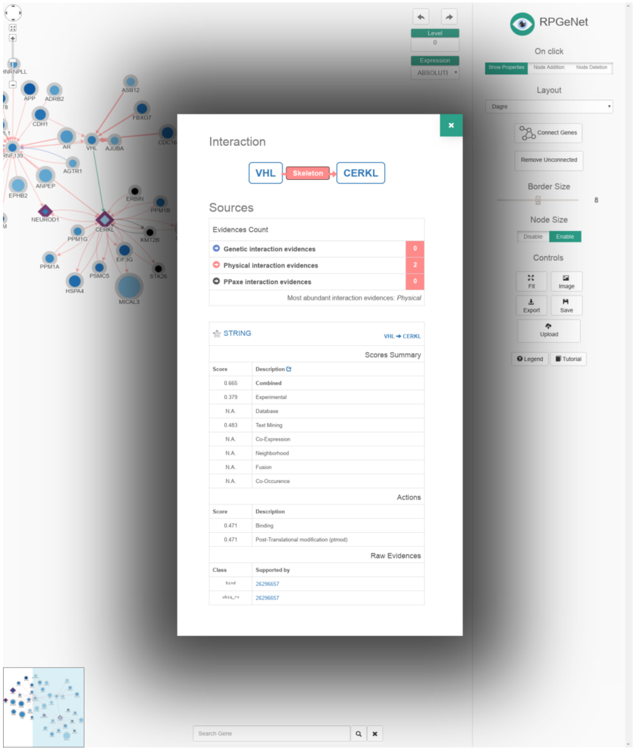

Fig. R2: An example of the renewed interaction information card pop-up panel. *CERKL*, *VHL*, and *RNF139* identifiers were used for the initial query over the skeleton graph level. Most supporting evidences are physical so the card is of "physical" type, shown as red in some the panel features (interaction image and evidences summary table).

In the same network example from that figure, we can find *AGTR1* and *RNF139* nodes connected by a pair of edges at skeleton level, both a physical and a genetic directed one. **Fig. R3** on the right shows the interaction panel appearing when user clicks on any of those two edges, which displays all the supporting evidences between those two nodes; it has also tagged the interaction as "*mixed*" (highlighted in yellow) because there is no evidence type summing up more than the others. The interaction *AGTR1*⇀*RNF139* is supported by two evidences, a genetic and a physical one, both from BioGRID.

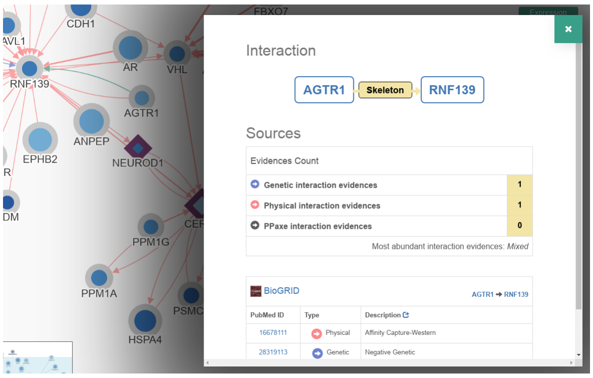

Fig. R3: An example of an interaction, between *AGTR1* and *RNF139*, supported by same amount of different evidences ("mixed" type, shown as yellow on some of the panel features).

Essentially, the interaction A⇀B is only counted once. If the interaction is undirected, A⇀B is counted once, and B⇀A is counted once. Neither the source nor the number of evidences or experiments has any impact on these counts, which are the ones listed in the manuscript.

On the other hand, it is true that we were showing just the evidence code supporting each interaction and no other related information. We agree that this may lead to confusion as the specific evidence source was not provided. Thus, as for the interaction information example commented by

the referee, for the *VHL⇌CERKL* case, one could only retrieve the `PubMed` ID without the appropriate context. We have improved those tables pairing each code with its related evidence description from the corresponding database. We have also fixed a bug where the total evidence counts for physical interactions from `STRING` was counting twice the `PubMed` evidences when other database evidences were empty (see **Fig. R4** below). When one of such categories are empty, they are also not shown on the interaction information panel (as it can be appreciated on the right bottom figure, where there is no empty "*Evidences*" subsection).

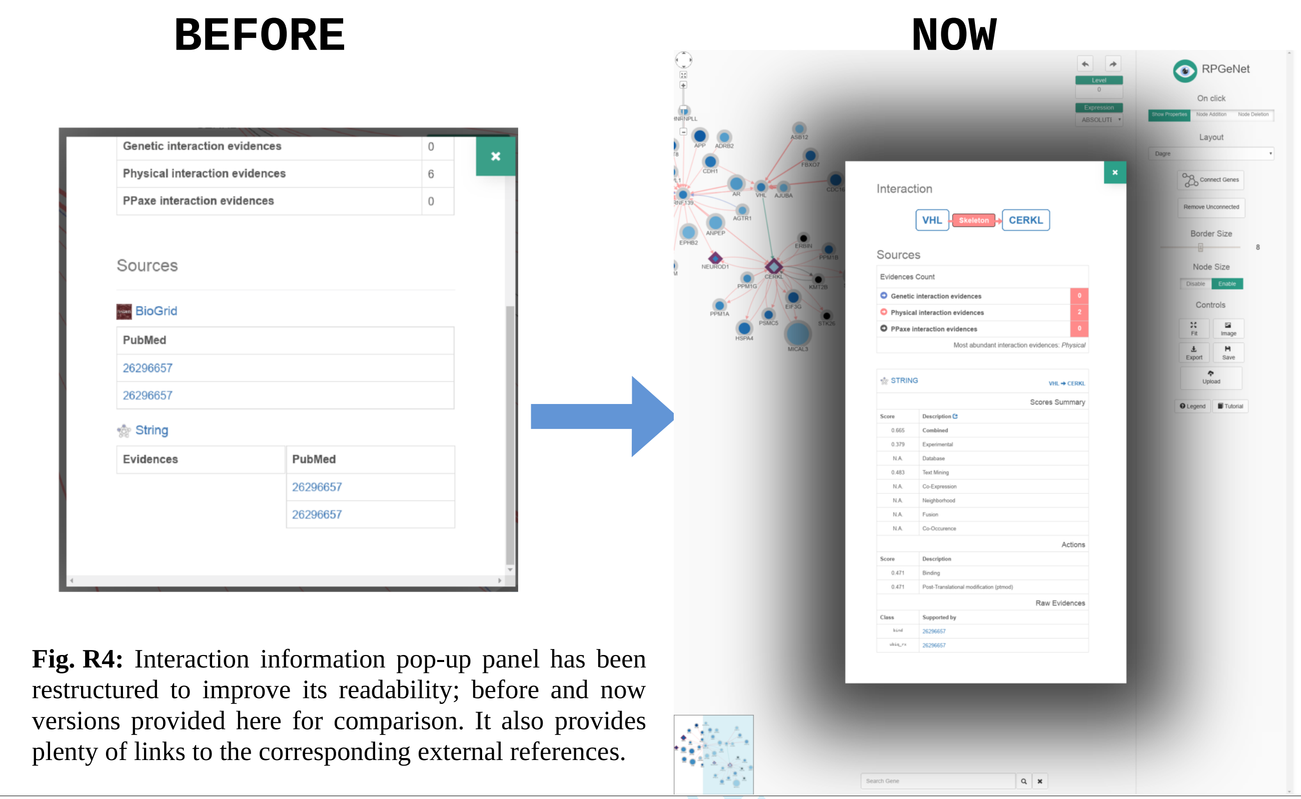

Fig. R4: Interaction information pop-up panel has been restructured to improve its readability; before and now versions provided here for comparison. It also provides plenty of links to the corresponding external references.

3) Presumably, due to the artificial creation of bidirectional edges and the duplication of interactions from multiple sources, this is currently counted as 12 interactions (6 from each bidirectional edge). As you can see, this likely leads to massive overcounting of interactions and the numbers reported in the paper are most likely highly redundant.

As we thoroughly described in the previous point, we do not count that interaction as 12 interactions. We currently count it as <u>two</u>: one for each direction, because we represent undirected interactions as two directed interactions/edges. The evidences (2 in this case, instead of 6, after the bug fix already described in the previous point and the one from db queries to limit interactions shown at a given level described in the general comments), shown in the information panel when clicking on either edge (see Figs. R1, R2, R3, R4), are stored independently as evidences, and thus they are not counted in the reported interaction counts in the manuscript nor in the supplementary materials. Although it is true that we count reciprocal interactions twice for the total number of interactions, and that we expand the undirected interactions into two directed edges, the statement "this likely leads to a massive overcounting of interactions" is false. We do not count each evidence or source as a different interaction anywhere in the main manuscript, the supplementary materials, nor the data page of the web-application. We have included in "Supplementary Table 1" a fully detailed characterization of nodes and edge types, at each graph levels of the `RPGeNet` core network, taking into account total nodes and isolated ones (number of adjacent nodes can be

calculated easily from those two values), as well as total directed, non-redundant, mutual, assymetric and self-loop edges.

In “Supplementary Table 2” those values can be compared with the counts of supporting evidences by Class and Sources. To further illustrate this, for interactions stored in `RPGeNet`, reciprocal interactions retrieved from `BioGRID` are counted only once, summing up a total 316,179 non-redundant interactions (see “Supplemen-tary Table 4”, where the individual contribution of each source database is provided in terms of total numbers within each type of edge after classifying them in mutual, assymetric, self, directed, and non-redundant). This is comparable to the non-redundant counts listed in the `BioGRID` stats page (373,866; look for *Homo sapiens rows at*: https://wiki.thebiogrid.org/doku.php/build_3.5.171).

The differences between these two counts can likely be attributed to the gene symbol disambiguation that is performed in the RPGeNet pipeline before merging the interactions from the `BioGRID`, `STRING`, and `PPaxe` data sources, which in the end results <u>in less</u> non-redundant interactions stored in `RPGeNet`, <u>not more</u>.

In order to clear the confusion between “evidences” and “interactions”, we have updated the text in the information panel that appears on the web application when clicking on an edge (see **Fig. R5**, and examples from other figures included in this documen, as the “before/now” comparison illustrated on the previous page in **Fig. R4**).

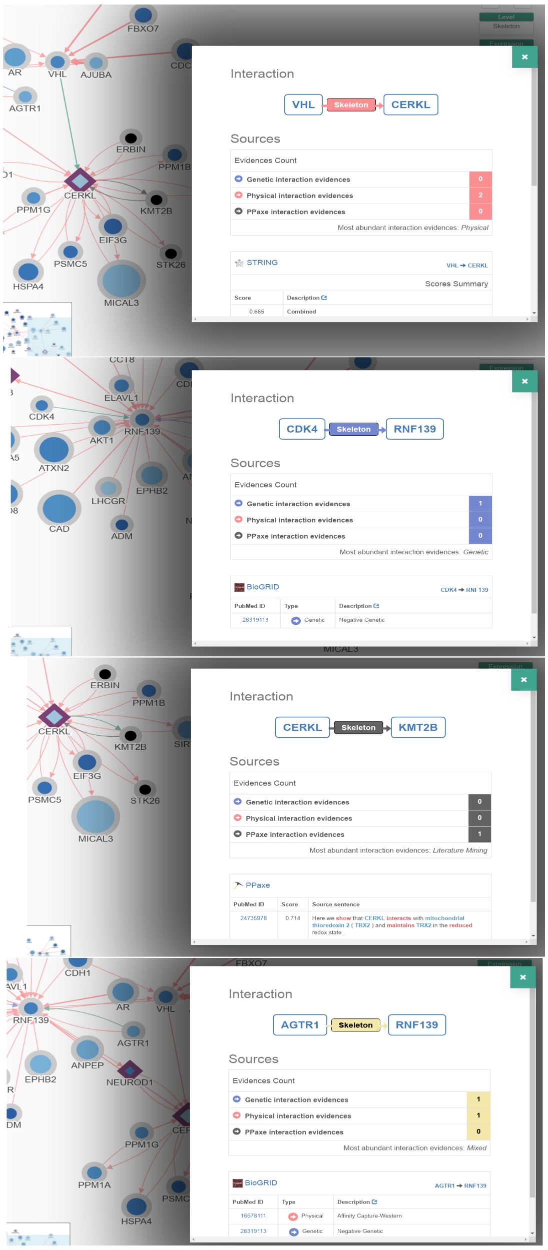

Fig. R5: To improve interpretation of the interaction cards different colors have been associated to the type of the most abundant supporting evidence: physical (red), genetic (blue), unknown (black), and mixed (yellow).

4) This is why I questioned the high numbers of interactions reported in the manuscript. Now the number is even bigger at 1,210,705. Given that `BioGRID` has 378,455 non-redundant interactions for all of human, it is highly unlikely that a specialized database like `RPGeNet`, which aims to create a smaller, more user friendly subset of interactions compared to `BioGRID` and `STRING` would have many times the number of interactions as the entire `BioGRID` database. This is a major problem with RPGeNet – I can’t see how it will be useful for users to have such inflated numbers of interactions.

We tried to address this concern in the answers to the previous revision stage (more specifically on *question 3b* of that document). The large size of *WholeGraph*—now 1,218,032 total directed interactions, or 613,319 non-redundant ones (see Supplementary Table 4)—does not have any impact on the usefulness of `RPGeNet`. The size of *Skeleton* graph (35,528 directed interactions, or 25,927 non-redundant ones) is the one that should be used to assess if `RPGeNet` is useful as a specialized database. In the previous `RPGeNet` version, the whole graph was there just in case the

For Peer Re

user was expanding the *Skeleton* or any of the other sub-graph levels, by clicking on the "N*ode addition*" button to explore "side effects". Our new implemented engine facilitates a more powerful traversal of the complementary information stored in the *WholeGraph*, in order to explore for instance the relationships of novel retinal dystrophies candidate genes with the core *Skeleton* (such functionality provided on the main form as "*Pathway to Level*").

Again, `RPGeNet` hosts 1,218,032 interactions on its core network (613,319 non-redundant). This graph, called in our manuscript *WholeGraph*, corresponds to the whole known interaction network of genes and proteins in *Homo sapiens*, gathered from distinct sources. It corresponds to the whole human network resulting from the merger of `BioGRID`, `STRING`, and `PPaxe`. Although `STRING` imports `BioGRID`, as properly stated by the referee in the preceding question 2 of the current document, we already knew that the intersection is not fully inclusive as it is described on the comparison of skeletons derived from each database in the supplementary figure S2 of Boloc *et al*, 2015 (https://doi.org/10.1371/journal.pone.0135307.s002), as well as in the Supplementary Table 3.

Even if *WholeGraph* is stored in `RPGeNet`, researchers interested in the interactions related to retinal diseases are expected and encouraged (by the text in the manuscript, the tutorial page, and the tutorial video) to start searches in the ***Skeleton* graph**, which only holds **35,528 interactions** (25,927 non-redundant ones). **This is the network that is enriched with genes and interactions related to retinal diseases, and it is the one that would correspond to the "*smaller, more user friendly subset of interactions*" that Reviewer 1 states in the question.** Please, note that this network only contains 2.9% of the total interactions from *WholeGraph*.

The usefulness of `RPGeNet` stems from the separation of the whole network in different subgraphs, allowing researchers to explore a relatively small graph (the *Skeleton* graph) to pinpoint possible disease candidates and to analyze relations that can define novel pathways among them. Additionally, `RPGeNet` provides ways to explore the whole network as a side feature in relation to that *Skeleton*. As an example, the new implementation that can deal with the whole network allowed us to create the new "*Pathway to Level*" functionality, which tries to find shortests paths to driver genes, and their direct interactors, for query genes that are not located in the *Skeleton* graph.

5) This must lead to usability problem. For example, I tried expanding the network by clicking on a few nodes using the "Node Addition" mode, and the network got so big, so quickly that the webpage crashed my Chrome browser.

As stated in the previous answer, users are expected to mainly work at the *Skeleton* level, which only holds 35,528 interactions. As the implementation of the network navigator runs on the client-side, the real final performance may vary depending on the hardware capabilities where the end user runs a web-browser to access `RPGeNet`. One can see also a large network already shown for *Level 1* subgraph on the right-side screenshots from **Fig. R1**. However, here we provide a link with a real-time video showing how `RPGeNet` interface can handle many "Node Addition" clicks and how it can also deal with layout changes in reasonable time for large sets of nodes:

`https://youtu.be/BN5A5onwgFw`

Additionally, on the meantime we have applied many of the performance recommendations listed in the `cytoscape.js` website (`http://js.cytoscape.org/#performance`). Unfortunately, we can't improve the performance further, and still it will depend at last on the user's machine capabilities as we just said.

For Peer Review

6) "For* `BioGRID` *all genetic interactions are assumed to be unidirectional" – There are multiple types of genetic interactions in biogrid and many of them are not directed. For instance, positive or negative genetic interaction types are not directed.

Initially we assumed the directionality already defined in the database. In order to address previous concerns of Reviewer 1 (<u>question **2a**</u>, which stated: *How can the interactions be directed if they include undirected PPIs from e.g.* `BioGRID`*?*), we considered physical interactions (PPIs) as bidirectional, and introduced that requisite on the analysis pipeline. For genetic interactions, where directionality may have an implied meaning that we want to preserve in our database, we followed `BioGRID` curation guide which states:

> ***If I have to arbitrarily choose a directionality for an interaction should I enter the interaction twice (once in each direction)?***
>
> *No, we curate using a spoke model and entering interactions in both directions will artificially inflate the number of interactions in the database (making it appear as if an interaction has been shown twice in a paper when it has not). Choose the directionality that makes the most sense based on* `BioGRID` *guidelines in the Directionality of Interactions (Baits/Hits) section and only enter the interaction once.*
>
> *Source:* https://wiki.thebiogrid.org/doku.php/curation_guide

7a) I previously asked what evidence sources were imported from* `STRING`*.

r Peer Review

We tried to answer to this concern in the previous revision (<u>questions **3a**</u> and <u>**3b**</u>), yet we haven't provided the numbers then. We will try to address both questions again here, as in the answer provided to question 4 of the current revision round.

> ***3a****) What evidence sources were used from* `STRING`*?*
>
> ***3b****) Even though it is less than before, 739122 interactions is still very large number of interactions – it seems like way too many to be useful for a specialized database. More detail about the* `STRING` *evidence sources and how the* `STRING` *data are filtered should be provided.* Maybe some of the evidence sources from `STRING` should be studied in more detail and filtered at a higher stringency level. *(…).*

EVIDENCE SUMMARY	Skeleton	Level1	Level2	Level3	WholeGraph
**TOTAL EVIDENCES**	75 447	2 371 305	3 209 677	3 209 856	3 209 871
**By Class**					
Genetic evidences	257	6 018	7 062	7 063	7 063
Avg. evids x directed edge	0.007	0.006	0.006	0.006	0.006
Physical evidences	70 561	2 342 154	3 177 567	3 177 739	3 177 748
Avg. evids x directed edge	1.986	2.512	2.609	2.609	2.609
Unknown evids (PPaxe)	4 629	23 133	25 048	25 054	25 060
Avg. evids x directed edge	0.130	0.025	0.021	0.021	0.021
**By Source**					
BioGRID	31 726	705 485	842 798	842 815	842 818
Physical interactions	31 469	699 467	835 736	835 752	835 755
Genetic Interactions	257	6 018	7 062	7 063	7 063
Avg. evids x directed edge	0.893	0.757	0.692	0.692	0.692
STRING	39 092	1 642 687	2 341 831	2 341 987	2 341 993
Avg. evids x directed edge	1.100	1.762	1.923	1.923	1.923
PPaxe	4 629	23 133	25 048	25 054	25 060
Avg. evids x directed edge	0.130	0.025	0.021	0.021	0.021

On the previous table, part the "Evidence Summary" block of Supplementary Table 2, we provide a breakdown of all the evidence sources imported into `RPGeNet` (including those from `STRING`). In the "Skeleton" column, one can see 39,092 evidences corresponding to 12,563 edges that are supported by `STRING` on the *Skeleton graph* (12,563 of the 35,528 total edges of course). Similar ratios can be observed at the different graph levels (and with respect to the other evidence sources). It is worth to pinpoint that there are on average around 2 to 2.7 evidences supporting unique edges at the different graph levels.

We have also performed an analysis of the separate contribution made by each source dataset, in terms of separate analysis, contribution to nodes and edges; such analysis has been distributed into two new Supplementary Tables: Suppl.Table 3 for the analysis of overlaps between the graphs produced when each source is taken separately from the others; and Suppl.Table 4, detailing the different types of edges obtained from each source, either separately or combined into the `RPGeNet` core network.

7b) Please see the `STRING` documentation at http://version10.string-db.org/help/getting_started/#evidence.

Evidence sources for `STRING` are:

Conserved Neighborhood, Co-occurrence, Fusion, Co-expression, Experiments, Databases, and Text mining.

For Peer Review

Those "Evidence sources" can be understood as categories to integrate all raw evidences recorded by interaction and to access them through their web interface. We have been always loading the raw evidences for the interactions filtered from the tables downloaded from the publicly available `STRING` database files (http://string-db.org/cgi/download.pl). We do not pretend to clone all the `STRING` (or `BioGRID`, etc) interface functionality; just to provide some additional information to users related to the interactions that were integrated into the `RPGeNet` graph.

The following table, an excerpt from Supplementary Table 2, provides the number of `RPGeNet` graph edges having a `STRING` evidence annotated into each of the categories at the distinct graph levels (take care that those values are not additive as one edge may have scores for one, some, or all categories).

EDGES with STRING score	Skeleton	Level1	Level2	Level3	WholeGraph
With any STRING score	12 563	440 111	629 167	629 265	629 271
With "experimental" score	2 696	75 075	109 191	109 231	109 235
With "database" score	9 112	371 666	541 032	541 082	541 084
With "text-mining" score	8 803	248 133	345 610	345 682	345 686
With "co-expression" score	2 227	107 246	157 974	158 008	158 010
With "neighborhood" score	0	0	0	0	0
With gene-"fusion" score	16	1 136	2 384	2 384	2 384
With "co-occurence" score	124	6 320	8 749	8 755	8 757

We want to reiterate (which was already stated in the previous answer 3b, and in answer 4 of this revision), that, in our opinion, the *Skeleton graph* is small enough to be useful for researchers (35,528 directed interactions, or 25,927 non-redundant ones), and we do not consider that `RPGeNet` needs any further filtering of interactions by `STRING` evidence sources.

To recapitulate the points discussed on this response to the referees, we state the following:

- `RPGeNet` does not aim to create a smaller database by sub-setting `BioGRID` or `STRING`.
- `RPGeNet` computes several subnetworks from the merger of `BioGRID`, `STRING`, and `PPaxe`. These subnetworks are nested and smaller than the whole network, with the smallest one containing only around 2.9% of the interactions of the whole network: 35,528 directed edges/interactions (25,927 non-redundant), filtered from 1,218,032 total directed edges/interactions (613,319 non-redundant).
- `RPGeNet` stores information about the evidences supporting the interactions integrated in all core network graph levels. Also important is that a single interaction, an edge in the graph, is made up from one or more evidences, and those are not accounted as individual edges (1,218,032 total directed edges/interactions derived from 3,209,871 total evidences gathered from three distinct sources).
- Users are expected to start searches in the smallest subnetwork, the *Skeleton graph*, when looking for candidate genes and pathways, which contains a short number of interactions (35,528 directed interactions, or 25,927 non-redundant ones).
- Having a large number of interactions in the *WholeGraph* level (1,218,032 interactions, 613,319 non-redundant) is not a problem, because `RPGeNet` protocol separates the network in different subgraph levels. Users are expected to look in the smallest one first (35,528 directed interactions, or 25,927 non-redundant ones).
- Keeping all the initial interactions resulting from the merger—the so called *WholeGraph, with* 1,218,032 total directed interactions (613,319 non-redundant)—, and classifying nodes and edges by nested graph levels, allows `RPGeNet` to:
    - Provide the “*Pathway to Level*” functionality, that tries to link new possible candidate genes to the current characterized driver genes and their interactors within the *Skeleton* graph level.
    - Give researchers the ability to explore the merger of the three data sources in the context of our application, that is, retaining the ability to map expression values, get the same information when clicking on genes, etc.
    - Distribute a dynamic and responsive visualization platform for the retinal dystrophies network, where we can integrate further information on the nodes, related for instance to future gene-expression and variation analyses, projecting into model organism interologs network, etc...

Computational Genomics Lab UB

Universitat de Barcelona
Genetics, Microbiology & Statistics Department

Institut de Biomedicina de la UB

Barcelona, September 5th, 2019.

Dear *Database* journal Editor;

Please find enclosed the second reviewed version of the manuscript entitled *"RPGeNet v2.0: expanding the universe of retinal disease gene interactions network."* by Rodrigo Arenas-Galnares, Sergio Castillo-Lara, Vasileios Toulis, Daniel Boloc, Roser Gonzàlez-Duarte, Gemma Marfany, and Josep F. Abril.

Just before getting into details, we would like to note that two reviewers, ref. 2 and ref. 3, were already satisfied with our response to the first round of review, and they were in agreement to publish this manuscript. We did our best to provide an accurate answer to the other reviewer; we thank referee 1 for his/her comments, yet we think he/she misunderstood the way supporting evidences are integrated into the `RPGeNet` core network. However, we have also considered that this point was probably not enough detailed in the previous revision round and, on the other hand, the corresponding information panels from the web interface were not clear enough for that purpose either.

We provide some extra supplementary tables in this new release of the manuscript to clarify the origin of the evidences and the merged interactions. Simultaneously, improving the web interface was a little bit time-consuming, as it implied that we had to implement extra functionality to our application—in order to ensure we could easily clarify some of the referee 1 concerns—: to extend the pre-processing protocol scripts to include information about the supporting evidences for the edges—instead of keeping just the evidence counts in the intermediate output files, as we were doing earlier—; to adapt the database records to manage those new data fields; and, finally, we had to extend some features on the web interface to deal with such new information—which improved how such information is available from interaction information pop-up cards too—. Moreover, the first step allowed us to spot a bug in the initial protocol scripts, while the latter drove us to detect another in a query to the database to provide the interactions at a given level for the web-interface. All the reported bugs are now fixed and the application remains fully functional.

We did our best to fulfill all the requests made by referee 1, and we are providing the corresponding reply to their questions and suggestions. We believe this contributed to improve the quality and readability of both, the manuscript and the web interface. You will find further details on our reply to the referees, also attached to this letter. We will appreciate if you can consider our manuscript new version, still describing a major upgrade to our retinal dystrophies interaction network and its web interface (RPGeNet), worth of publishing on your journal. As we stated in our previous letters, we think that the manuscript can be a milestone to be cited when describing some candidate pathways that are under experimental validation by Dr Marfany lab for instance. The upgraded tool is already publicly available as it was the former version, from the following address: `https://compgen.bio.ub.edu/RPGeNet/`

Thank you for your time in considering our paper. We look forward to hearing from you at your convenience.

Yours sincerely,

Josep F Abril, PhD.
Aggregate Professor
Dept. of Genetics, Microbiology & Statistics
Universitat de Barcelona
Av. Diagonal 643, 08028 Barcelona, Catalonia, Spain.
E-mail: jabril@ub.edu – Telf: +34 934035305

For Peer Review

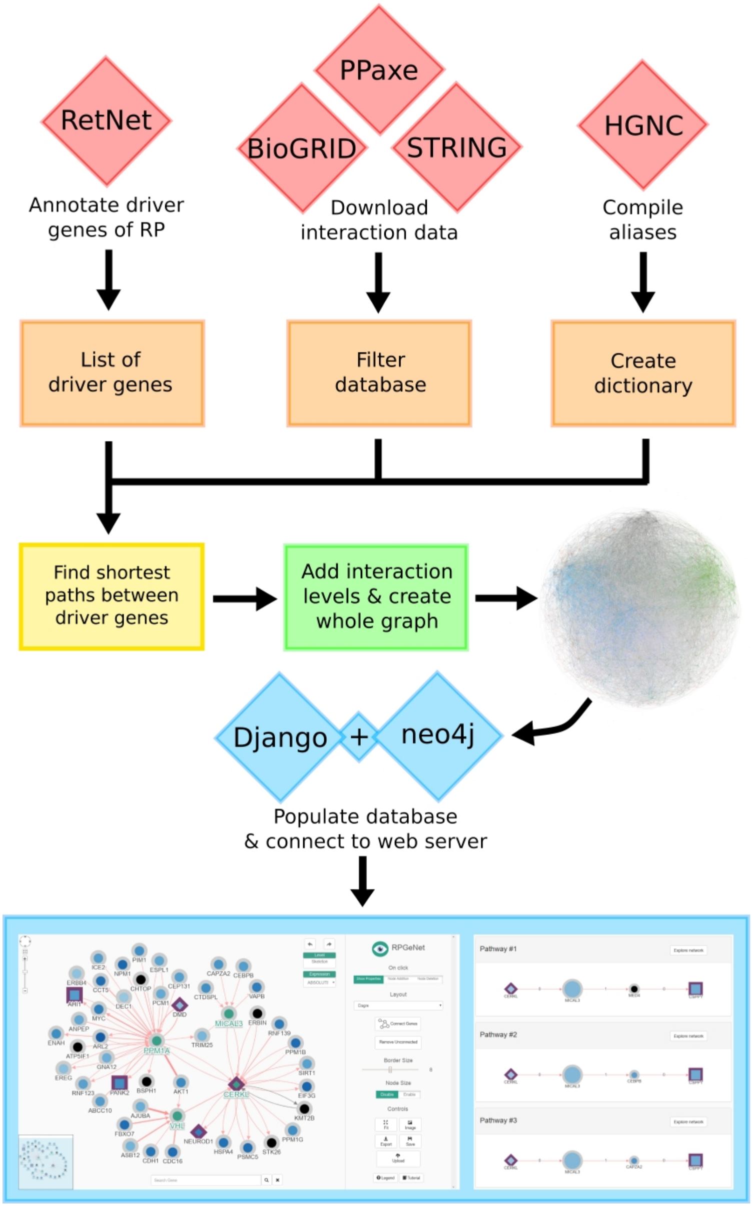

Figure 1.- Data integration workflow to build the RPGeNet core database.
The final graphical web interface (bottom panels) depends on a series of data integration steps that provide the interactions and nodes to the main neo4j database engine. Each component of the workflow is described in detail on the main text.

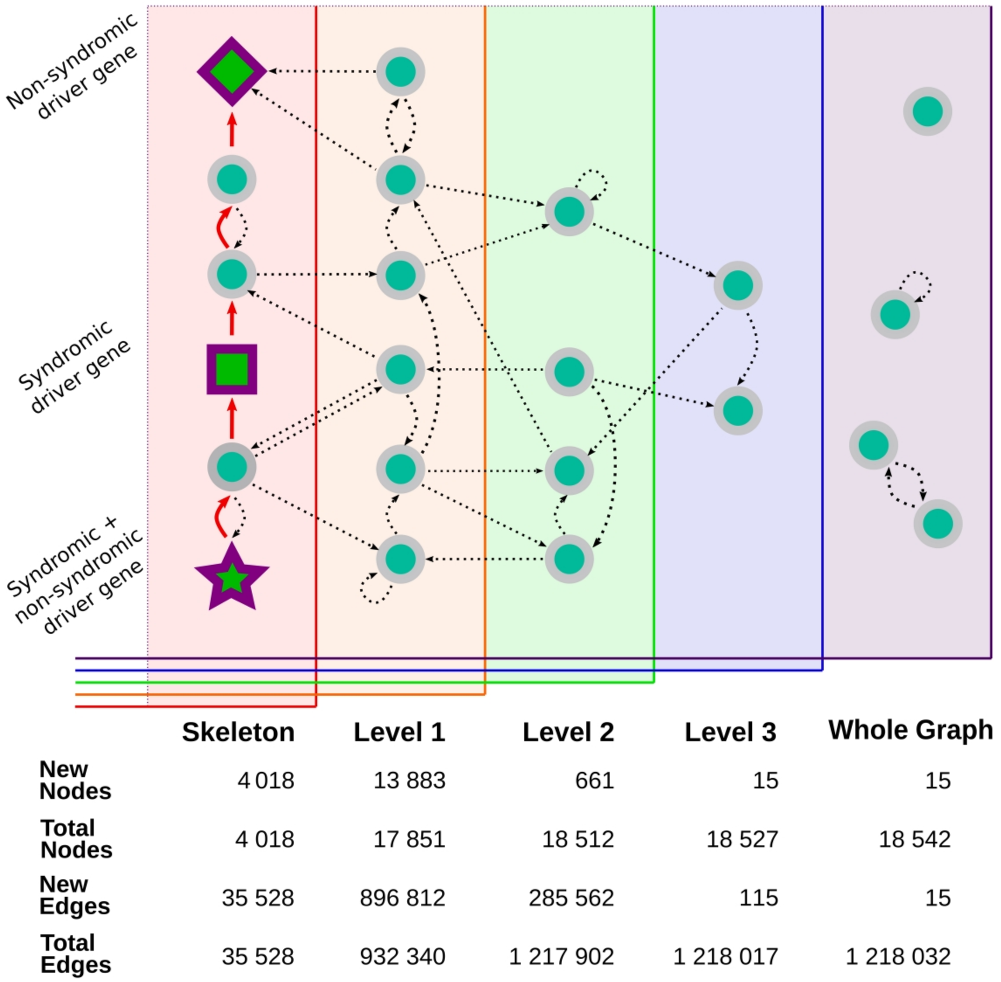

	Skeleton	Level 1	Level 2	Level 3	Whole Graph
**New Nodes**	4 018	13 883	661	15	15
**Total Nodes**	4 018	17 851	18 512	18 527	18 542
**New Edges**	35 528	896 812	285 562	115	15
**Total Edges**	35 528	932 340	1 217 902	1 218 017	1 218 032

Figure 2.- Visual representation of the expansion of the core interactions graph. The graph builder begins with the construction of the skeleton graph, represented by all the nodes on the leftmost panel. The skeleton is created by finding the shortest direct interaction paths (the red arrows) between all the known driver genes—drawn here using the same shapes as in RPGeNet Network Explorer (star, square, and diamond shapes, based on whether their mutations cause or not syndromic diseases)—. The graph is then expanded into level 1 (represented as the orange panel) by adding all of the parents and children (straight lines) of the nodes already found in the skeleton graph. Level-specific interactions are shown as curved connections linking nodes within a given graph level. The expansion is repeated until the highest level is reached and all known genes with known interactions have been connected to the interactions core graph. The remaining genes that do not have any known interaction that connects them with the core graph are included in the whole graph level. Some of those genes may have interactions with other genes found only in the whole graph level (and many of those interactions are self-references). The bottom table from this figure compares the number of nodes and edges added on each level (“New Nodes” and “New Edges” rows), as well as the total number of nodes and edges accumulated.

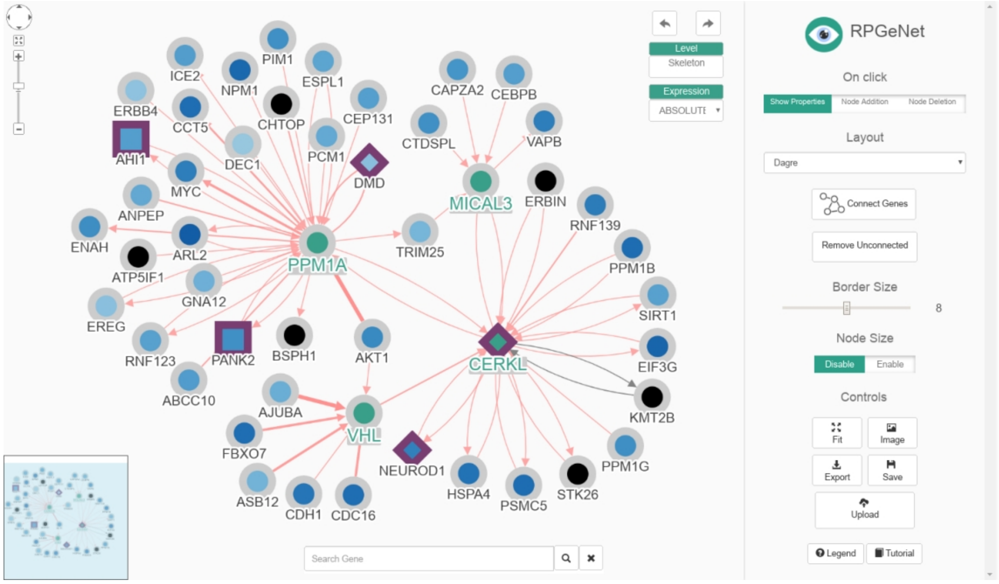

Figure 3.- The renewed Network Explorer interface of RPGeNet.
In this example, the Network Explorer interface shows a subgraph containing all the genes that directly interact with CERKL within the skeleton subnetwork, after expanding nodes for PPM1A, VHL, and MICAL3 (those selected four nodes highlighted in green, driver genes border in purple, node colors based on the "ABSOLUTE" gene-expression data). Some improvements to the interface can be appreciated: a "search" gene add-on at the mid-bottom, an expression data set selection drop-down menu at right-top corner of the network visualization canvas, as well as the "undo"/"redo" buttons. A more dynamic "buttons panel" on the right facilitates the interaction with the network data. Finally, the coloured-by-type interactions also provide directionality information with arrow heads, and have reliability-score proportional widths adjusted to the number of evidences supporting them. This figure can be reproduced on the Network Explorer if users upload the Supplementary File 1.

509x294mm (72 x 72 DPI)

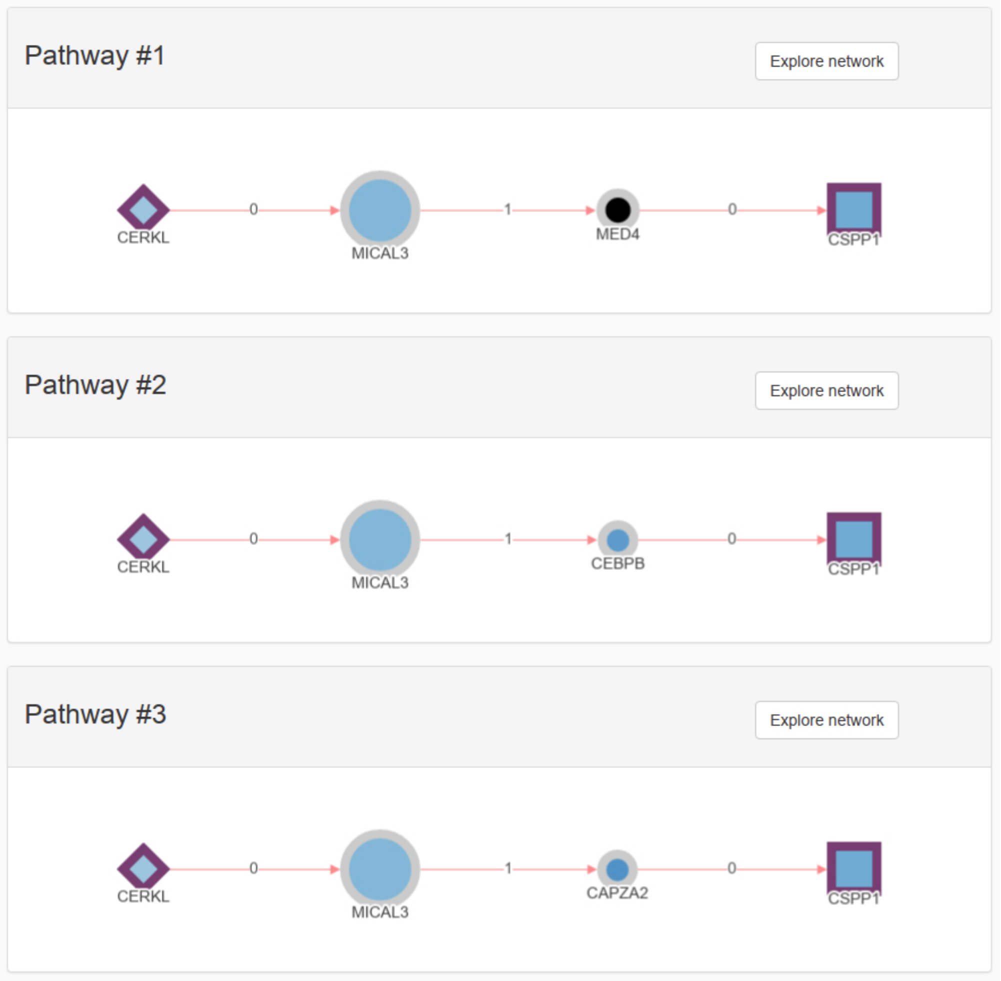

Figure 4.- Example of a pathways list returned using the shortest pathways RPGeNet query. CERKL and CSPP1 where used to start the pathway search on the main RPGeNet form. Only the first three pathways of the 28 retrieved by that query are shown on this figure, all of them at the shortest path length of three (3 edges and 2 nodes between the chosen identifiers). By clicking on the corresponding “Explore Network” button on any of the listed pathways, users can easily jump to the Network Explorer interface to work on the selected genes for that pathway.

311x305mm (72 x 72 DPI)

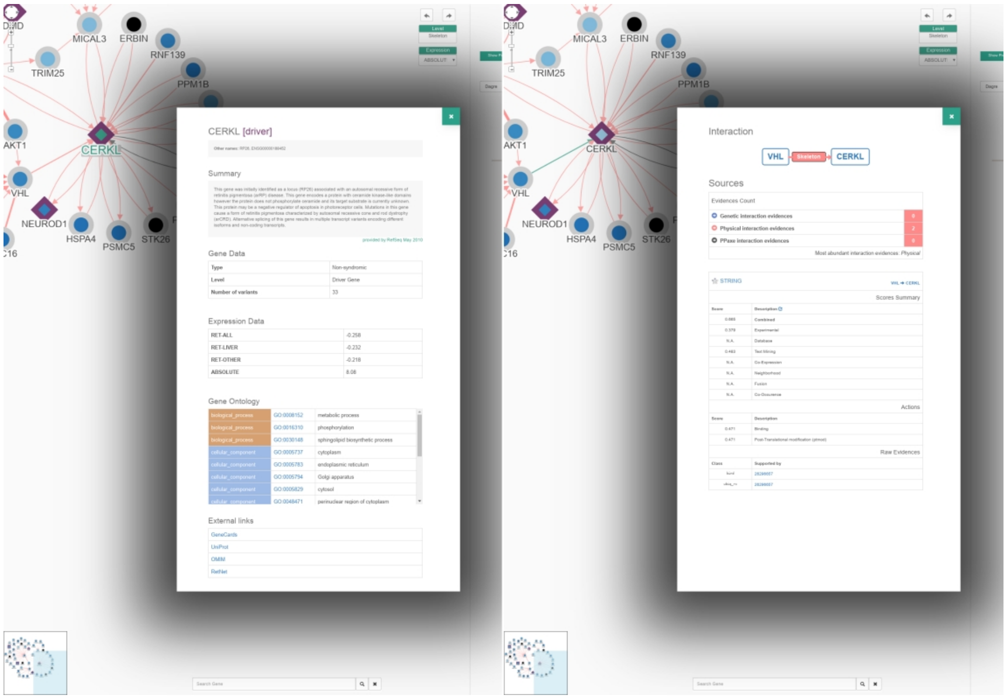

Figure 5.- An example of node (left) and interaction (right) information panels from the Network Explorer interface.

The default behaviour "On click" of the Network Explorer interface is to show "node properties" (see topmost controls on the right panel of that interface on previous figure). From the network example of Figure 3, when clicking at the CERKL node the gene/protein information panel pops up to display a description of the gene, known aliases, a summary of its expression levels and functional annotation, and links to external references. On the other hand, by clicking on an edge, VHL to CERKL in this example, the interaction panel pops up, providing information about the type of possible interactions (genetic [blue], physical [red], or "unknown" [black]), as well as a series of tables containing details about the supporting evidences from the distinct sources, along with the corresponding external links to the reference databases and to the supporting evidences when possible.

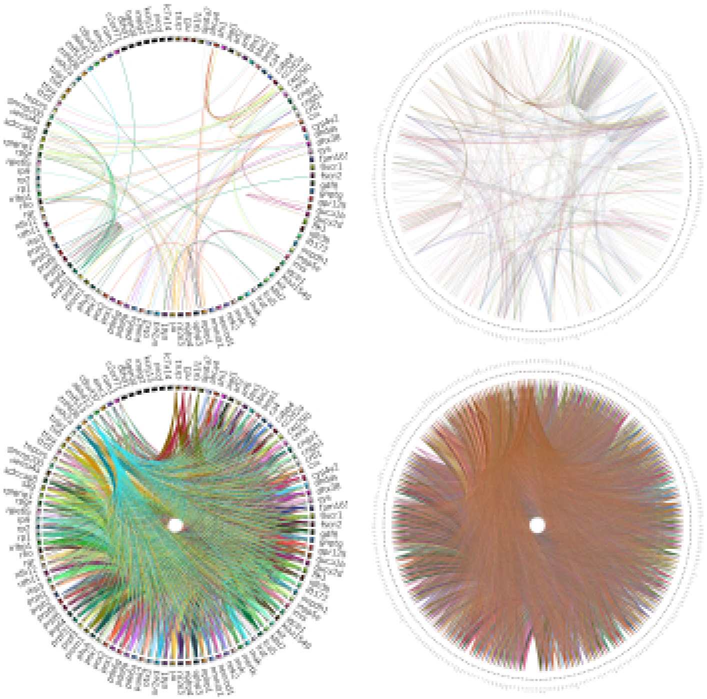

Figure 6.- Analysis of RPGeNet v1 and v2 networks connectivity with Circos (19).
The figure compares the connectivity of the driver genes of the old RPGeNet v1.0 (left) and the updated RPGeNet v2.0 (right). The top pair of plots show all the shortest paths between driver genes at distance one, meaning direct interaction between each pair of driver genes. The bottom par of plots provide the comparison at distance 3, meaning there are two genes in the shortest path between a pair of driver genes of interest. It is clear from the visualized Circos plots that the updated RPGeNet database has a highly connected network.

130x125mm (72 x 72 DPI)

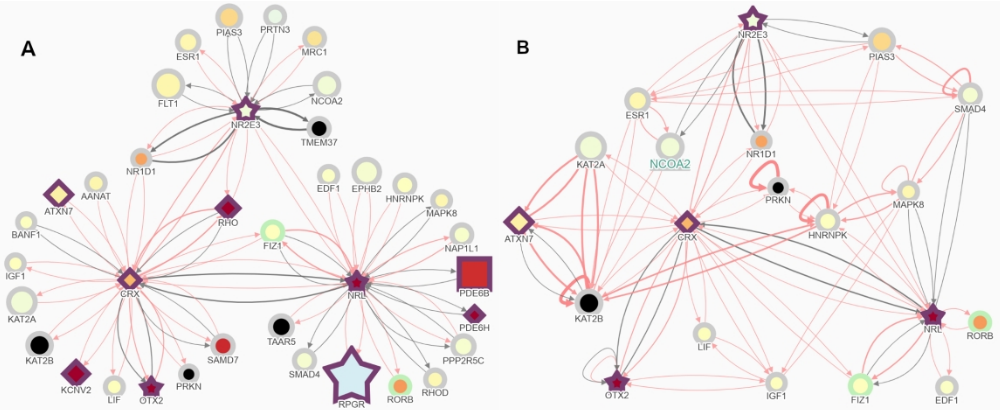

Figure 7.- A control case interaction visualized on RPGeNet showing the connections between NRL, NR2E3 and CRX retinal transcription factors.

A) RPGeNet was queried to display the subnetwork among NRL, NR2E3 for the nodes at distance one at level 1; then nodes connected to CRX were added with the "Node Addition" button activated on click. CRX, NRL, and NR2E3 are three well-known transcription factors that co-regulate retinal-specific genes, among them RHO. Interestingly, several other genes that cause retinal dystrophies also appear in the network as target genes or other transcriptional regulators (border shown in purple). Nodes color-fill defined by the "RET-ALL" gene-expression data.

B) Further trimming of the subnetwork obtained by omitting the nodes that are not chromatin remodelers or transcription factors provide an overview of relevant co-regulators of retinal genes. After deleting the corresponding nodes, further edges—out of the shortest paths that link the genes left—were shown by clicking on the "Connect Genes" button at the control panel. This visualization allows pinpointing and exploring alternate pathways that connect the initial seeds; for instance, CRX and NRL were already connected, but longer paths are now evident like CRX⇌PRKN⇌HNRNPK⇌NRL, when PRKN and HNRNPK are linked. Another example can be the pathway found between NRL and NR2E3 on the path NRL⇌SMAD4⇌PIAS3⇌NR2E3, when SMAD4 and PIAS3 are linked. Both panels from this figure can be reproduced on the Network Explorer if users upload the Supplementary Files 2 and 3, respectively.

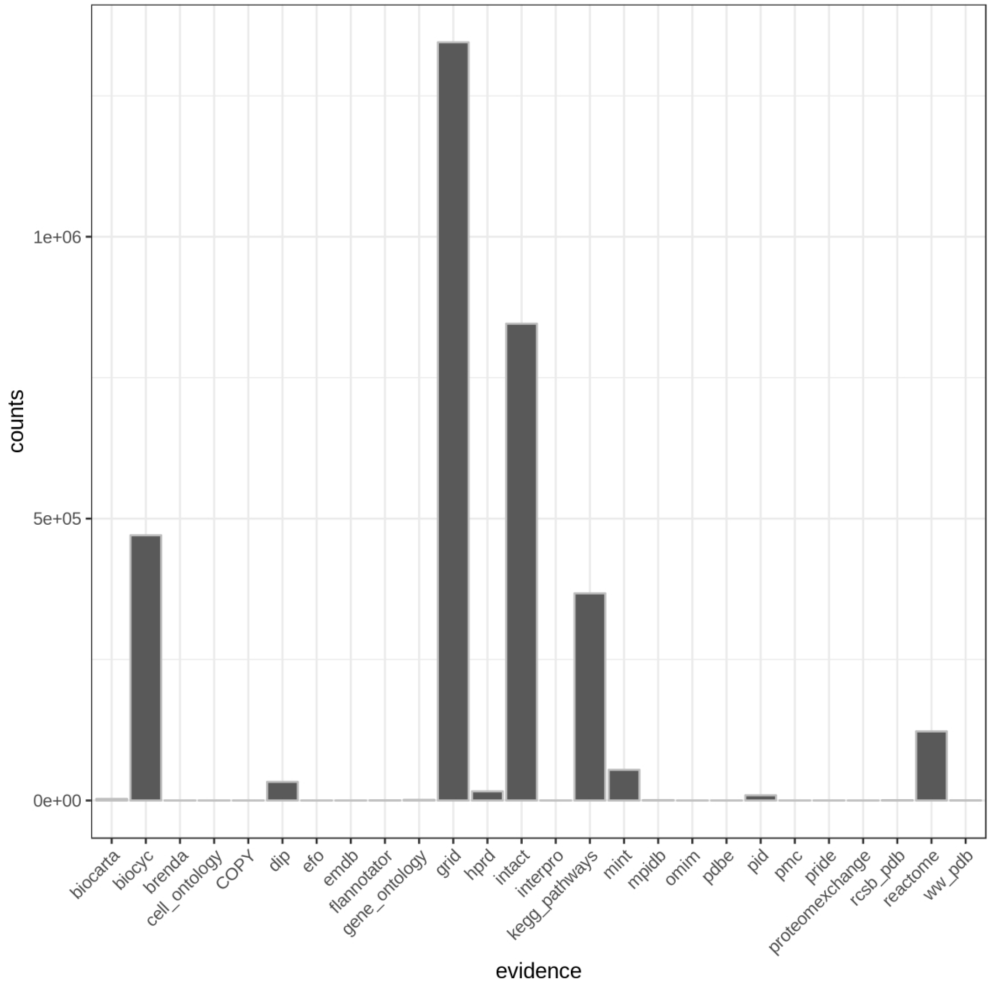

Supplementary Figure 1.- Bar plot with distribution of the different evidences used by STRING. STRING is a protein-to-protein interactions database that includes predictions and experimentally validated interactions. The experimentally validated interactions include evidences from different sources. The proportion of such evidences is shown in this bar-plot. GRID, INTACT, KEGG, BIOCARTA and REACTOME are the most common sources of experimentally-validated interactions from STRING.

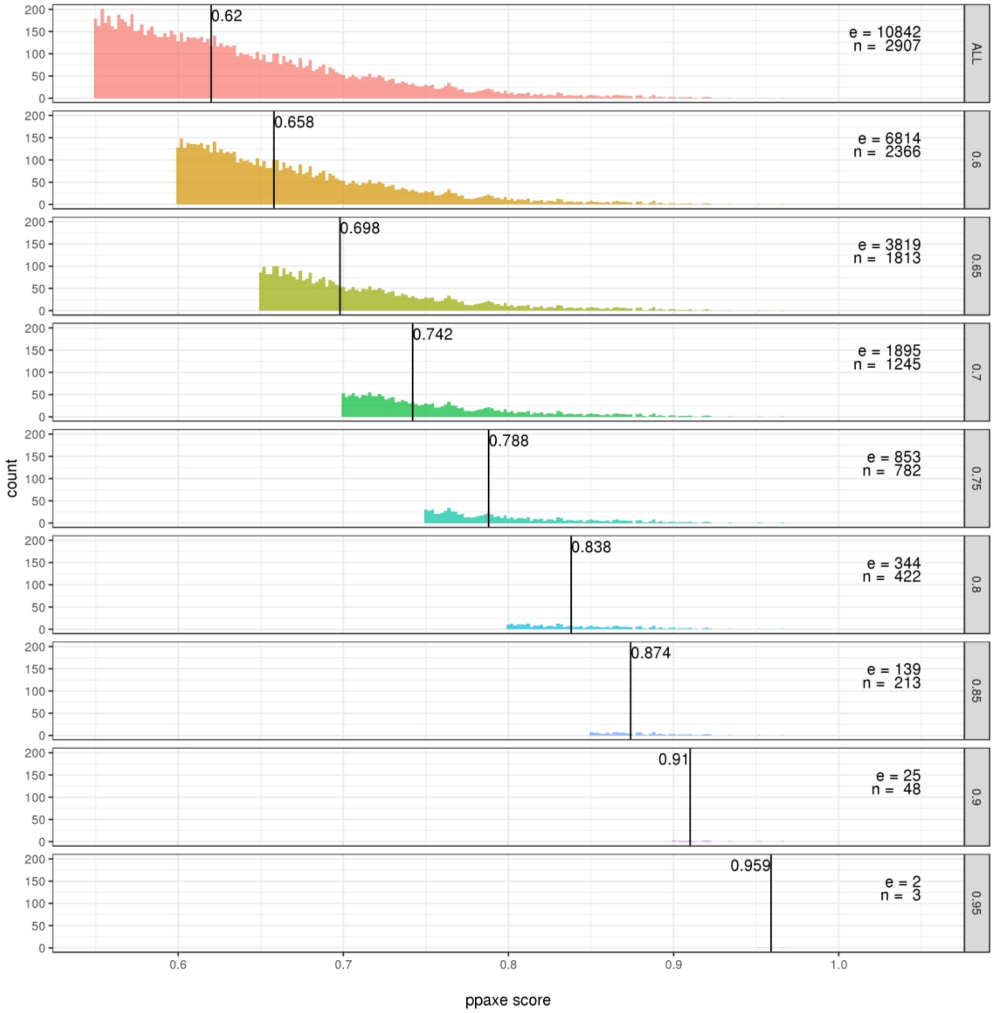

Supplementary Figure 2.- Choosing the optimal votes cut-off for PPaxe.
The graph shows the distribution of the un-normalized confidence score given to the interactions detected by PPaxe with respect to the number of PPaxe interactions (counts), and the average score depending on the cut-off score chosen (vertical line). In an attempt to optimize the number interactions while reducing false positive interactions and increasing false-negatives, the cut-off score chosen was 0.65, which was the value that yielded a minimum precision of 90% in the validation assessment performed in the original PPaxe manuscript (6). "e" and "n" correspond to the number of edges and nodes respectively that are passing the cut-off threshold. Panels categorize by the percentage of votes by the random-forest classifier.

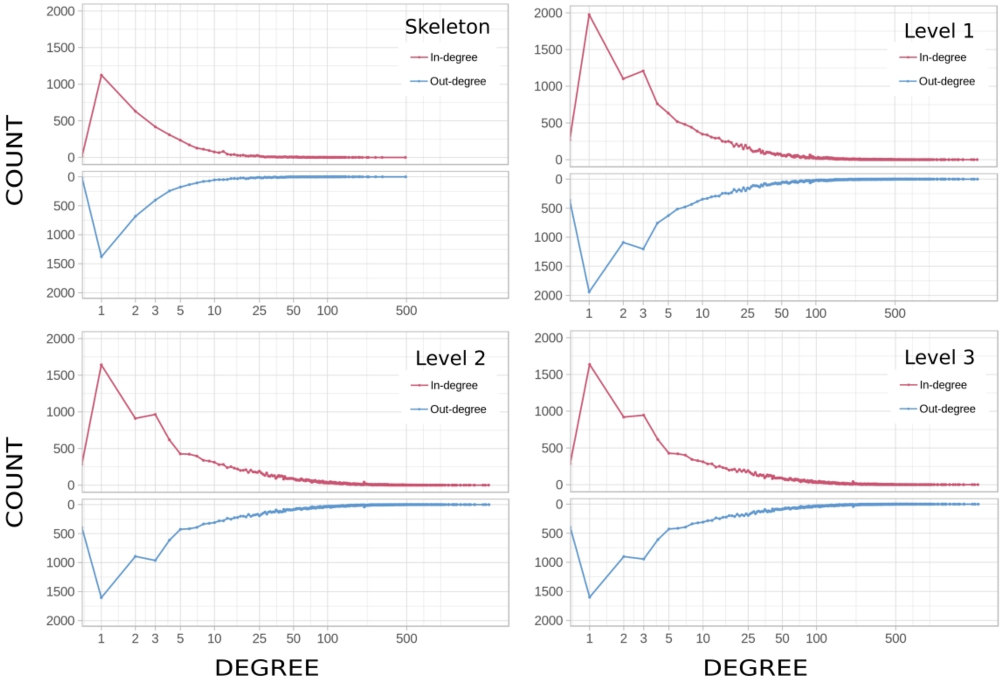

Supplementary Figure 3.- Comparing the number of counts between in and out degrees for each level of the RPGeNet core network.

In the skeleton graph the much smaller area between the in-/out-degree lines in comparison to higher level graphs denotes a smaller number of interactions. Comparing the skeleton with level one, there is a large increase in counts for lower degrees. However, as the graph is expanded to levels two and three, a decrease of counts in lower degrees can be observed. This can be explained by a larger jump in total number of interactions between skeleton and level one, followed by smaller increments in new interactions and a slower increase in degree from levels one to three, as new nodes and interactions are introduced.

GRAPH STATS	RPGeNet v1		RPGeNet v2 — All Sets					BioGRID Only		STRING Only		PPaxe Only	
	Skeleton	WholeGrap	Skeleton	Level1	Level2	Level3	WholeGrap	Skeleton	WholeGrap	Skeleton	WholeGrap	Skeleton	WholeGrap
**Total #Nodes**	1 294	22 372	4 018	17 851	18 512	18 527	18 542	3 057	15 205	2 555	13 325	762	3 146
**Isolated Nodes**	7	7	16	15	15	15	15	71	70	56	56	93	84
**Adjacent Drivers**	103 of **110**		260 of **276**	261 of **276**				205 of **276**	206 of **276**	220 of **276**		183 of **276**	192 of **276**
**Total #Edges**	5 883	752 062	35 528	932 340	1217 902	1 218 017	1 218 032	23 480	623 601	20 791	629 271	3 654	13 584
**Mutual** [A⇌B]	1 082	319 928	9 601	462 988	604 652	604 707	604 713	6 243	307 422	6 422	314 629	1 554	6 760
**Assymetric** [A→B]	3 719	106 907	16 326	5 074	5 931	5 931	5 931	10 994	6 138	7 947	0	546	0
**Self-loop** [A→A]	0	5 299	0	1 290	2 667	2 672	2 675	0	2 619	0	13	0	64
**Total Non-Redundant**	4 801	432 134	25 927	469 352	613 250	613 310	613 319	17 237	316 179	14 369	314 642	2 100	6 824
**Graph Density**	0.0035	0.0015	0.0022	0.0029	0.0036	0.0035	0.0035	0.0025	0.0027	0.0032	0.0035	0.0063	0.0014
**Avg. Clustering**	0.0433	0.1445	0.0613	0.1449	0.2331	0.2331	0.2331	0.0448	0.0737	0.1508	0.5512	0.0959	0.0580
**Graph Diameter**	10	9	8	6	7	8	8	8	7	9	10	13	13
**Graph Reciprocity**	0.3678	0.8568	0.5405	0.9946	0.9951	0.9951	0.9951	0.5318	0.9901	0.6178	1.0000	0.8506	1.0000
**Avg. Degree**	9.0927	67.2324	17.6844	104.458	131.5797	131.4856	131.3809	15.3615	82.0258	16.2748	94.4497	9.5906	8.6357
**Avg. Closeness**	0.0969	0.0185	0.052	0.0529	0.0529	0.0529	0.0295	0.0128	0.0116	0.0165	0.0052	0.0093	0.0024
**Betweenness**	3	32 888.11	10	33	34	35	35 044.91	7	28 684.70	6	31 567.57	1 635.56	7 714.01
**Avg. Edge Betw.**	1	1 480.75	1 629.48	980.37	812.19	814.28	814.27	1	1 064.77	1	942.69	463.38	2 354.06
**Avg. Coreness**	4.7751	39.8983	9.1309	54.8258	77.0557	77.0063	76.9456	7.9326	42.0278	8.7366	67.3668	5.1273	4.6300
**Avg. Eccentricity**	6.7272	4.5465	5.6904	4.5666	4.9552	5.8732	5.8691	5.5741	5.1786	6.2693	6.6453	6.8307	7.9259
**Avg. Path Length**	4.1559	2.9473	3.6155	2.8810	2.8969	2.9000	2.9000	3.5936	2.9143	3.8110	3.4375	3.7888	4.1479

Supplementary Table 1.- Topology of the `RPGeNet` interactions graph.

This table shows the total number of nodes (genes/proteins, where adjacent nodes can be calculated by substracting *isolated* from *total nodes*) and edges (interactions, including counts by the type of relation between pairs of adjacent nodes: mutual-, asymmetric- and self-interactions), for every level of the `RPGeNet` database graph (see "`RPGeNet v2` All Sets" block). For comparison purposes, graph stats are provided for the previous `RPGeNet v1` version, as well as those for the interaction networks that can be produced separately from each of the evidence sources (blocks named as "`BioGRID` Only", "`STRING` Only", and "`PPaxe` Only", respectively). Most of the *isolated nodes* correspond to drivers without interaction evidences from the selected sources, and those numbers complement the values from the *adjacent drivers* row. Graph statistics were described in depth on Newman *et al* [1]. In br: *graph density* is the ratio between edges and the total number of possible vertices (nodes); *average clustering coefficient* (or the tendency of nodes to cluster together) is a measure of how complete the neighborhood of a node is, over all the nodes of the network; *diameter* is the maximum distance between two nodes; *reciprocity* is the; *average degree* is the average number of edges per node; *closeness* is the average length of shortest path between a node and every other node in the network; *betweenness* is a measure of the number of times a node is found within the shortest paths between two other nodes; *coreness* defines the shell index of the vertices of a network; *eccentricity* is the maximum of the shortest distances of a node with respect all other nodes in the graph; finally, *average path length* is a measure of the average distance between two nodes. Graph stats produced with `python-igraph` library (v0.7.0, see further details at https://igraph.org/python/).

1. Newman MEJ, Barabási A-L, Watts DJ. The structure and dynamics of networks. 2006. p. 624

Section		Skeleton	Level1	Level2	Level3	WholeGraph
GRAPH SUMMARY	**Total UNIQUE NODES**	4 018	17 851	18 512	18 527	18 542
	Adjacent Nodes	4 002	17 836	18 497	18 512	18 527
	Isolated Nodes	16	15	15	15	15
	**NODES by Source**					
	BioGRID	3 677	14 831	15 132	15 136	15 139
	STRING	3 580	12 864	13 253	13 263	13 269
	PPaxe	1 407	3 016	3 054	3 056	3 062
	TOTAL	8 664	30 711	31 439	31 455	31 470
	Nodes Source "redundancy"	*215.63%*	*172.04%*	*169.83%*	*169.78%*	*169.72%*
	**Total DIRECTED EDGES**	35 528	932 340	1 217 902	1 218 017	1 218 032
	Mutual [A⇄B]	9 601	462 988	604 652	604 707	604 713
	Assymetric [A⇀B]	16 326	5 074	5 931	5 931	5 931
	Self-loop [A⇀A]	0	1 290	2 667	2 672	2 675
	Total Non-Redundant	25 927	469 352	613 250	613 310	613 319
	Directed Edges "redundancy"	*137.03%*	*198.64%*	*198.60%*	*198.60%*	*198.60%*
	**EDGES by Source**					
	BioGRID all	22 914	518 478	623 643	623 656	623 659
	BioGRID only	*21 334*	*483 667*	*579 207*	*579 220*	*579 220*
	STRING all	12 563	440 111	629 167	629 265	629 271
	STRING only	*10 599*	*402 920*	*582 094*	*582 190*	*582 190*
	PPaxe all	2 277	12 282	13 572	13 578	13 584
	PPaxe only	*1 534*	*8 049*	*8 984*	*8 988*	*8 988*
	TOTAL	37 754	970 871	1 266 382	1 266 499	1 266 514
	Edges Source "redundancy"	*145.62%*	*206.85%*	*206.50%*	*206.50%*	*206.50%*
EVIDENCE SUMMARY	**TOTAL EVIDENCES**	75 447	2 371 305	3 209 677	3 209 856	3 209 871
	**By Class**					
	Genetic evidences	257	6 018	7 062	7 063	7 063
	Avg. evids x directed edge	*0.007*	*0.006*	*0.006*	*0.006*	*0.006*
	Physical evidences	70 561	2 342 154	3 177 567	3 177 739	3 177 748
	Avg. evids x directed edge	*1.986*	*2.512*	*2.609*	*2.609*	*2.609*
	Unknown evids (PPaxe)	4 629	23 133	25 048	25 054	25 060
	Avg. evids x directed edge	*0.130*	*0.025*	*0.021*	*0.021*	*0.021*
	**By Source**					
	BioGRID	31 726	705 485	842 798	842 815	842 818
	Physical interactions	*31 469*	*699 467*	*835 736*	*835 752*	*835 755*
	Genetic Interactions	*257*	*6 018*	*7 062*	*7 063*	*7 063*
	Avg. evids x directed edge	*0.893*	*0.757*	*0.692*	*0.692*	*0.692*
	STRING	39 092	1 642 687	2 341 831	2 341 987	2 341 993
	Avg. evids x directed edge	*1.100*	*1.762*	*1.923*	*1.923*	*1.923*
	PPaxe	4 629	23 133	25 048	25 054	25 060
	Avg. evids x directed edge	*0.130*	*0.025*	*0.021*	*0.021*	*0.021*
EDGES with STRING SCORE	**With any STRING score**	**12 563**	**440 111**	**629 167**	**629 265**	**629 271**
	With "experimental" score	2 696	75 075	109 191	109 231	109 235
	With "database" score	9 112	371 666	541 032	541 082	541 084
	With "text-mining" score	8 803	248 133	345 610	345 682	345 686
	With "co-expression" score	2 227	107 246	157 974	158 008	158 010
	With "neighborhood" score	0	0	0	0	0
	With gene-"fusion" score	16	1 136	2 384	2 384	2 384
	With "co-occurrence" score	124	6 320	8 749	8 755	8 757

Supplementary Table 2.- Source origin and redundancy of evidences for RPGeNet core network interactions.
First block accounts for the number of nodes and edges supported by each input source at each graph level of the core network. Second block provides information about distinct evidences used to weight the interactions. Number of interactions on the core network with STRING scores is provided on the last block to complement evidences (also shown in the interaction information pop-up cards like the one shown in Figure 5 right panel). RPGeNet models interactions as directed edges between nodes, and thus, represents undirected interactions between genes as two separate interactions. The "Total Interactions" column shows the total number of edges stored in the database (without taking into account the number of evidences), counting reciprocal interactions twice (A⇀B is different from B⇀A). The "Non-redundant Interactions" column refers to the number of interactions in RPGeNet independently of the direction, and thus, the pair A⇀B and B⇀A is only counted once. The Non-redundant interactions count criteria is equivalent to the one used by the BioGRID database (https://wiki.thebiogrid.org/doku.php/build_3.5.171).

	Skeleton		WholeGraph	
	Total # Nodes	Total # Edges	Total # Nodes	Total # Edges
**Run Together (RPGeNet v2)**	4 018	35 528	18 542	1 218 032
**Overlapping Sets**	4 767	46 610	18 539	1 217 974
**BioGRID only**	2 031	22 757	4 956	579 165
**STRING only**	1 389	19 650	2 598	582 196
**PPaxe only**	136	2 994	113	8 994
**BioGRID ∩ STRING**	585	549	7 839	43 029
**BioGRID ∩ PPaxe**	45	68	145	544
**STRING ∩ PPaxe**	185	486	623	3 183
**BioGRID ∩ STRING ∩ PPaxe**	396	106	2 265	863
**Total from BioGRID**	3 057	23 480	15 205	623 601
**Total from STRING**	2 555	20 791	13 325	629 271
**Total from PPaxe**	762	3 654	3 146	13 584

Supplementary Table 3.- Interaction sources overlap against the RPGeNet skeleton and wholegraph networks.

To provide an estimate of the overlap for the evidences gathered from each source, this table shows the intersection of total number of nodes and edges for the skeleton and wholegraph networks produced when the RPGeNet pipeline is run over each of the interactions sources separately. First row has the totals for the whole network that is produced when combining all the sources when running the full pipeline to create the graph levels integrated on the RPGeNet v2 database, already shown on the previous Supplementary Tables. See also Supplementary Table 4 for further details about edges defined from the analysis of each separate interaction sources.

	Edges Summary				
	Total Directed	Mutual [A⇌B]	Assymetric [A⇀B]	Self-loop [A⇀A]	Total Non-Redundant
**BioGRID**					
Skeleton	23 480	6 243	10 994	0	17 237
WholeGraph	623 601	307 422	6 138	2 619	316 179
**STRING**					
Skeleton	20 791	6 422	7 947	0	14 369
WholeGraph	629 271	314 629	0	13	314 642
**PPaxe**					
Skeleton	3 654	1 554	546	0	2 100
WholeGraph	13 584	6 760	0	64	6 824
**RPGeNet v2 Core Network**					
Skeleton	35 528	9 601	16 326	0	25 927
WholeGraph	1 218 032	604 713	5 931	2 675	613 319

Supplementary Table 4.- Edges classification for the standalone pipeline analyses over each separate interaction sources versus RPGeNet core network.

The "Total Interactions" column shows the total number of edges stored in the database (without taking into account the number of evidences), counting reciprocal interactions twice (A⇀B is different from B⇀A). The "Non-redundant Interactions" column refers to the number of interactions in RPGeNet independently of the direction, and thus, the pair A⇀B and B⇀A is only counted once. Further details on all graph levels for the RPGeNet core network are provided on Supplementary Table 2.

**Driver Genes**	**Predicted Interactions**	**Putative Interactors**
DTHD1 *	10	*AIPL1, CHD3, CNGB1, IFI27, MACC1, PID1, SH3BP4, UNC5A, UNC5C, UNC5CL*
IMPG2 *	10	*C2orf71, C8orf37, EYS, FAM161A, PRCD, TMEM252, TTC8, TULP1, ZNF408, ZNF513*
KCNJ13 *	10	*COL11A1, COL4A3, EDNRB, GJA5, IGSF11, KCNE3, KCNK5, KCNN4, KCNQ1, PNP*
MIR204	0	-
MT-TH	0	-
MT-TL1	0	-
MT-TP	0	-
PLA2G5	10	*ALOX5, ENSG00000168970, PLA2G15, PLA2G16, PLA2G4D, PLA2G7, PLD1, PLA2G4A, PTGS1, PTGS2*
PRCD *	10	*C2orf71, CERKL, CNGB1, FRMD6, IMPG2, PDE6A, PDE6B, RPE65, SLC4A3, ZNF513*
RAX2	10	*AKTIP, C8orf37, CRX, DPRX, FIZ1, GUCA1A, LEUTX, NRL, PITPNM3, PLAC9*
RDH12	10	*RBP1, RBP2, RBP5, RETSAT, ALDH1A1, ALDH1A2, LRAT, AOX1, CYP26A1, BCO1*
SLC7A14 *	10	*ANKS4B, CAPN9, EIF5A2, GART, MFSD9, NMNAT2, RASD2, RPL22L1, SLC15A5, SLCO1B7*
SRD5A3	10	*SRD5A1, AKR1C1, AKR1C2, AKR1C3, AKR1D1, DOLK, HSD3B2, HSD17B3, HSD17B6, CYP17A1*
TRNT1	10	*ACOT13, ELAC1, ELAC2, FARS2, LARS2, PNPT1, TRIT1, TRMT10C, YARS, YARS2*
TTPA	10	*ATCAY, BNIP2, CYP4F2, EIF3K, FXN, PRUNE2, SLC6A11, SRR, RRS1, TRIM37*
C1QTNF5 (*CTPR5*)	4	*PATE4, TNFAIP8L1, ENSG00000235718, ENSG00000259159*

Supplementary Table 5.- Predicted interactions for unconnected drivers genes on RPGeNet core network.

Driver genes can be unconnected to the core interactions whole-network graph because there are no known experimentally validated interactions that link them to the rest of nodes from the whole-network graph. Number of predicted interactions and putative interactors were retrieved from STRING database. The predicted interactions were not included into the whole core network on this release. Bottom row shows a driver gene, *C1QTNF5* (formerly identified as *CTPR5*), which is aggregated by the protocol to the growing graph at level 1 expansion yet it only has a validated interaction to itself from that point to the whole-graph. From seven unconnected driver genes of the RPGeNet previous version (2), five are still listed on this table—those marked with * —, while the updated interaction evidences made possible to connect the other two (*HGSNAT* and *PCARE*, the latter formerly identified as *C2ORF71*).

**Non-Driver Genes**	**Predicted Interactions**	**Putative Interactors**
DEFB106A	10	*DEFB103A, DEFB103B, DEFB104A, DEFB104B, DEFB105A, DEFB105B,* ***DEFB106B****, DEFB107A, DEFB107B, DEFB108B*
DEFB106B	10	*DEFB103A, DEFB103B, DEFB104A, DEFB104B, DEFB105A, DEFB105B,* ***DEFB106A****, DEFB107A, DEFB107B, DEFB108B*
DFNB32	0	-
DFNA37	0	-
MFSD2A	10	*CDC5L, ERVFRD-1, KIAA1919, MFSD5, MFSD10, MFSD11, SLC25A33, SVOP, SVOPL, SYSP2L*
MFSD2B	10	*AP1S3, C9orf66, C16orf59, C16orf70, DDRGK1, EXOC3L4, MYLK3, RFX4, SAMD14, SUSD1*
PDSS1	10	*COQ2, COQ6, COQ9, DHDDS, FDPS, FDFT1, FNTA, FNTB, GGPS1,* ***PDSS2***
PDSS2	10	*COQ2, COQ6, COQ9, DHDDS, FDPS, FDFT1, FNTA, FNTB, GGPS1,* ***PDSS1***
SLC22A7	10	*ABCB11, ABCG2, ENSG00000257046, SLC10A1, SLC22A8, SLCO1A2, SLCO1B1, SLCO1B3, SLCO2B1, UCK2*
SLC22A10	10	*OR5AR1, OR10X1, SLC7A13, SLC28A1, SLC35G4P, SLCO1A2, SLCO1B7, SLCO3A1, SLCO4A1, SLCO4C,*
ADGRL4 (*ELTD1*)	10	*CLEC14A, EMCN, GSTO1, KNG1, LGALS8, MYCT1, NAA10, NEUROD6, PHACTR1, ZNF366*
SLC24A2	10	*CNGA3, CRKL, GUCA1C, HHIP, KCNIP1, MAP3K4, RCVRN, SLC6A1, SH3GL2, TRIM4*
TMEM196	10	*BTBD17, CDCA7L, DNAH11, ITGB8, MACC1, RAPGEF5, SP4, TMEM63C, TMEM184C, TMEM207*

Supplementary Table 6.- Predicted interactions for "unconnected" non-driver genes on `RPGeNet` core network.

Non-driver genes can be unconnected to the core interactions whole-network graph because there are no known experimentally validated interactions that link them from the whole-graph network level to the rest of nodes at any level of the `RPGeNet` core network. Number of predicted interactions and putative interactors were retrieved from `STRING` database. The predicted interactions were not included into the whole core network on this release. The last three genes have self-interactions, while the others are connected only among them in pairs at the whole-graph level (as for instance, *DEFB106A* and *DEFB106B*).

`RPGeNet v2.0`: expanding the universe of retinal disease gene interactions network

Rodrigo Arenas-Galnares[1,2,✓], Sergio Castillo-Lara[1,2,✓], Vasileios Toulis[1,2,3], Daniel Boloc[4], Roser Gonzàlez-Duarte[5], Gemma Marfany[1,2,3,5,*,+] Josep F. Abril[1,2,*,+]

[1] Department of Genetics, Microbiology and Statistics, University of Barcelona, Barcelona, 08028, Catalonia, Spain
[2] Institute of Biomedicine (IBUB), University of Barcelona, Barcelona, 08028, Catalonia, Spain
[3] CIBERER, ISCIII, University of Barcelona, Barcelona, 08028, Catalonia, Spain
[4] Faculty of Medicine, University of Barcelona, Barcelona, 08036, Catalonia, Spain
[5] DBGen Ocular Genomics, Barcelona, 08028, Catalonia, Spain

✓ The authors wish it to be known that, in their opinion, the first two authors should be regarded as joint First Authors.
* To whom correspondence should be addressed. Telf: +34 93 403 1305. Email: jabril@ub.edu
+ The authors wish it to be known that last two authors should be regarded as joint Senior Authors.

ABSTRACT

`RPGeNet` offers researchers a user-friendly queriable tool to visualize the interactome network of visual disorder genes, thus enabling the identification of new potential causative genes and the assignment of novel candidates to specific retinal or cellular pathways. This can be highly relevant for clinical applications as retinal dystrophies affect 1:3000 people worldwide and the causative genes are still unknown for 30% of the patients. `RPGeNet` is a refined interaction network interface that limits its skeleton network to the shortest paths between each and every known causative gene of inherited syndromic and non-syndromic retinal dystrophies. `RPGeNet` integrates interaction information from `STRING`, `BioGRID`, and `PPaxe`, along with retina-specific expression data and associated genetic variants, over a `Cytoscape.js` web interface. For the new version, `RPGeNet` v2.0, the database engine was migrated to `Neo4j` graph database manager, which speeds up the initial queries and can handle whole interactome data for new ways to query the network. Further user facilities have been introduced, as the capability of saving and restoring a researcher customized network layout, or as novel features to facilitate navigation and data projection on the network explorer interface. Responsiveness has been further improved by transferring some functionality to the client-side.

INTRODUCTION

Inherited retinal dystrophies (IRDs) comprise a highly heterogeneous group of disorders caused by over 200 causative genes (1). The prevalence of IRDs is 1:3000 worldwide, which make these blinding disorders a health relevant target. The implementation of massive sequencing approaches has greatly facilitated genetic testing and, as a result, the number of IRD genes and mutations is constantly increasing. Nonetheless, a substantial number of cases remain to be accurately diagnosed, as the average yield in IRD genetic diagnosis is roughly 50%.

Besides technical limitations, one of the bottlenecks in massive sequence-based molecular diagnosis is that most identified variants are previously unreported missense changes of unknown pathogenicity, either in known causative genes or in previously unreported candidates. These variants may be deemed as pathogenic or probably damaging by *in silico* predictive programs but end up classified as VUS (genetic variants of unknown significance), since there are not functional analyses to support their pathogenicity and their relationship to retinal physiology is yet to be determined.

Molecular medicine based on gene and protein networks is rapidly expanding since most disease-causing genes often work together, either forming a protein complex or participating in the same signalling pathways. In contrast to the analysis of isolated genes, finding the networks that link disease candidate genes provides supporting data for the identification of new causative genes, functional clues for assaying putative pathogenic novel variants, as well as opens new scenarios to identify key therapeutical targets.

Comprehensive tools to navigate through gene functions, cellular pathways and pathogenicity begin to emerge, particularly for cancer research. Although a considerable amount of genetic and functional data on IRDs genes and mutations has been gathered, there are not many user-friendly searchable tools to make a network map of gene/proteins interactions. To fill this gap, a web application, `RPGeNet` (2), was implemented that integrated all the physical and genetic interactions at that time for a subset of IRD genes (retinitis pigmentosa and Leber congenital amaurosis), obtained from different databases and including additional data such as tissue-specific expression. The expansion of genetic data, plus all the interactome and other omics information gathered of late, has prompted us to review and expand the initial 100 genes to more than 200 retinal dystrophy genes,

include distilled interaction databases and implement an improved network generation, management, and visualization interface.

TOOL DESCRIPTION

`RPGeNet` is a tool to assist in the search for potential candidate genes and pathways associated with retinitis pigmentosa and intended to be used by both research and genetic diagnostic purposes. `RPGeNet` refines the vast interaction network by reducing it to the shortest paths between known driver genes of the disease (from now on the so-called skeleton network). The reduced network allows users to better identify genes interconnecting drivers that can be directly associated with retinal dystrophies by searching through the shortest paths in the skeleton graph (see workflow schema on Figure 1), instead of the immense number of interactions found in the complete whole network. `RPGeNet` does, however, allow users to expand beyond the skeleton subnetwork if needed. To make searches more practical, `RPGeNet` expands the network by levels until recreating the entire known interactions graph (whole graph). Each level is an expansion in the parents and children of the nodes in the previous level (see Figure 2), and its newly added nodes tend to be either less significant to the disease the higher the level you go or poorly studied genes with few known interactions.

`RPGeNet` now has three distinct types of queries available to undergo such disease-specific pathways characterization. The first query, as in the original `RPGeNet`, is *what interacts directly with the gene of interest?* The user sets one or more gene of interest and the output is a graph with the gene or genes of interest connected with all the genes that interact directly. This graph can then be expanded and its layout manually curated (Figure 3). The second query is *what are all the shortest paths between two genes of interest?* Two genes are provided and a list of all the pathways between those two genes is returned (Figure 4). However, as edges in the pathway are directional, only pathways driving from the first requested gene to the second one are listed. To find possible pathways in the opposite direction, if any, the user should redo the query just switching the order of the query genes. A pathway can then be transferred to the `Network Explorer` where the user can further explore other nodes extending to the retrieved shortest pathway. A third query was included: *is a given gene connected to any disease associated gene?* The idea is to characterize the shortest paths from a gene of interest, not yet related to a disease, to one of the network levels already defined over the driver genes. The new database engine facilitates that kind of query on the whole

graph of interactions with respect to any of the nodes on the skeleton path and upper levels. Like in the previous query, a user can further explore a retrieved pathway from results listing on `Network Explorer`.

CHANGES TO DATABASE

Driver Genes

`RPGeNet` sources its driver genes from online database `RetNet` (1), which has a collection of over 300 mapped loci that are clinically validated with known Mendelian mutations that can cause a retinal disease in humans. Of the over 300 mapped loci, only the 276 identified genes, from now on referred to as driver genes, were chosen to build the skeleton network. That means that the database now handles 166 more driver genes than in the previous version. The increase of driver genes comes with many changes to the network including a more connected whole network and an earlier saturation of the interaction subnetworks when considering parent and child nodes growing from the skeleton network (see Supplementary Table 1 for a summary of graph statistics at different `RPGeNet` levels). Previously, the subnetworks saturated at level four and now saturate at level three. That posed some limitations to the previous implementation of the interface and the management of the network queries, which has been overcome with the new release of `RPGeNet`. All gene identifiers included on the whole network were unaliased to the official HUGO Gene Nomenclature Committee (HGNC) reference symbol (3).

Data Sources

`BioGRID`: (4) The `RPGeNet` v2.0 database has been updated with the interaction data from version 3.5.171 of `BioGRID`. This database contains a compilation of protein-to-protein interactions and genetic interaction data for about sixty-one species. `BioGRID` includes interactions of artificially induced trans-species. All non-human interactions, including interactions that had human proteins/genes that interacted with proteins/genes of another species, were filtered out. The protein-to-protein interactions are considered physical interactions whilst the genetic interactions may refer to both physical and genetic interactions. Not all interactions had the physical/genetic label but all

interactions had an experiment type, hence it was possible to deduce the interaction type from the experiment in such cases. Examples of physical interaction experiments include affinity capture-luminescence, affinity capture-MS, co-crystal structure, FRET, and two-hybrid. Examples of genetic interaction experiments are dosage growth defect, dosage lethality, and dosage rescue. All the filtering and curation was done by means of a `Perl` script that recovered for the whole network ~~17,788~~15,139 nodes and ~~676,408~~623,659 ~~non-redundant~~ interactions from `BioGRID` (see Supplementary Tables 2, 3, and 4, for a comparison of the contribution made by each source database integrated into `RPGeNet` graph). The database, however, is filled with many undirected interactions. When direction of interaction is unknown or otherwise unstated, we assume bidirectionality. Our interactions graph building program took interactions with unknown direction and duplicate the interaction in the reverse direction (A⇌B will become A→B and B→A). For genetic interactions, the interactions were assumed to be unidirectional.

`STRING`: (5) The network was updated with interactions from version 11.0 of `STRING`. The top five sources for `STRING` were `GRID`, `INTACT`, `KEGG`, `BIOCARTA` and `REACTOME` (Supplementary Figure 1). Not all the interactions from this database have experimental evidence to back them up and many interactions are predictions of possible interactions. Because of this, any interaction not supported by evidence were discarded when building the `RPGeNet` core network. `STRING` database includes tags stating the directionality of the interaction; in the case that there is an interaction where the direction is not known, the interaction is assumed to be bidirectional. It also includes a large list of non-human protein interactions; those were filtered out as well. After the processing steps, ~~13,791~~13,269 nodes and ~~640,903~~629,271 ~~non-redundant~~ interactions were included from this database (further details on Supplementary Tables 2, 3, and 4).

`PPaxe`: (6) This text-mining tool can sift through academic papers to find interactions for the user's gene(s) of interest. `PPaxe` uses the random forest classifier algorithm, which is a machine learning method by which large collection of decorrelated decision trees are computed. `PPaxe` uses decision trees that combine different variables about the sentences it reads. One such variable that `PPaxe` considers is whether a verb describes the act of interaction or relationship. `PPaxe` was used to gather further interactions from scientific literature that were described and detected in published articles referred from `PubMed`. `PPaxe` replaces the `sparser` tool applied on the first release of `RPGeNet` with

the added benefit of using machine learning to gather a larger number of interactions than possible by hand. PPaxe can work on abstracts and full text articles; the first option gathers interactions solely from the abstract, but can process more articles because abstracts are generally free to read; the second option looks for interactions from entire articles, which implies a smaller set of articles. Each option was used in two separate searches: the first search was built to process PubMed papers that contained any of the 276 driver genes (65,820 abstracts and 29,819 full papers retrieved); the second search was scanning any interaction related to retinitis pigmentosa (1,124 abstracts and 502 full papers retrieved). All four PPaxe outputs were combined into one set and were then filtered by score and by the putative gene/proteins found (Supplementary Figure 2). Any interactions not having a gene identifier on the HGNC official nomenclature database (3) were filtered out using a Perl script. PPaxe does not infer yet directionality on retrieved interactions, so that bidirectionality is assumed. ~~2,376~~3,062 nodes and ~~7,862~~13,584 ~~non-redundant~~ interactions were collected for the RPGeNet core network (further details on Supplementary Tables 2, 3, and 4).

Database Manager

In order to handle a larger driver gene skeleton network and an increased amount of interactions, as well as to facilitate new ways of querying the data elements for the web interface, we had to resort to a more suitable database manager. Neo4j (community 3.1.7) was chosen for that purpose because it is a graph-based database manager that uses the property graph model to store and access the network data efficiently using a set of graph function instead of simply storing information in tables, like those used in traditional relational database managers as MySQL, etc (7). This database manager is used by other interaction database web applications, such as REACTOME and PlanNET, for storing and managing large interaction data (8,9). The property graph model uses nodes (the elements to store attributes/data of an entity) and relationships (relevant connections between nodes). Its native use of graph functions to query graph data makes neo4j an ideal system to store and manage information for all the network levels of RPGeNet, speeding up searches either complex or taking larger numbers of nodes into account.

WEB SERVER IMPROVEMENTS

Queries and Performance

As mentioned in the tool description, RPGeNet now has three distinct queries available to help users in finding genes or pathways of interest. Previously, RPGeNet would break when trying to access interactions at higher subnetwork levels, but the new RPGeNet engine can now handle searching and visualizing interactions from genes in the highest level with respect to the gene interest. The new database manager not only optimizes the searches and access of higher levels, but also makes possible that the new queries implemented in the new RPGeNet web interface were feasible and can be done in a reasonable amount of time.

Data Management and Visualization

The current RPGeNet upgrade facilitates navigation through all data available, making it more accessible and producing more informative results. Cytoscape.js (10) was used to display the interactive graphs in RPGeNet (see Figure 3). The interactions between genes are now colour coded depending on what type of interaction exists between them (in blue for genetic, red for physical or black for unknown edges, respectively). If multiple interaction types exist between two genes, multiple arrows with the corresponding interaction type colour will be drawn between the two genes. The driver genes also have distinctive shapes depending on whether they are associated with syndromic or non-syndromic retinitis pigmentosa, which is particularly useful for genetic diagnosis. Finally, clicking on a gene of interest provides users with further information about it from a pop-up panel like the one shown in Figure 5 (left panel).. A basic summary of the gene is given: all of the known aliases, related expression data, functional annotation in Gene Ontology (GO), the subnetwork level at which the gene is found within the RPGeNet network, the number of known variants, and external links to its GeneCards, UniProt, OMIM, and RetNet pages to access further information if needed. When users click on a given interaction, a complete information panel is also provided that summarizes all the evidences supporting that edge as well as links to the external references if available (see Figure 5, right panel)

Gene Expression Layer

RPGeNet continues to use the NCBI GEO (11) entry GSE7905 (12) as an example of expression data that can be projected into the network. For this expression set there are several precomputed analyses available, like retina only absolute expression, retina fold-change versus all other tissues, and fold-change with respect to liver on the same microarray experiment. However, the new implementation facilitates the integration of further expression datasets, some of them are already in progress and we expect to make them available soon. Another improvement made on the network explorer interface is that charging expression data on the current visualized network can be done on the fly, without having to repeat the query as it happened in the previous version.

Other Improvements on the Web Interface

RPGeNet network explorer now has an “undo” and a “redo” buttons, recording up to five changes made to the graph. The “undo/redo” buttons facilitate exploring the network with the already available “add/remove” buttons too. Another add-on to the visualization interface is a “search” bar that can look for any gene(s) and highlight them in the displayed graph; which is especially helpful when working on large graphs in the network explorer. There were also improvements made to the “save image” and “save graph” buttons to reduce the number of steps required; now the user is asked for the saving directory directly. One main improvement has been introduced to the “save graph” button, which was initially only saving the nodes identity but not the nodes distinct locations within the graph so, when reuploading the graph to RPGeNet, the nodes were not necessarily laid out in the same way as in the previous session. When importing a graph, now the nodes are laid out exactly as in the previous session, facilitating the storage of manual rearrangements made by users across different work sessions.

DISCUSSION

Using open-source databases, like BioGRID and STRING, has the advantage that they are free and commonly used within the scientific community. The problem with these large databases is that they are usually too large to serve the community for specific necessities and need to be further curated by researchers to distil the relevant biological network data from noise. BioGRID, has experimental evidence backing every interaction in their database. STRING, on the other hand, has

many predicted interactions, which can be a good start for researchers interested in finding novel evidences for them. STRING and BioGRID share many of the same interactions, although the raw STRING database does have more interactions due to the predictions and the larger number of species in comparison to BioGRID. Despite STRING not having all of their interactions experimentally backed up, they do offer a much wider range of information for each interaction than does BioGRID. Once the STRING dataset was processed, all interactions that did not have experimental evidence were removed. Since the interactions without evidences were excluded, the new RPGeNet has a smaller whole graph than the previous version of RPGeNet that was also considering the predictions. We now have ~~19,416~~18,542 nodes and ~~1,210,705~~1,218,032 edges~~—~~defining 613,319 non-redundant interactions~~—~~ versus the 63,139 nodes and 1,688,656 edges in the core network of the past version of RPGeNet.

On the other hand, using the PPaxe machine learning software, we managed to find multiple interactions, but still required some post-filtering to ensure that all the interactions found were indeed protein/genetic interactions. PPaxe cannot distinguish yet between a genetic and a protein interaction, so all PPaxe derived interactions in the network were labelled as "unknown" interactions (and coloured in black to distinguish from the other interactions). PPaxe retrieves the PubMed ID (PMID) of the article from which the interaction was found; those PMIDs are now available on RPGeNet, so that those who may be interested can figure out whether the “unknown” interaction of interest describes a protein or a genetic interaction by jumping to the corresponding PubMed entry. Regardless of the cons, PPaxe is simpler to use and is able to retrieve interactions without defining any syntactic pattern, unlike the previous sparser method.

In relation to the updated core whole network, shortest paths between two retinitis pigmentosa driver genes had distances from one to seven, where a distance of one means that there is a direct interaction between two driver genes and any number above one is the number of genes in between the two driver genes that made up the shortest pathway. The shortest paths distances for all 276 driver genes fall within three to four edges; in other words there are two to three connecting genes between them (Figure ~~5~~6). The subnetworks can be compared by the topology of each of the level’s own graph (see Supplementary Table 1). The average degree is 17.68~~8.344~~ in the skeleton (~8.84 in-/out-degree), 104.458~~48.894~~ in level one (~52.23 in-/out-), and 131.49~~62.371~~ in level three (~65.74

in-/out-). The large increase of average degree for level one mainly results from both adding new nodes and basically much more interactions; obviously, network saturates faster at nodes than at interactions (about 4.44 fold5% and 26.24 fold increase from skeleton,30% respectively, but accounting for 96.27% of nodes and 76.54% of edges from wholegraphincreasing about 5% and 30% respectively). Such trend can be observed on the corresponding in-/out-degree density graphs (see Supplementary Figure 3), as well as on the aforementioned Supplementary Table 1).

With an increased number of driver genes, it was expected for the RPGeNet core whole network to swell immensely. Previously, the network saturated at subnetworks of level four meaning that there were no more interactions within the network above that level. The only genes not found within these four subnetwork levels were genes that were unconnected to the rest of the network. The new RPGeNet network saturates earlier at level three. There is a decrease in the number of total nodes and interactions in relation to the previous version, but there is also a large number of supporting evidences for interactions that did not exist in the previous network. Most of the nodes and interactions no longer included are the result of stricter filtering of the data from the STRING database and improved anti-aliasing of node identifiers over HGNC standard symbols; only interactions with evidence were added to the network and predictions were left out. It is possible that these new nodes and interactions filled up missing gaps in the network, sketching new pathways to be discovered.

Even with an earlier saturation at level three, there were still driver genes that did not connect to any other member of the network. Some of the unconnected driver genes within the network were mitochondrial genes, like *MT-ND4*, *MT-TP*, *MT-TS2*. This may be because there is not enough research on interactions between mitochondrial genes/proteins and autosomal genes/proteins despite the fact that clear genetic communication between the nucleus and the mitochondria is known (13) and that most proteins of the mitochondria are, in fact, encoded in the autosomal DNA (14). Yet the proteins that are encoded in mitochondrial DNA are all important for the electron transport chain and connect well with each other (15,16). There were also few autosomal genes that did not connect to the network but that may simply be because they do not have any known interactions at the moment or they have not been characterized at an experimental level in depth (see Supplementary Tables 2 5 and 3 6 for a list of driver and non-driver genes, respectively, not connected to the core interactions network).

The RPGeNet network was curated by reducing the network to the shortest paths between known driver genes, allowing users to better identify genes and pathways important in the development of retinitis pigmentosa. Using RPGeNet many potential candidate genes have been identified by inspecting the shortest paths found in the skeleton graph. One of the candidate genes identified in the skeleton, *SIRT1*, has recently been experimentally confirmed to interact with *CERKL*. More importantly, it was found that *CERKL* regulates autophagy via *SIRT1* (17). This discovery supports *SIRT1* as a new driver gene of retinitis pigmentosa and confirms the utility of the RPGeNet model to identify potential IRD candidate genes. Furthermore, RPGeNet allows to visualize and to highlight new connections even in known interaction networks. The subnetwork retrieved after querying for three retinal-specific transcription factors (shortest path between *NRL* and *NR2E3*, plus addition of *CRX*), allows showing ~~to easily show~~ their connection to other causative retinal dystrophy genes (Figure ~~6~~7A). In addition, such subnetwork can be easily trimmed~~,~~ by omitting "noisy" nodes to focus on particular interactors. In this case, deletion of the nodes unrelated to transcriptional regulation and chromatin remodelers unveils new regulatory loops between these transcription factors that may be relevant for retinal development and maintenance (Figure ~~6B~~7B).

There are plans on the way to create a mouse and zebrafish RPGeNet specific interaction networks, as they are the two most used model organisms for research on retinal dystrophies, and later on to integrate them with the human network currently available. The newer RPGeNet graph engine will facilitate clustering gene nodes against a separate network layer based on disease nodes; for instance, as described in Lázaro-Guevara *et al* (18). New retinal differential gene-expression data from new RNA-seq and proteomic experiments is under analysis and will be added soon. We are also working on an automated pipeline to automate the protocol used to create RPGeNet, so it would be easier to keep it up-to-date as well as to expand the procedure to generate specific interaction networks for other rare diseases.

AVAILABILITY

RPGeNet is an open-source refined interaction network for retinitis pigmentosa. The RPGeNet website provides data description and a complete tutorial. Visit RPGeNet at https://compgen.bio.ub.edu/RPGeNet

SUPPLEMENTARY DATA

Supplementary Data are available at “Database” online.

ACKNOWLEDGEMENTS

This work was supported by research grants from BFU2017-83755P (Spanish Ministry of Economy), and 2017-SGR-1455 (Generalitat de Catalunya) to JFA; and SAF2016-80937-R (Ministerio de Economía y Competitividad/FEDER), 2017 SGR 738 (Generalitat de Catalunya), and La Marató TV3 (Project Marató 201417-30-31-32) to GM and RGD. S.C.-L. is a fellow of the Catalan Government ‘AGAUR’ (FI- FDR, 2017FI_B_00191). V.T. is fellow of the MINECO (BES-2014-068639, Ministerio de Economía, Industria y Competitividad).

CONFLICT OF INTEREST

G.M. and R.G-D. are co-founders and assessors of DBGen, a spin-off of the Universitat de Barcelona dedicated to the genetic diagnosis of visual disorders.

The authors declare there is not a competing interest.

REFERENCES

1. Daiger, S. P.; Sullivan, L. S. and Bowne, S. J. RetNet, the Retinal Information Network https://sph.uth.edu/retnet/
2. Boloc, D.; Castillo-Lara, S.; Marfany, G.; Gonzàlez-Duarte, R. and Abril, J. F. (2015) Distilling a Visual Network of Retinitis Pigmentosa Gene-Protein Interactions to Uncover New Disease Candidates, *PLoS One*, **10**, e0135307. PMID:25952370
3. Braschi, B.; Denny, P.; Gray, K.; Jones, T.; Seal, R.; Tweedie, S.; Yates, B. and Bruford, E. (2019) Genenames.org: the HGNC and VGNC resources in 2019., *Nucleic Acids Res.*, **47**, D786–D792. PMID:30304474
4. Chatr-Aryamontri, A.; Oughtred, R.; Boucher, L.; Rust, J.; Chang, C.; Kolas, N. K.; O'Donnell, L.; Oster, S.; Theesfeld, C.; Sellam, A.; Stark, C.; Breitkreutz, B. J.; Dolinski, K. and Tyers, M. (2017) The BioGRID interaction database: 2017 update, *Nucleic Acids Res.*, **45**, D369–D379. PMID:27980099
5. Szklarczyk, D.; Morris, J. H.; Cook, H.; Kuhn, M.; Wyder, S.; Simonovic, M.; Santos, A.; Doncheva, N. T.; Roth, A.; Bork, P.; Jensen, L. J. and von Mering, C. (2017) The STRING database in 2017: quality-controlled protein-protein association networks, made broadly accessible., *Nucleic Acids Res.*, **45**, D362–D368. PMID:27924014
6. Castillo-Lara, S. and Abril, J. F. (2018) PPaxe: easy extraction of protein occurrence and interactions from the scientific literature., *Bioinformatics*, bty988 [Epub ahead of print]. PMID:30500875
7. Robinson, I.; Webber, J. and Eifrem, E. *Graph Databases: New opportunities for connected data.*; O'Reilly Media, Inc., 2014
8. Fabregat, A.; Jupe, S.; Matthews, L.; Sidiropoulos, K.; Gillespie, M.; Garapati, P.; Haw, R.; Jassal, B.; Korninger, F.; May, B.; Milacic, M.; Roca, C. D.; Rothfels, K.; Sevilla, C.; Shamovsky, V.; Shorser, S.; Varusai, T.; Viteri, G.; Weiser, J.; Wu, G.; Stein, L.; Hermjakob, H. and D'Eustachio, P. (2018) The Reactome Pathway Knowledgebase, *Nucleic Acids Res.*, **46**, D649–D655. PMID:29145629
9. Castillo-Lara, S. and Abril, J. F. (2018) PlanNET: homology-based predicted interactome for multiple planarian transcriptomes., *Bioinformatics*, **34**, 1016–1023. PMID:29186384
10. Franz, M.; Lopes, C. T.; Huck, G.; Dong, Y.; Sumer, O. and Bader, G. D. (2016) Cytoscape.js: a graph theory library for visualisation and analysis., *Bioinformatics*, **32**, 309–11. PMID:26415722
11. Barrett, T.; Wilhite, S. E.; Ledoux, P.; Evangelista, C.; Kim, I. F.; Tomashevsky, M.; Marshall, K. A.; Phillippy, K. H.; Sherman, P. M.; Holko, M.; Yefanov, A.; Lee, H.; Zhang, N.; Robertson, C. L.; Serova, N.; Davis, S. and Soboleva, A. (2013) NCBI GEO: Archive for functional genomics data sets - Update, *Nucleic Acids Res.*, **41** PMID:21097893
12. Dezső, Z.; Nikolsky, Y.; Sviridov, E.; Shi, W.; Serebriyskaya, T.; Dosymbekov, D.; Bugrim, A.; Rakhmatulin, E.; Brennan, R. J.; Guryanov, A.; Li, K.; Blake, J.; Samaha, R. R. and Nikolskaya, T. (2008) A comprehensive functional analysis of tissue specificity of human gene

expression, *BMC Biol.*, **6**, 49. PMID:19014478

13. Brandvain, Y. and Wade, M. J. (2009) The Functional Transfer of Genes From the Mitochondria to the Nucleus: The Effects of Selection, Mutation, Population Size and Rate of Self-Fertilization, *Genetics*, **182**, 1129–1139. PMID:19448273
14. Berg, O. G. and Kurland, C. G. (2000) Why Mitochondrial Genes are Most Often Found in Nuclei, *Mol. Biol. Evol.*, **17**, 951–961. PMID:10833202
15. Anderson, S.; Bankier, A. T.; Barrell, B. G.; de Bruijn, M. H. L.; Coulson, A. R.; Drouin, J.; Eperon, I. C.; Nierlich, D. P.; Roe, B. A.; Sanger, F.; Schreier, P. H.; Smith, A. J. H.; Staden, R. and Young, I. G. (1981) Sequence and organization of the human mitochondrial genome, *Nature*, **290**, 457–465. PMID:7219534
16. SATOH, M. (1991) Organization of multiple nucleoids and DNA molecules in mitochondria of a human cell, *Exp. Cell Res.*, **196**, 137–140. PMID:1715276
17. Hu, X.; Lu, Z.; Yu, S.; Reilly, J.; Liu, F.; Jia, D.; Qin, Y.; Han, S.; Liu, X.; Qu, Z.; Lv, Y.; Li, J.; Huang, Y.; Jiang, T.; Jia, H.; Wang, Q.; Liu, J.; Shu, X.; Tang, Z. and Liu, M. (2019) *CERKL* regulates autophagy via the NAD-dependent deacetylase *SIRT1*, *Autophagy*, **15**, 453–465. PMID:30205735
18. Lázaro-Guevara, J. M.; Flores-Robles, B. J.; Garrido, K.; Pinillos-Aransay, V.; Elena-Ibáñez, A.; Merino-Meléndez, L.; López-Martínez, J. A. and Victoriano-Lacalle, R. (2018) Gene's hubs in retinal diseases: A retinal disease network, *Heliyon*, **4**, e00867. PMID:30417144
19. Krzywinski, M.; Schein, J.; Birol, I.; Connors, J.; Gascoyne, R.; Horsman, D.; Jones, S. J. and Marra, M. A. (2009) `Circos`: An information aesthetic for comparative genomics, *Genome Res.*, **19**, 1639–1645. PMID:19541911

TABLES AND FIGURES

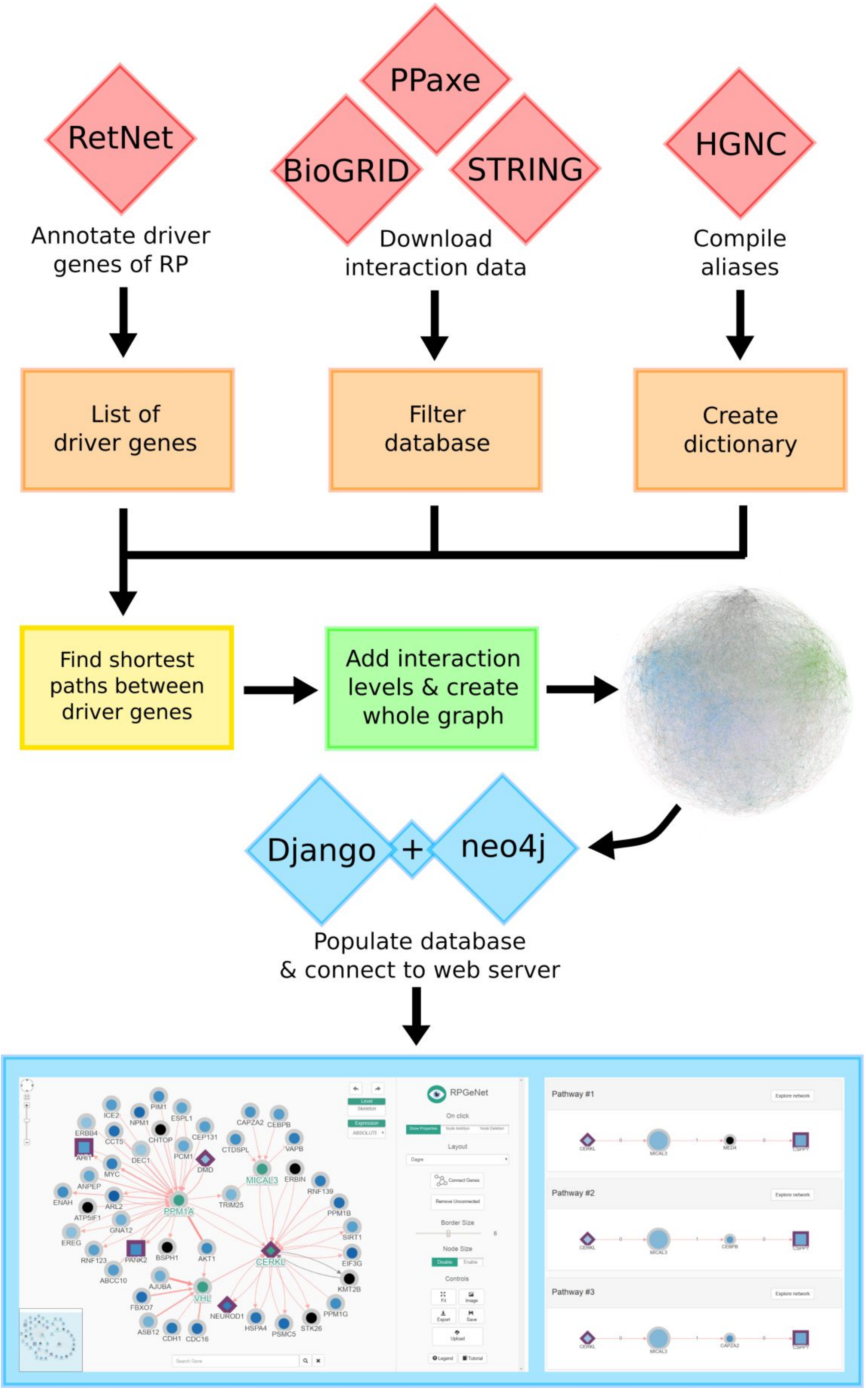

Figure 1.- Data integration workflow to build the `RPGeNet` core database.

The final graphical web interface (bottom panels) depends on a series of data integration steps that provide the interactions and nodes to the main `neo4j` database engine. Each component of the workflow is described in detail on the main text.

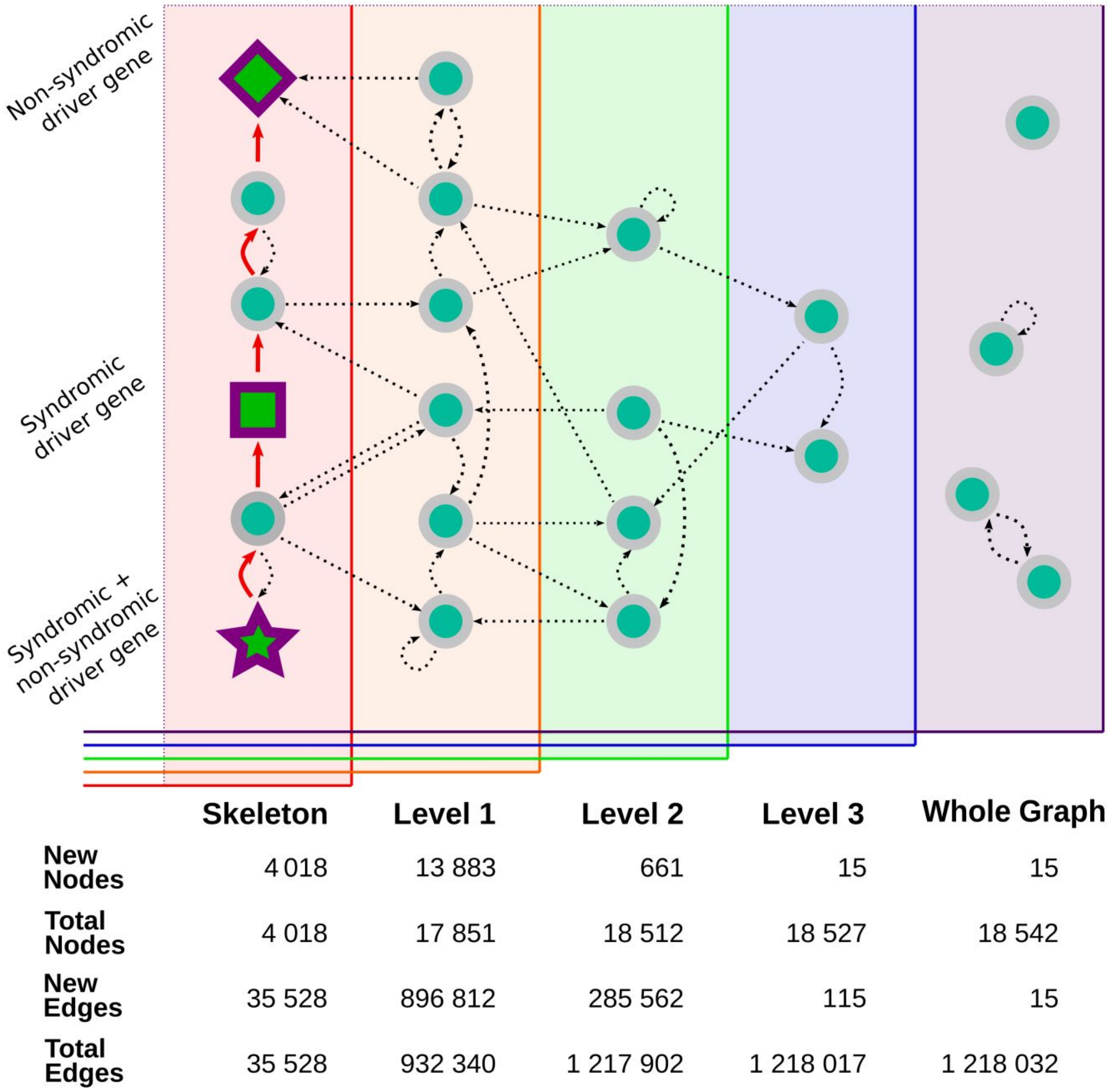

	Skeleton	Level 1	Level 2	Level 3	Whole Graph
**New Nodes**	4 018	13 883	661	15	15
**Total Nodes**	4 018	17 851	18 512	18 527	18 542
**New Edges**	35 528	896 812	285 562	115	15
**Total Edges**	35 528	932 340	1 217 902	1 218 017	1 218 032

Figure 2.- Visual representation of the expansion of the core interactions graph.

The graph builder begins with the construction of the skeleton graph, represented by all the nodes on the leftmost panel. The skeleton is created by finding the shortest direct interaction paths (the red arrows) between all the known driver genes—drawn here using the same shapes as in `RPGeNet Network Explorer` (star, square, and diamond shapes, based on whether their mutations cause or not syndromic diseases)—. The graph is then expanded into level 1 (represented as the orange panel) by adding all of the parents and children (straight lines) of the nodes already found in the skeleton graph. Level-specific interactions are shown as curved connections linking nodes within a given graph level. The expansion is repeated until the highest level is reached and all known genes with known interactions have been connected to the interactions core graph. The remaining genes that do not have any known interaction that connects them with the core graph are included in the whole graph level. Some of those genes may have interactions with other genes found only in the whole graph level (and many of those interactions are self-references). The bottom table from this figure compares the number of nodes and edges added on each level ("New Nodes" and "New Edges" rows), as well as the total number of nodes and edges accumulated ~~on each level~~.

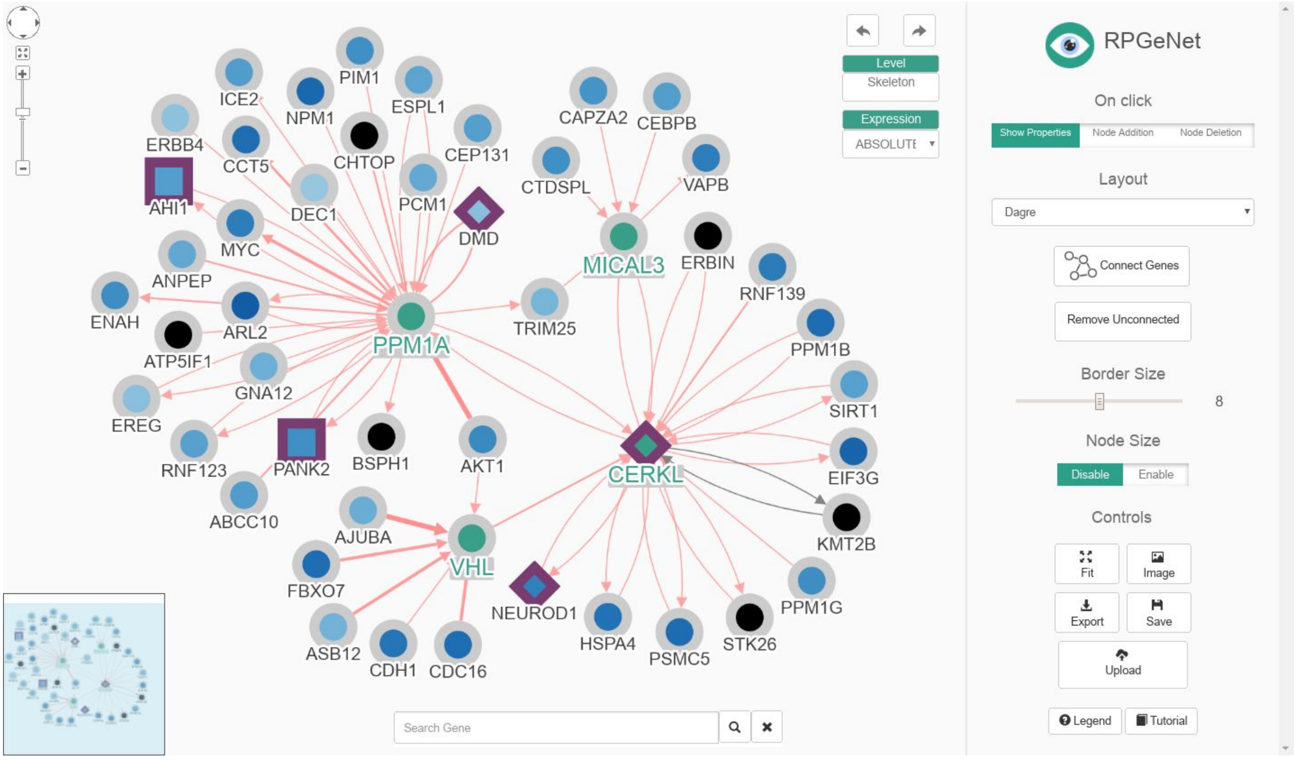

Figure 3.- The renewed `Network Explorer` interface of `RPGeNet`.

In this example, the `Network Explorer` interface shows a subgraph containing all the genes that directly interact with *CERKL* within the skeleton subnetwork, after expanding nodes for ~~*PPM1B*~~ *PPM1A, VHL,* and *MICAL3*~~.~~ (those selected four nodes highlighted in green, driver genes border in purple, node colors based on the "ABSOLUTE" gene-expression data). Some improvements to the interface can be appreciated: a "search" gene add-on at the mid-bottom, an expression data set selection drop-down menu at right-top corner of the network visualization canvas, as well as the "`undo`"/"`redo`" buttons. A more dynamic "buttons panel" on the right facilitates the interaction with the network data. Finally, the coloured-by-type interactions also provide directionality information with arrow heads, and have reliability-score proportional widths adjusted to the number of evidences supporting them. This figure can be reproduced on the Network Explorer if users upload the Supplementary File 1.

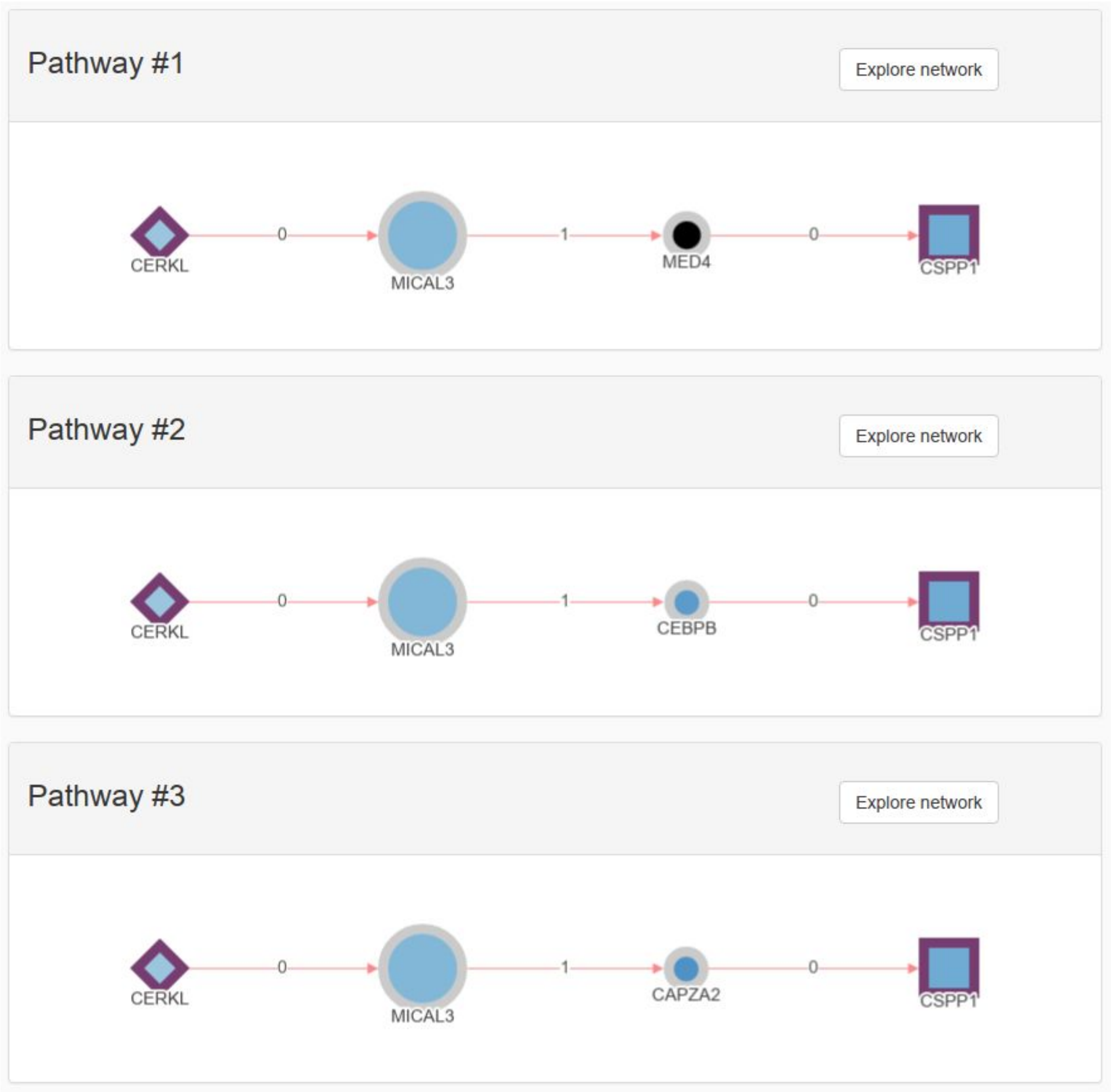

Figure 4.- Example of a pathways list returned using the shortest pathways `RPGeNet` query. *CERKL* and *CSPP1* where used to start the pathway search on the main `RPGeNet` form. Only ~~T~~the first three pathways of the 28 retrieved by that query are shown on this figure, all of them at the shortest path length of three (3 edges and 2 nodes between the chosen identifiers). ~~b~~By clicking on the corresponding “Explore Network” button on any of the listed pathways, users can easily jump to the Network Explorer interface to work on the selected genes for that pathway.

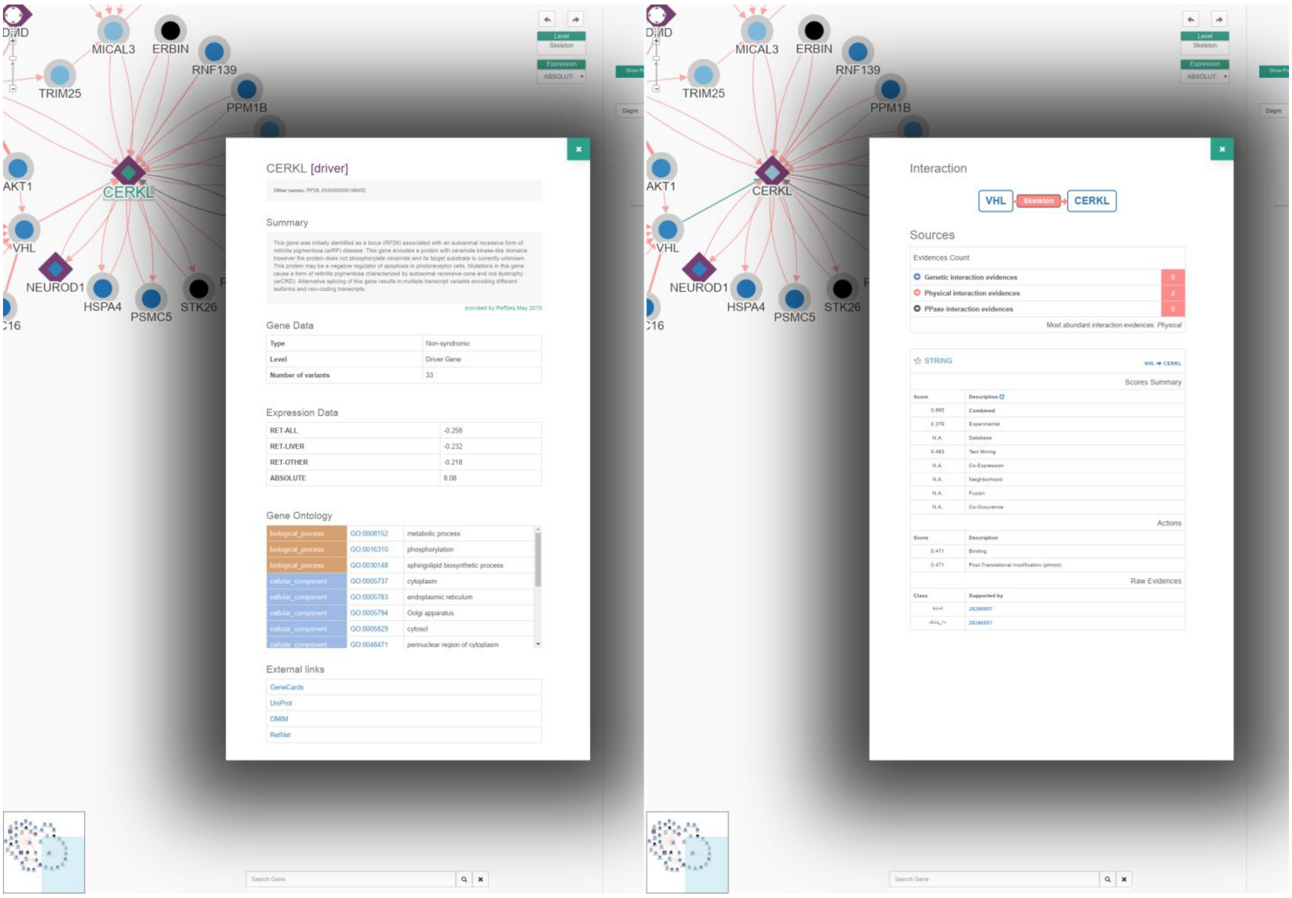

Figure 5.- An example of node (left) and interaction (right) information panels from the `Network Explorer` interface.

The default behaviour "*On click*" of the `Network Explorer` interface is to show "*node properties*" (see topmost controls on the right panel of that interface on previous figure). From the network example of Figure 3, when clicking at the *CERKL* node the gene/protein information panel pops up to display a description of the gene, known aliases, a summary of its expression levels and functional annotation, and links to external references. On the other hand, by clicking on an edge, *VHL* to *CERKL* in this example, the interaction panel pops up, providing information about the type of possible interactions (genetic [blue], physical [red], or "unknown" [black]), as well as a series of tables containing details about the supporting evidences from the distinct sources, along with the corresponding external links to the reference databases and to the supporting evidences when possible.

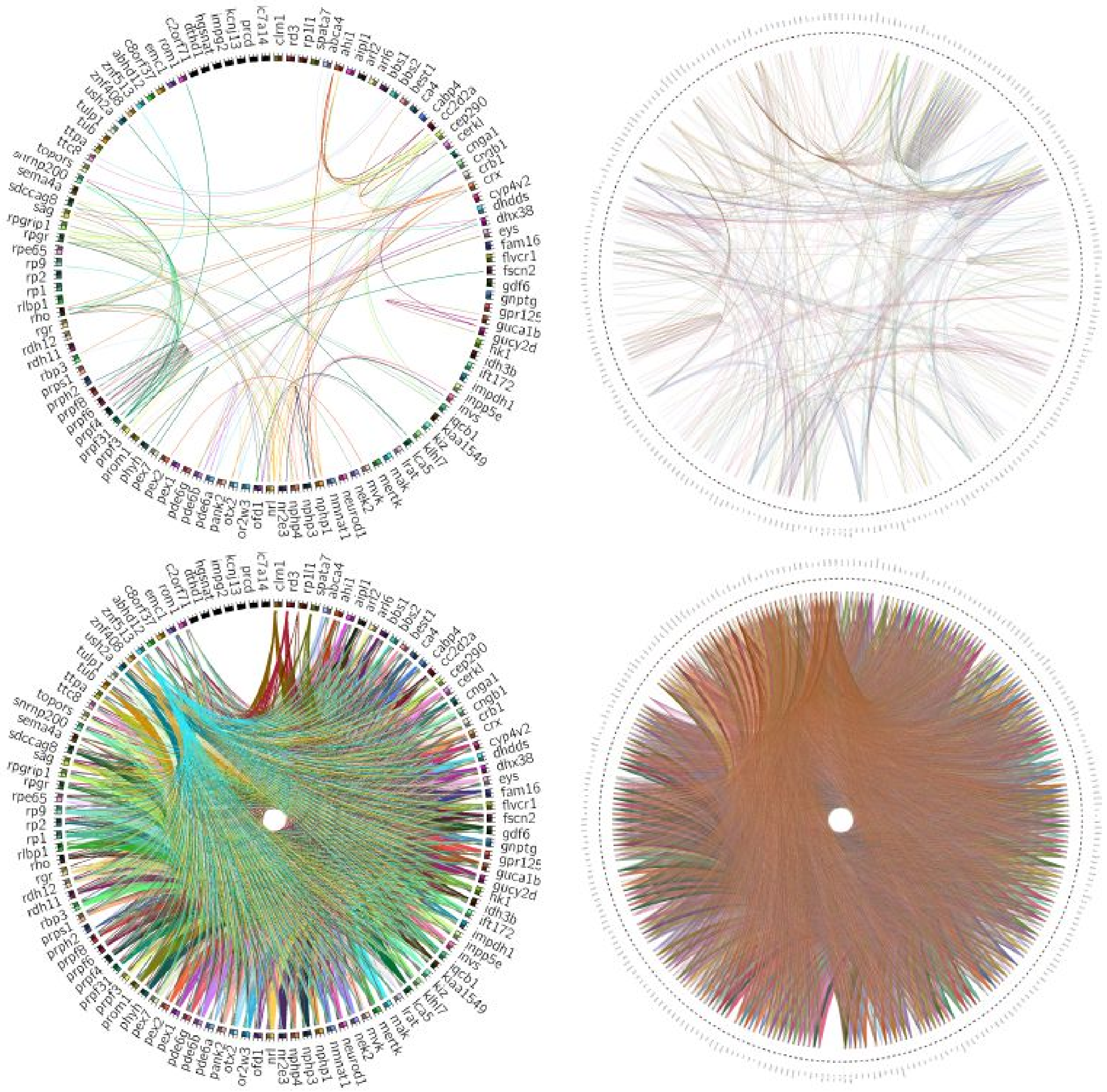

Figure ~~5~~6.- ~~Comparing~~ Analysis of `RPGeNet` v1 and v2 networks connectivity with `Circos` (19).

The figure compares the connectivity of the driver genes of the old `RPGeNet` v1.0 (left) and the updated `RPGeNet` v2.0 (right). The top ~~comparison~~ pair of plots~~panels~~ show all the shortest paths between driver genes at distance one, meaning direct interaction between each pair of driver genes. The bottom par of plots~~panels~~ provide the comparison at distance 3, meaning there are two genes in the shortest path between a pair of driver genes of interest. It is clear from the visualized `Circos` plots that the updated `RPGeNet` database has a highly connected network.

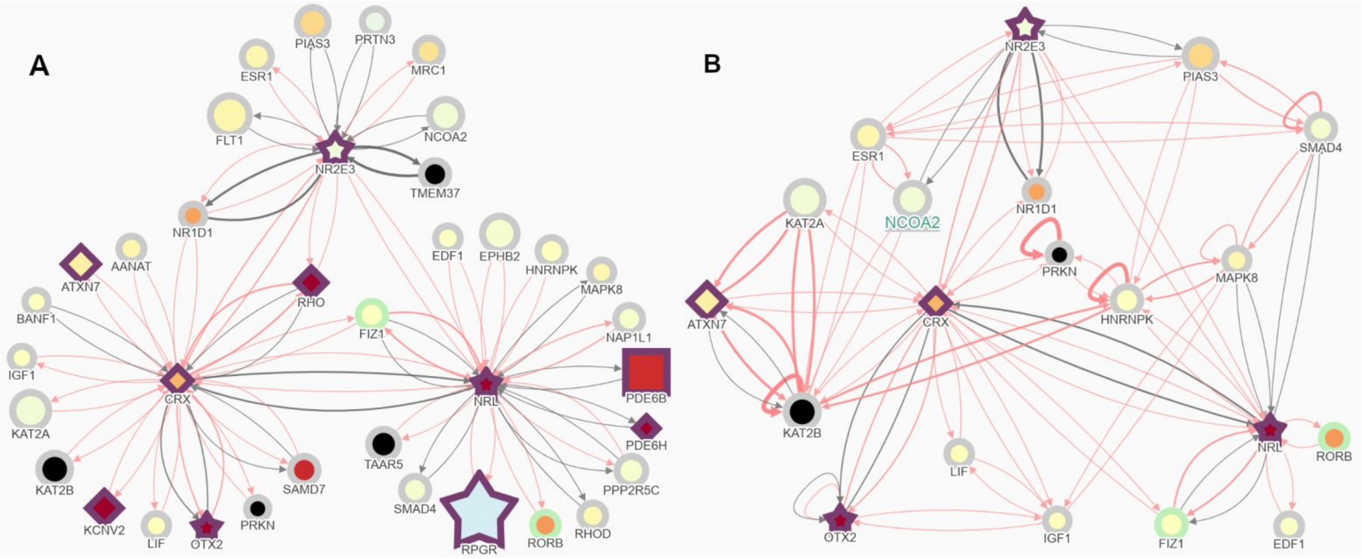

Figure ~~6~~7.- A control case interaction visualized on RPGeNet showing the connections between *NRL*, *NR2E3* and *CRX* retinal transcription factors.

A) RPGeNet was queried to display the subnetwork ~~for the shortest path between~~among *NRL* ~~and~~, *NR2E3*~~;~~ ~~that subnetwork was explored by adding *CRX*~~ for the nodes at distance one ~~and the nodes interacting with each of them (~~ at level 1~~)~~; then nodes connected to *CRX* were added with the “*Node Addition*” button activated on click. *CRX*, *NRL*, and *NR2E3* are three well-known transcription factors that co-regulate retinal-specific genes, among them *RHO*. Interestingly, several other genes that cause retinal dystrophies also appear in the network as target genes or other transcriptional regulators (border shown in purple). Nodes color fill defined by the “RET-ALL” gene-expression data.

B) Further trimming of the subnetwork obtained by omitting the nodes that are not chromatin remodelers or transcription factors provide an overview of relevant co-regulators of retinal genes. After deleting the corresponding nodes, further edges—out of the shortest paths that link the genes left—were shown by clicking on the “*Connect Genes*” button at the control panel. This visualization allows pinpointing and exploring ~~other factors~~ alternate pathways that connect the initial seeds~~,~~ ; for instance, ~~the path linking *NR2E3* to~~ *CRX* and *NRL* were already connected~~via *NR1D1*~~ , but longer paths are now evident like *CRX*⇌*PRKN*⇌*HNRNPK*⇌*NRL*, when *PRKN* and *HNRNPK* are linked. Another example can be the pathway found between *NRL* and *NR2E3* on the path *NRL*⇌*SMAD4*⇌*PIAS3*⇌*NR2E3*, when *SMAD4* and *PIAS3* are linked.~~and *EP300*, and the links between the three factors via *SNAPC1*, *SP1*, and *SMAD4*.~~ Both panels from this figure can be reproduced on the Network Explorer if users upload the Supplementary Files 2 and 3, respectively.

SUPPLEMENTARY MATERIAL

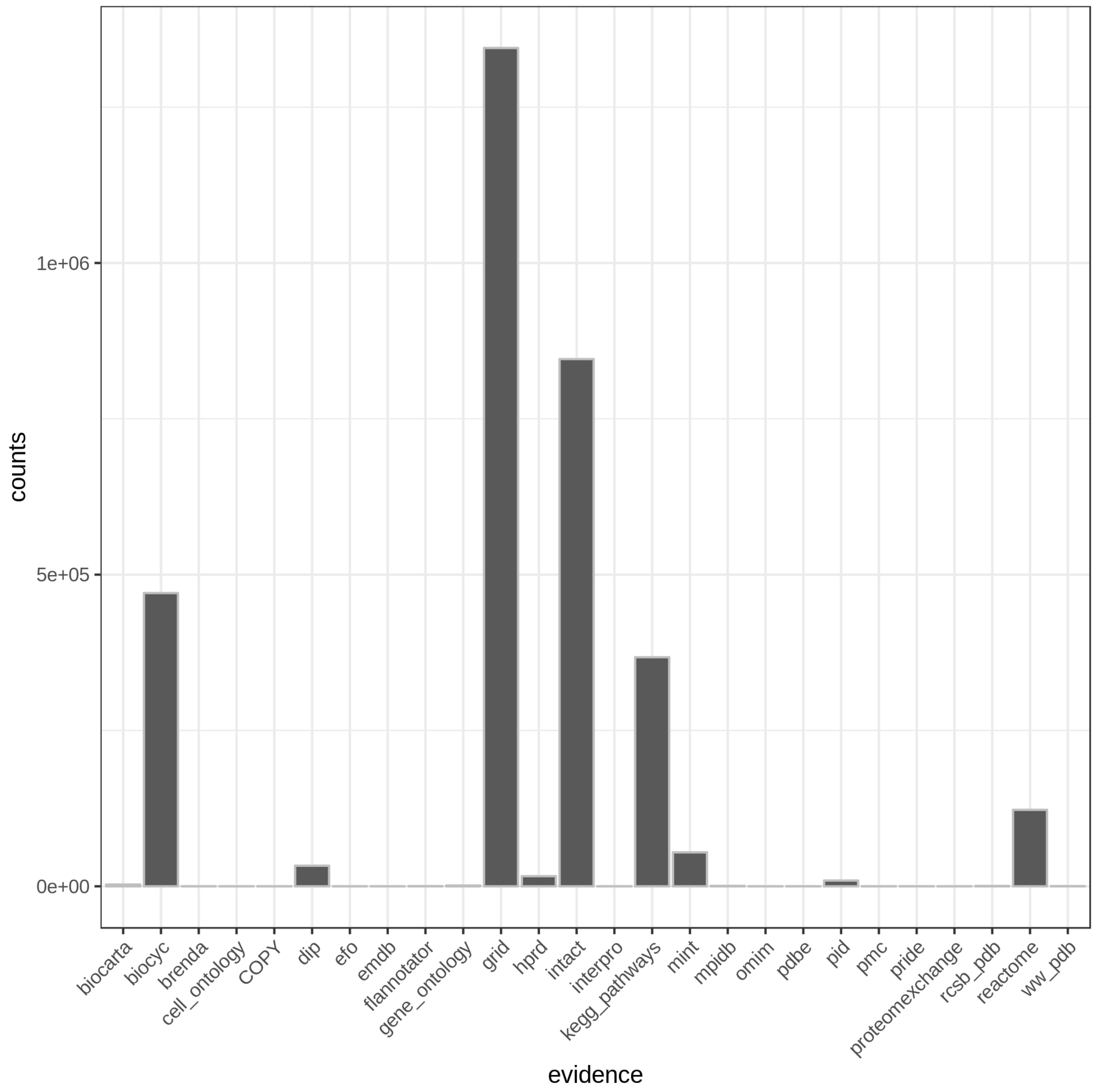

Supplementary Figure 1.- Bar plot with distribution of the different evidences used by `STRING`.
`STRING` is a protein-to-protein interactions database that includes predictions and experimentally validated interactions. The experimentally validated interactions include evidences from different sources. The proportion of such evidences is shown in this bar-plot. `GRID`, `INTACT`, `KEGG`, `BIOCARTA` and `REACTOME` are the most common sources of experimentally-validated interactions from `STRING`.

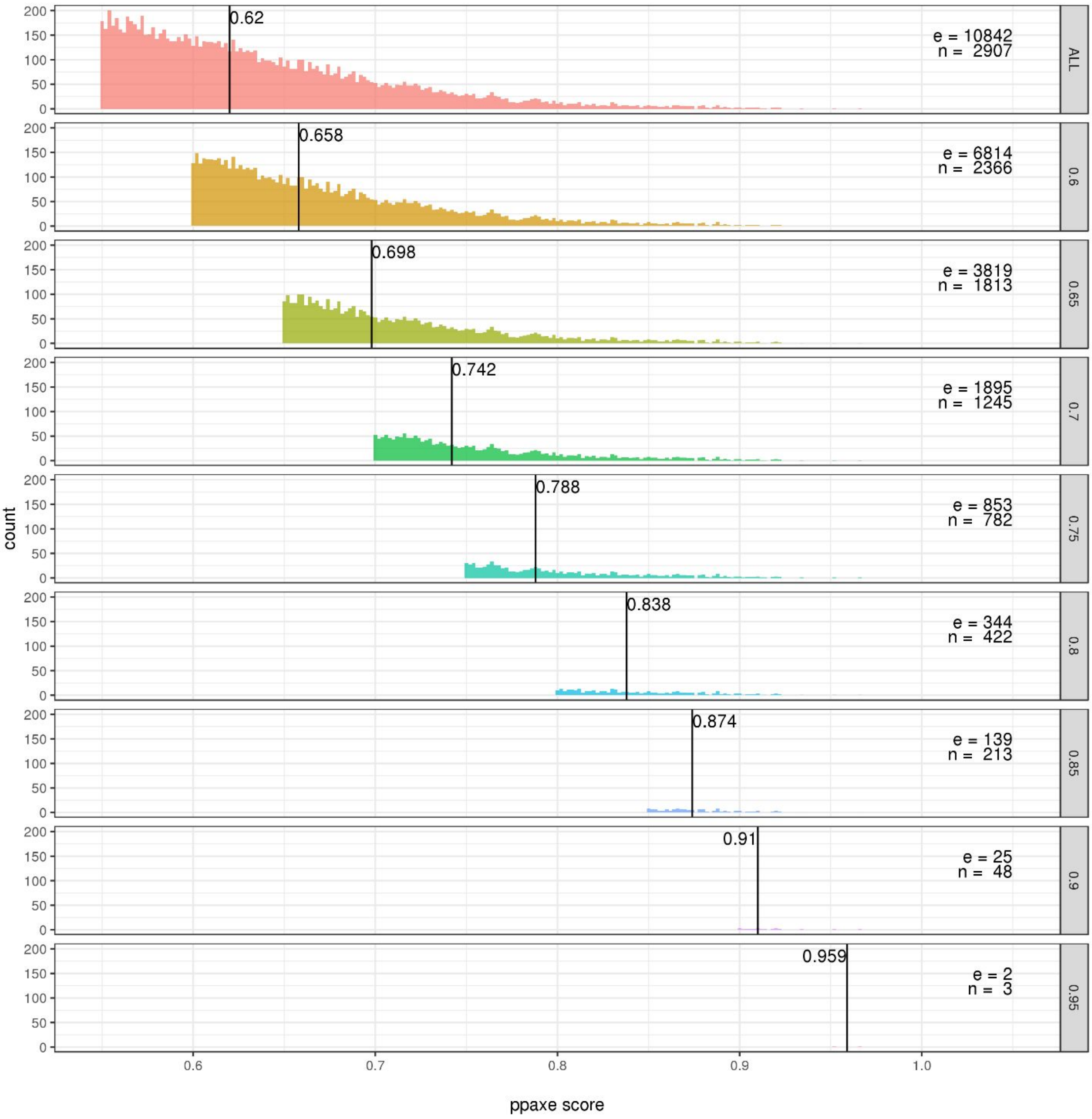

Supplementary Figure 2.- Choosing the optimal votes cut-off for `PPaxe`.

The graph shows the distribution of the un-normalized confidence score given to the interactions detected by `PPaxe` with respect to the number of `PPaxe` interactions (counts), and the average score depending on the cut-off score chosen (vertical line). In an attempt to optimize the number interactions while reducing false positive interactions and increasing false-negatives, the cut-off score chosen was 0.65, which was the value that yielded a minimum precision of 90% in the validation assessment performed in the original PPaxe manuscript (6). “e” and “n” correspond to the number of edges and nodes respectively that are passing the cut-off threshold. Panels categorize by the percentage of votes by the random-forest classifier.

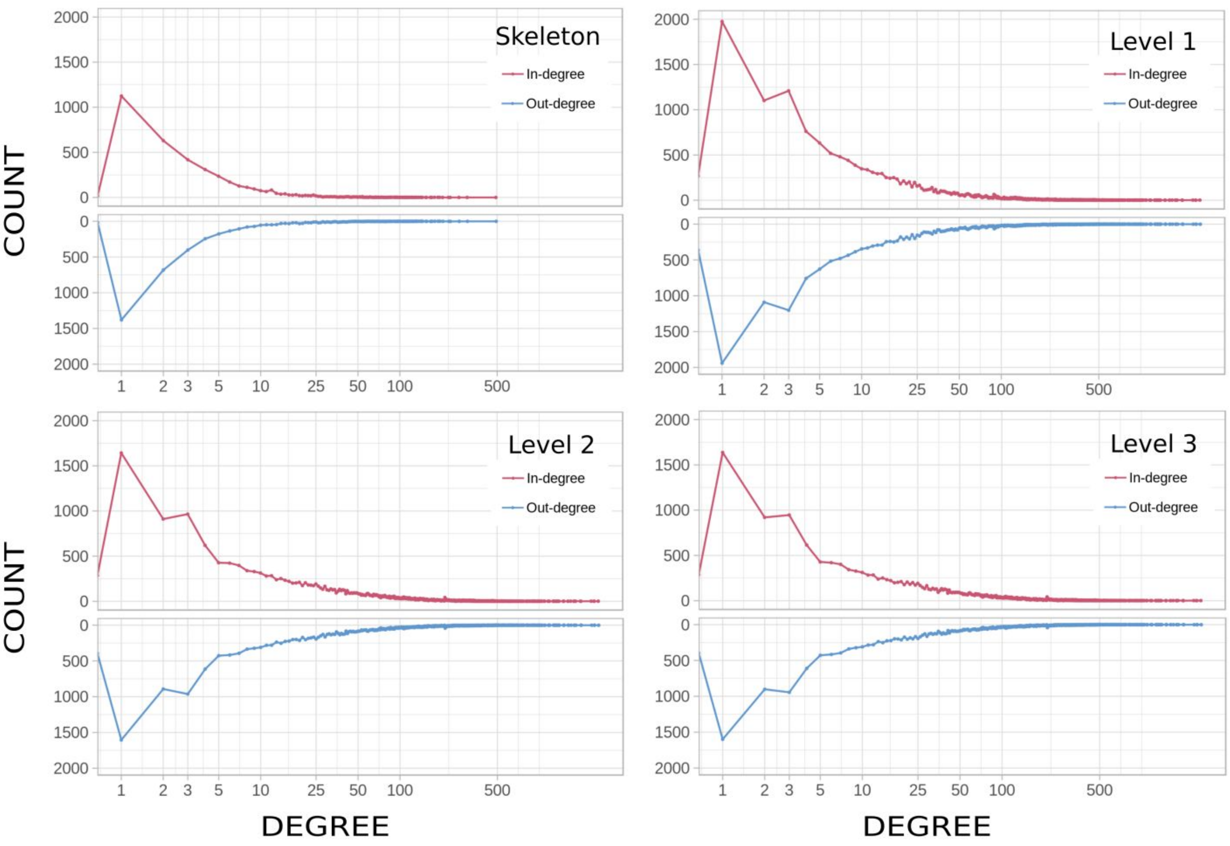

Supplementary Figure 3.- Comparing the number of counts between in and out degrees for each level of the RPGeNet core network.

In the skeleton graph the much smaller area between the in-/out-degree lines in comparison to higher level graphs denotes a smaller number of interactions. Comparing the skeleton with level one, there is a large increase in counts for lower degrees. However, as the graph is expanded to levels two and three, a decrease of counts in lower degrees can be observed. This can be explained by a larger jump in total number of interactions between skeleton and level one, followed by smaller increments in new interactions and a slower increase in degree from levels one to three, as new nodes and interactions are introduced.

GRAPH STATS	RPGeNet v1		RPGeNet v2 — All Sets					BioGRID Only		STRING Only		PPaxe Only	
	Skeleton	WholeGraph	Skeleton	Level1	Level2	Level3	WholeGraph	Skeleton	WholeGraph	Skeleton	WholeGraph	Skeleton	WholeGraph
**Total #Nodes**	1 294	22 372	4 018	17 851	18 512	18 527	18 542	3 057	15 205	2 555	13 325	762	3 146
**Isolated Nodes**	7	7	16	15	15	15	15	71	70	56	56	93	84
**Adjacent Drivers**	103 of **110**		260 of **276**	261 of **276**				205 of **276**	206 of **276**	220 of **276**		183 of **276**	192 of **276**
**Total #Edges**	5 883	752 062	35 528	932 340	1217 902	1 218 017	1 218 032	23 480	623 601	20 791	629 271	3 654	13 584
**Mutual** [A⇌B]	1 082	319 928	9 601	462 988	604 652	604 707	604 713	6 243	307 422	6 422	314 629	1 554	6 760
**Assymetric** [A→B]	3 719	106 907	16 326	5 074	5 931	5 931	5 931	10 994	6 138	7 947	0	546	0
**Self-loop** [A→A]	0	5 299	0	1 290	2 667	2 672	2 675	0	2 619	0	13	0	64
**Total Non-Redundant**	4 801	432 134	25 927	469 352	613 250	613 310	613 319	17 237	316 179	14 369	314 642	2 100	6 824
**Graph Density**	0.0035	0.0015	0.0022	0.0029	0.0036	0.0035	0.0035	0.0025	0.0027	0.0032	0.0035	0.0063	0.0014
**Avg. Clustering Coef.**	0.0433	0.1445	0.0613	0.1449	0.2331	0.2331	0.2331	0.0448	0.0737	0.1508	0.5512	0.0959	0.0580
**Graph Diameter**	10	9	8	6	7	8	8	8	7	9	10	13	13
**Graph Reciprocity**	0.3678	0.8568	0.5405	0.9946	0.9951	0.9951	0.9951	0.5318	0.9901	0.6178	1.0000	0.8506	1.0000
**Avg. Degree**	9.0927	67.2324	17.6844	104.458	131.5797	131.4856	131.3809	15.3615	82.0258	16.2748	94.4497	9.5906	8.6357
**Avg. Closeness**	0.0969	0.0185	0.052	0.0529	0.0529	0.0529	0.0295	0.0128	0.0116	0.0165	0.0052	0.0093	0.0024
**Betweenness**	3 977.16	32 888.11	10 423.13	33 430.86	34 988.77	35 073.29	35 044.91	7 551.86	28 684.70	6 868.09	31 567.57	1 635.56	7 714.01
**Avg. Edge Betw.**	1 151.99	1 480.75	1 629.48	980.37	812.19	814.28	814.27	1 362.32	1 064.77	1 144.27	942.69	463.38	2 354.06
**Avg. Coreness**	4.7751	39.8983	9.1309	54.8258	77.0557	77.0063	76.9456	7.9326	42.0278	8.7366	67.3668	5.1273	4.6300
**Avg. Eccentricity**	6.7272	4.5465	5.6904	4.5666	4.9552	5.8732	5.8691	5.5741	5.1786	6.2693	6.6453	6.8307	7.9259
**Avg. Path Length**	4.1559	2.9473	3.6155	2.8810	2.8969	2.9000	2.9000	3.5936	2.9143	3.8110	3.4375	3.7888	4.1479

Supplementary Table 1.- Topology of the `RPGeNet` interactions graph.

This table shows the total number of nodes (genes/proteins, where adjacent nodes can be calculated by substracting *isolated* from *total nodes*) and edges (interactions, including counts by the type of relation between pairs of adjacent nodes: mutual-, asymmetric- and self-interactions), for every level of the `RPGeNet` database graph (see "`RPGeNet v2` All Sets" block). For comparison purposes, graph stats are provided for the previous `RPGeNet v1` version, as well as those for the interaction networks that can be produced separately from each of the evidence sources (blocks named as "`BioGRID` Only", "`STRING` Only", and "`PPaxe` Only", respectively). Most of the *isolated nodes* correspond to drivers without interaction evidences from the selected sources, and those numbers complement the values from the *adjacent drivers* row. Graph statistics were described in depth on Newman *et al* [1]. In br: *graph density* is the ratio between edges and the total number of possible vertices (nodes); *average clustering coefficient* (or the tendency of nodes to cluster together) is a measure of how complete the neighborhood of a node is, over all the nodes of the network; *diameter* is the maximum distance between two nodes; *reciprocity* is the; *average degree* is the average number of edges per node; *closeness* is the average length of shortest path between a node and every other node in the network; *betweenness* is a measure of the number of times a node is found within the shortest paths between two other nodes; *coreness* defines the shell index of the vertices of a network; *eccentricity* is the maximum of the shortest distances of a node with respect all other nodes in the graph; finally, *average path length* is a measure of the average distance between two nodes. Graph stats produced with `python-igraph` library (v0.7.0, see further details at `https://igraph.org/python/`).

1. Newman MEJ, Barabási A-L, Watts DJ. The structure and dynamics of networks. 2006. p. 624

		Skeleton	Level1	Level2	Level3	WholeGraph
GRAPH SUMMARY	**Total UNIQUE NODES**	4 018	17 851	18 512	18 527	18 542
	Adjacent Nodes	4 002	17 836	18 497	18 512	18 527
	Isolated Nodes	16	15	15	15	15
	**NODES by Source**					
	BioGRID	3 677	14 831	15 132	15 136	15 139
	STRING	3 580	12 864	13 253	13 263	13 269
	PPaxe	1 407	3 016	3 054	3 056	3 062
	TOTAL	8 664	30 711	31 439	31 455	31 470
	Nodes Source "redundancy"	*215.63%*	*172.04%*	*169.83%*	*169.78%*	*169.72%*
	**Total DIRECTED EDGES**	35 528	932 340	1 217 902	1 218 017	1 218 032
	Mutual [A⇌B]	9 601	462 988	604 652	604 707	604 713
	Assymetric [A⇀B]	16 326	5 074	5 931	5 931	5 931
	Self-loop [A⇀A]	0	1 290	2 667	2 672	2 675
	Total Non-Redundant	25 927	469 352	613 250	613 310	613 319
	Directed Edges "redundancy"	*137.03%*	*198.64%*	*198.60%*	*198.60%*	*198.60%*
	**EDGES by Source**					
	BioGRID all	22 914	518 478	623 643	623 656	623 659
	BioGRID only	*21 334*	*483 667*	*579 207*	*579 220*	*579 220*
	STRING all	12 563	440 111	629 167	629 265	629 271
	STRING only	*10 599*	*402 920*	*582 094*	*582 190*	*582 190*
	PPaxe all	2 277	12 282	13 572	13 578	13 584
	PPaxe only	*1 534*	*8 049*	*8 984*	*8 988*	*8 988*
	TOTAL	37 754	970 871	1 266 382	1 266 499	1 266 514
	Edges Source "redundancy"	*145.62%*	*206.85%*	*206.50%*	*206.50%*	*206.50%*
EVIDENCE SUMMARY	**TOTAL EVIDENCES**	75 447	2 371 305	3 209 677	3 209 856	3 209 871
	**By Class**					
	Genetic evidences	257	6 018	7 062	7 063	7 063
	Avg. evids x directed edge	*0.007*	*0.006*	*0.006*	*0.006*	*0.006*
	Physical evidences	70 561	2 342 154	3 177 567	3 177 739	3 177 748
	Avg. evids x directed edge	*1.986*	*2.512*	*2.609*	*2.609*	*2.609*
	Unknown evids (PPaxe)	4 629	23 133	25 048	25 054	25 060
	Avg. evids x directed edge	*0.130*	*0.025*	*0.021*	*0.021*	*0.021*
	**By Source**					
	BioGRID	31 726	705 485	842 798	842 815	842 818
	Physical interactions	*31 469*	*699 467*	*835 736*	*835 752*	*835 755*
	Genetic Interactions	*257*	*6 018*	*7 062*	*7 063*	*7 063*
	Avg. evids x directed edge	*0.893*	*0.757*	*0.692*	*0.692*	*0.692*
	STRING	39 092	1 642 687	2 341 831	2 341 987	2 341 993
	Avg. evids x directed edge	*1.100*	*1.762*	*1.923*	*1.923*	*1.923*
	PPaxe	4 629	23 133	25 048	25 054	25 060
	Avg. evids x directed edge	*0.130*	*0.025*	*0.021*	*0.021*	*0.021*
EDGES with STRING SCORE	**With any STRING score**	**12 563**	**440 111**	**629 167**	**629 265**	**629 271**
	With "experimental" score	2 696	75 075	109 191	109 231	109 235
	With "database" score	9 112	371 666	541 032	541 082	541 084
	With "text-mining" score	8 803	248 133	345 610	345 682	345 686
	With "co-expression" score	2 227	107 246	157 974	158 008	158 010
	With "neighborhood" score	0	0	0	0	0
	With gene-"fusion" score	16	1 136	2 384	2 384	2 384
	With "co-occurrence" score	124	6 320	8 749	8 755	8 757

Supplementary Table 2.- Source origin and redundancy of evidences for `RPGeNet` core network interactions. First block accounts for the number of nodes and edges supported by each input source at each graph level of the core network. Second block provides information about distinct evidences used to weight the interactions. Number of interactions on the core network with `STRING` scores is provided on the last block to complement evidences (also shown in the interaction information pop-up cards like the one shown in Figure 5 right panel). `RPGeNet` models interactions as directed edges between nodes, and thus, represents undirected interactions between genes as two separate interactions. The "Total Interactions" column shows the total number of edges stored in the database (without taking into account the number of evidences), counting reciprocal interactions twice (A⇀B is different from B⇀A). The "Non-redundant Interactions" column refers to the number of interactions in `RPGeNet` independently of the direction, and thus, the pair A⇀B and B⇀A is only counted once. The Non-redundant interactions count criteria is equivalent to the one used by the `BioGRID` database (https://wiki.thebiogrid.org/doku.php/build_3.5.171).

	Skeleton		WholeGraph	
	Total # Nodes	Total # Edges	Total # Nodes	Total # Edges
**Run Together (`RPGeNet v2`)**	4 018	35 528	18 542	1 218 032
**Overlapping Sets**	4 767	46 610	18 539	1 217 974
**`BioGRID` only**	2 031	22 757	4 956	579 165
**`STRING` only**	1 389	19 650	2 598	582 196
**`PPaxe` only**	136	2 994	113	8 994
**`BioGRID` ∩ `STRING`**	585	549	7 839	43 029
**`BioGRID` ∩ `PPaxe`**	45	68	145	544
**`STRING` ∩ `PPaxe`**	185	486	623	3 183
**`BioGRID` ∩ `STRING` ∩ `PPaxe`**	396	106	2 265	863
**Total from `BioGRID`**	3 057	23 480	15 205	623 601
**Total from `STRING`**	2 555	20 791	13 325	629 271
**Total from `PPaxe`**	762	3 654	3 146	13 584

Supplementary Table 3.- Interaction sources overlap against the `RPGeNet` skeleton and wholegraph networks.

To provide an estimate of the overlap for the evidences gathered from each source, this table shows the intersection of total number of nodes and edges for the skeleton and wholegraph networks produced when the `RPGeNet` pipeline is run over each of the interactions sources separately. First row has the totals for the whole network that is produced when combining all the sources when running the full pipeline to create the graph levels integrated on the `RPGeNet v2` database, already shown on the previous Supplementary Tables. See also Supplementary Table 4 for further details about edges defined from the analysis of each separate interaction sources.

.

	Edges Summary				
	Total Directed	Mutual [A⇌B]	Assymetric [A⇀B]	Self-loop [A⇀A]	Total Non-Redundant
**BioGRID**					
Skeleton	23 480	6 243	10 994	0	17 237
WholeGraph	623 601	307 422	6 138	2 619	316 179
**STRING**					
Skeleton	20 791	6 422	7 947	0	14 369
WholeGraph	629 271	314 629	0	13	314 642
**PPaxe**					
Skeleton	3 654	1 554	546	0	2 100
WholeGraph	13 584	6 760	0	64	6 824
**RPGeNet v2 Core Network**					
Skeleton	35 528	9 601	16 326	0	25 927
WholeGraph	1 218 032	604 713	5 931	2 675	613 319

Supplementary Table 4.- Edges classification for the standalone pipeline analyses over each separate interaction sources versus RPGeNet core network.

The “Total Interactions” column shows the total number of edges stored in the database (without taking into account the number of evidences), counting reciprocal interactions twice (A⇀B is different from B⇀A). The “Non-redundant Interactions” column refers to the number of interactions in RPGeNet independently of the direction, and thus, the pair A⇀B and B⇀A is only counted once. Further details on all graph levels for the RPGeNet core network are provided on Supplementary Table 2.

Driver Genes	Predicted Interactions	Putative ~~Interactions~~Interactors
DTHD1 *	10	*AIPL1, CHD3, CNGB1, IFI27, MACC1, PID1, SH3BP4, UNC5A, UNC5C, UNC5CL*
IMPG2 *	10	*C2orf71, C8orf37, EYS, FAM161A, PRCD, TMEM252, TTC8, TULP1, ZNF408, ZNF513*
KCNJ13 *	10	*COL11A1, COL4A3, EDNRB, GJA5, IGSF11, KCNE3, KCNK5, KCNN4, KCNQ1, PNP*
MIR204	0	-
MT_TH	0	-
MT_TL1	0	-
MT_TP	0	-
PLA2G5	~~9~~10	*ALOX5, ENSG00000168970, PLA2G15, PLA2G16, PLA2G4D, PLA2G7, PLD1, ~~PLTG21, PLTGS2~~PLA2G4A, PTGS1, PTGS2*
PRCD *	10	*C2orf71, CERKL, CNGB1, FRMD6, IMPG2, PDE6A, PDE6B, RPE65, SLC4A3, ZNF513*
RAX2	10	*AKTIP, C8orf37, CRX, DPRX, FIZ1, GUCA1A, LEUTX, NRL, PITPNM3, PLAC9*
~~*RP1*~~	~~10~~	~~*GAK, IMPDH1, PLIN2, POLE, PRAME, PRPF31, RP2, SLC29A1, SP3, UBALD1*~~
RDH12	10	*RBP1, RBP2, RBP5, RETSAT, ALDH1A1, ALDH1A2, LRAT, AOX1, CYP26A1, BCO1*
SLC7A14 *	10	*ANKS4B, CAPN9, EIF5A2, GART, MFSD9, NMNAT2, RASD2, RPL22L1, SLC15A5, SLCO1B7*
SRD5A3	10	*SRD5A1, AKR1C1, AKR1C2, AKR1C3, AKR1D1, DOLK, HSD3B2, HSD17B3, HSD17B6, CYP17A1*
TRNT1	10	*ACOT13, ELAC1, ELAC2, FARS2, LARS2, PNPT1, TRIT1, TRMT10C, YARS, YARS2*
TTPA	10	*ATCAY, BNIP2, CYP4F2, EIF3K, FXN, PRUNE2, SLC6A11, SRR, RRS1, TRIM37*
C1QTNF5 (CTPR5)	4	*PATE4, TNFAIP8L1, ENSG00000235718, ENSG00000259159*

Supplementary Table ~~2~~5.- Predicted interactions for unconnected drivers genes on RPGeNet core network.

Driver genes can be unconnected to the core interactions whole-network graph because there are no known experimentally validated interactions that link them to the rest of nodes from the whole-network graph. Number of predicted interactions and putative interactors were retrieved from STRING database. The predicted interactions were not included into the whole core network on this release. Bottom row shows a driver gene, *C1QTNF5* (formerly identified as *CTPR5*), which is aggregated by the protocol to the growing graph at level 1 expansion yet it only has a validated interaction to itself from that point to the whole-graph. From seven unconnected driver genes of the RPGeNet previous version (2), five are still listed on this table—those marked with * —, while the updated interaction evidences made possible to connect the other two (*HGSNAT* and *PCARE*, the latter formerly identified as *C2ORF71*).

**Non-Driver Genes**	**Predicted Interactions**	**Putative ~~Interactions~~Interactors**
~~*CLC*~~	~~10~~	~~*ADH1A, CHD7, EPX, GALE, ISYNA1, LGALS3, LGALS12, PAICS, RNASE2, RNASE3*~~
DEFB106A	10	*DEFB103A, DEFB103B, DEFB104A, DEFB104B, DEFB105A, DEFB105B,* ***DEFB106B****, DEFB107A, DEFB107B, DEFB108B*
DEFB106B	10	*DEFB103A, DEFB103B, DEFB104A, DEFB104B, DEFB105A, DEFB105B,* ***DEFB106A****, DEFB107A, DEFB107B, DEFB108B*
DFNB32	0	-
DFNA37	0	-
~~*IGSF11*~~	~~10~~	~~*C1orf204, CCDC74A, CSF1R, ENSG00000278289, GPATCH2, KCNJ13, LPP , REV3L, SLC37A4, TSPAN3*~~
MFSD2A	10	*CDC5L, ERVFRD-1, KIAA1919, MFSD5, MFSD10, MFSD11, SLC25A33, SVOP, SVOPL, SYSP2L*
MFSD2B	10	*AP1S3, C9orf66, C16orf59,* ~~*C15orf70*~~*C16orf70, DDRGK1, EXOC3L4, MYLK3, RFX4, SAMD14, SUSD1*
~~*SCUBE1*~~	~~10~~	~~*ANTXR2, C1R, C1s, CARD14, CCDC129, GCC2, MYO9B, PTCH2, SMPDL3A, WDFY3*~~
PDSS1	10	*COQ2, COQ6, COQ9, DHDDS, FDPS, FDFT1, FNTA, FNTB, GGPS1,* ***PDSS2***
PDSS2	10	*COQ2, COQ6, COQ9, DHDDS, FDPS, FDFT1, FNTA, FNTB, GGPS1,* ***PDSS1***
SLC22A7	10	*ABCB11, ABCG2, ENSG00000257046, SLC10A1, SLC22A8, SLCO1A2, SLCO1B1, SLCO1B3, SLCO2B1, UCK2*
SLC22A10	10	*OR5AR1, OR10X1, SLC7A13, SLC28A1, SLC35G4P, SLCO1A2, SLCO1B7, SLCO3A1, SLCO4A1, SLCO4C1,*
ADGRL4 (ELTD1)	10	*CLEC14A, EMCN, GSTO1, KNG1, LGALS8, MYCT1, NAA10, NEUROD6, PHACTR1, ZNF366*
SLC24A2	10	*CNGA3, CRKL, GUCA1C, HHIP, KCNIP1, MAP3K4, RCVRN, SLC6A1, SH3GL2, TRIM4*
TMEM196	10	*BTBD17, CDCA7L, DNAH11, ITGB8, MACC1, RAPGEF5, SP4, TMEM63C, TMEM184C, TMEM207*

Supplementary Table ~~3~~6.- Predicted interactions for "unconnected" non-driver genes on RPGeNet core network.

Non-driver genes can be unconnected to the core interactions whole-network graph because there are no known experimentally validated interactions that link them ~~to the rest of nodes~~ from the whole-~~network~~ graph network level to the rest of nodes at any level of the RPGeNet core network. Number of predicted interactions and putative interactors were retrieved from STRING database. The predicted interactions were not included into the whole core network on this release. The last three genes have self-interactions, while the others are connected only among them in pairs at the whole-graph level (as for instance, *DEFB106A* and *DEFB106B*).

Supplementary File 1.- "SFile1_RPGeNet_Upgrade_2019_Database_Figure3.json" JavaScript JSON file containing the graph data for the nodes and edges found by the query described on Figure 3, as well as the coordinates to place the nodes with the same layout as shown on this figure. To reproduce such figure, one can upload this file by clicking on the "*Upload*" button of the controls panel in the Network Explorer window, or from the "*Network Explorer*" web form section at the RPGeNet home page. If no gene identifier is provided when clicking on the "*Explore Network*" button from that form page, a pop up panel will appear requesting to upload a graph file, such the one provided.

Supplementary File 2.- "SFile2_RPGeNet_Upgrade_2019_Database_Figure6A.json" JavaScript JSON file containing the graph data for the nodes and edges found by the query described on Figure 6A, as well as the coordinates to place the nodes with the same layout as shown on this figure. To reproduce such figure, one can upload this file by clicking on the "*Upload*" button of the controls panel in the Network Explorer window, or from the "*Network Explorer*" web form section at the RPGeNet home page. If no gene identifier is provided when clicking on the "*Explore Network*" button from that form page, a pop up panel will appear requesting to upload a graph file, such the one provided.

Supplementary File 3.- "SFile3_RPGeNet_Upgrade_2019_Database_Figure6B.json" JavaScript JSON file containing the graph data for the nodes and edges found by the query described on Figure 6B, as well as the coordinates to place the nodes with the same layout as shown on this figure. To reproduce such figure, one can upload this file by clicking on the "*Upload*" button of the controls panel in the Network Explorer window, or from the "*Network Explorer*" web form section at the RPGeNet home page. If no gene identifier is provided when clicking on the "*Explore Network*" button from that form page, a pop up panel will appear requesting to upload a graph file, such the one provided.
