# Supplementary material for: RPGeNet v2.0: expanding the universe of retinal disease gene interactions network
Source: Database (Oxford). 2019 Nov 11;2019:baz120. doi: 10.1093/database/baz120 (PMC6846243; doi:10.1093/database/baz120)
Supplement: Supplementary_data_baz120 [file supplementary_data_baz120.zip › SFigure1_2_3_RPGeNet_Upgrade_2019_Database_baz120.pdf]

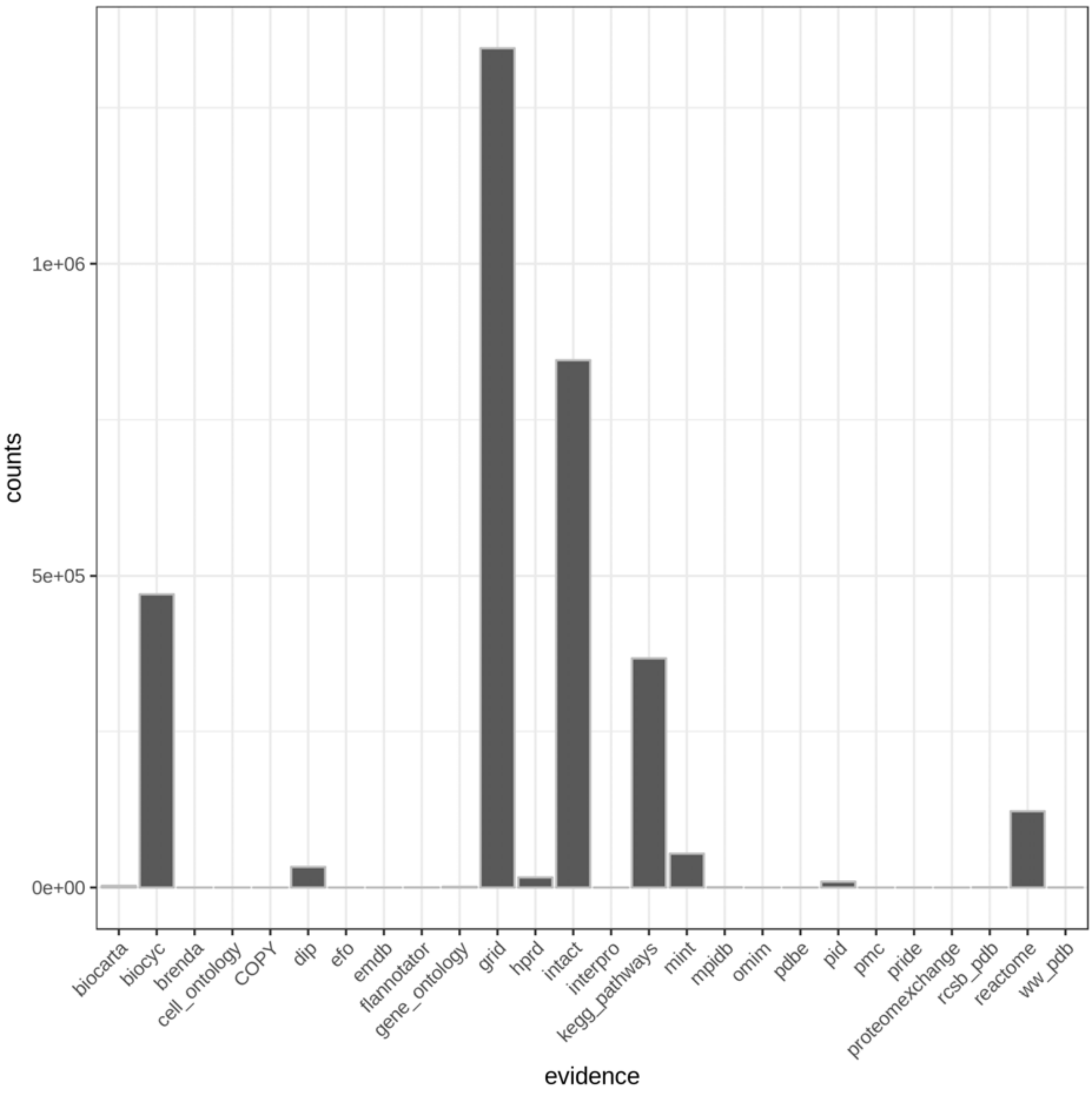

Supplementary Figure 1.- Bar plot with distribution of the different evidences used by `STRING`. `STRING` is a protein-to-protein interactions database that includes predictions and experimentally validated interactions. The experimentally validated interactions include evidences from different sources. The proportion of such evidences is shown in this bar-plot. `GRID`, `INTACT`, `KEGG`, `BIOCARTA` and `REACTOME` are the most common sources of experimentally-validated interactions from `STRING`.

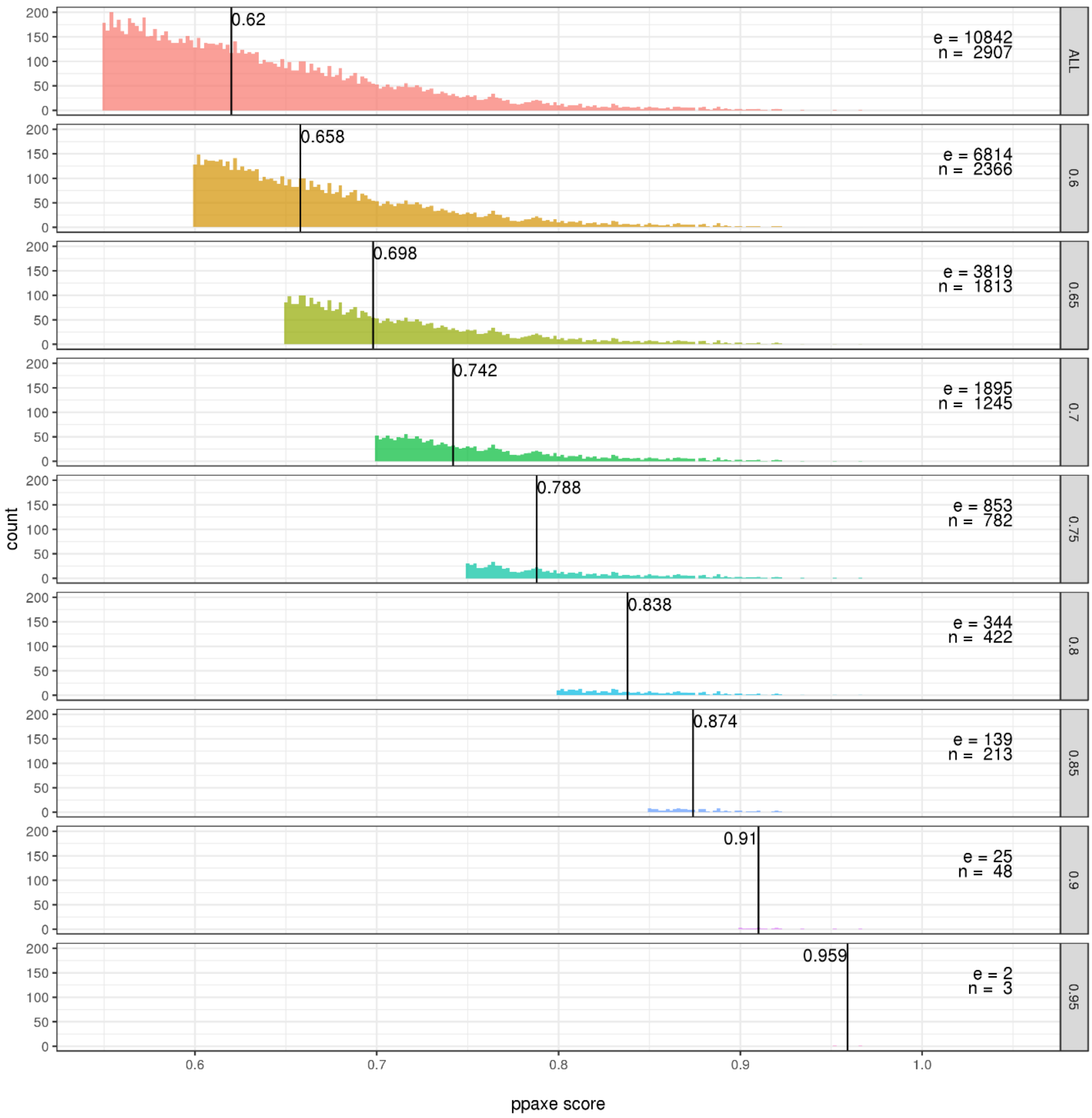

Supplementary Figure 2.- Choosing the optimal votes cut-off for `PPaxe`.

The graph shows the distribution of the un-normalized confidence score given to the interactions detected by PPaxe with respect to the number of PPaxe interactions (counts), and the average score depending on the cut-off score chosen (vertical line). In an attempt to optimize the number interactions while reducing false positive interactions and increasing false-negatives, the cut-off score chosen was 0.65, which was the value that yielded a minimum precision of 90% in the validation assessment performed in the original PPaxe manuscript (6). “e” and “n” correspond to the number of edges and nodes respectively that are passing the cut-off threshold. Panels categorize by the percentage of votes by the random-forest classifier.

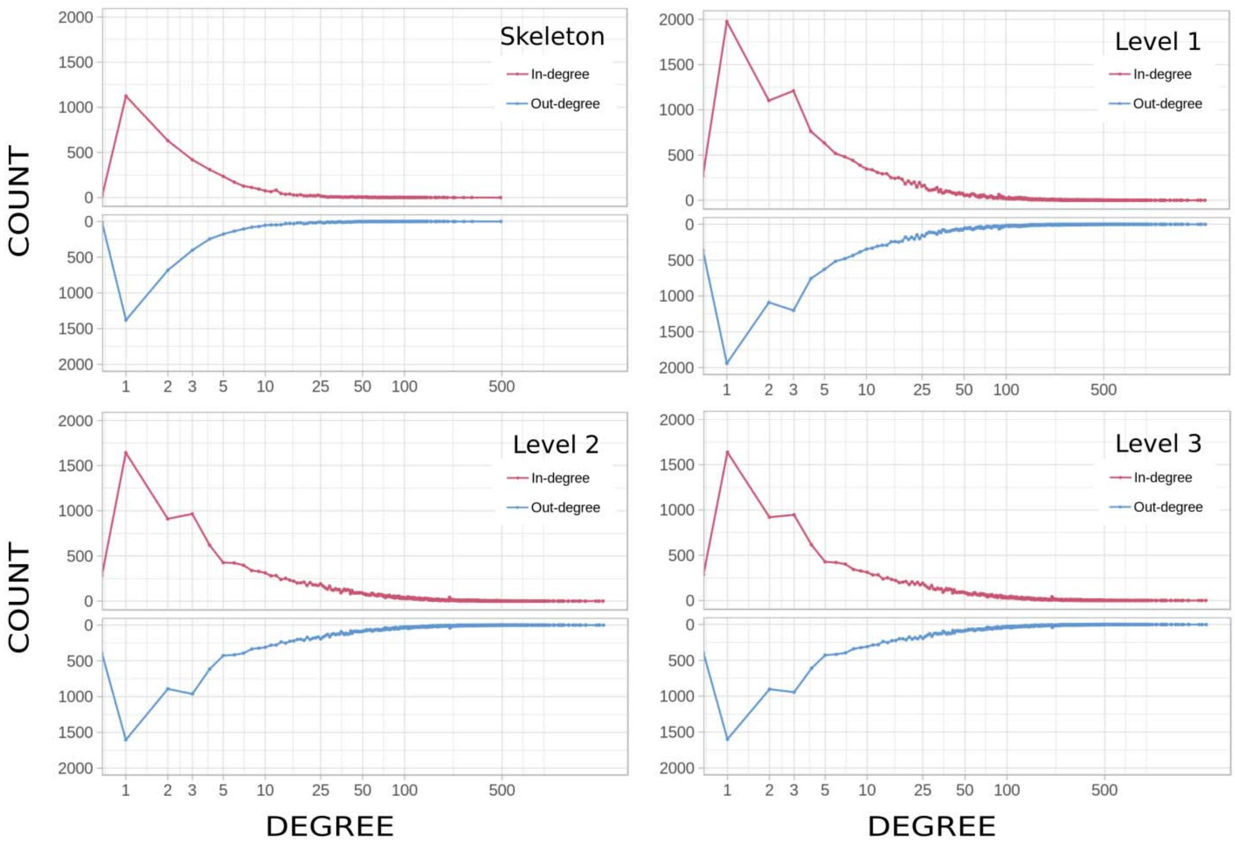

Supplementary Figure 3.- Comparing the number of counts between in and out degrees for each level of the RPGeNet core network.

In the skeleton graph the much smaller area between the in-/out-degree lines in comparison to higher level graphs denotes a smaller number of interactions. Comparing the skeleton with level one, there is a large increase in counts for lower degrees. However, as the graph is expanded to levels two and three, a decrease of counts in lower degrees can be observed. This can be explained by a larger jump in total number of interactions between skeleton and level one, followed by smaller increments in new interactions and a slower increase in degree from levels one to three, as new nodes and interactions are introduced.
