# Supplementary material for: RPGeNet v2.0: expanding the universe of retinal disease gene interactions network
Source: Database (Oxford). 2019 Nov 11;2019:baz120. doi: 10.1093/database/baz120 (PMC6846243; doi:10.1093/database/baz120)
Supplement: Supplementary_data_baz120 [file supplementary_data_baz120.zip › STable1_2_3_4_5_6_RPGeNet_Upgrade_2019_Database_baz120.pdf]

GRAPH STATS	RPGeNet v1		RPGeNet v2 — All Sets					BioGRID Only		STRING Only		PPaxe Only	
	Skeleton	WholeGrap	Skeleton	Level1	Level2	Level3	WholeGrap	Skeleton	WholeGrap	Skeleton	WholeGrap	Skeleton	WholeGrap
**Total #Nodes**	1 294	22 372	4 018	17 851	18 512	18 527	18 542	3 057	15 205	2 555	13 325	762	3 146
**Isolated Nodes**	7	7	16	15	15	15	15	71	70	56	56	93	84
**Adjacent Drivers**	103 of **110**		260 of **276**	261 of **276**				205 of **276**	206 of **276**	220 of **276**		183 of **276**	192 of **276**
**Total #Edges**	5 883	752 062	35 528	932 340	1217 902	1 218 017	1 218 032	23 480	623 601	20 791	629 271	3 654	13 584
**Mutual** [A⇌B]	1 082	319 928	9 601	462 988	604 652	604 707	604 713	6 243	307 422	6 422	314 629	1 554	6 760
**Assymetric** [A→B]	3 719	106 907	16 326	5 074	5 931	5 931	5 931	10 994	6 138	7 947	0	546	0
**Self-loop** [A→A]	0	5 299	0	1 290	2 667	2 672	2 675	0	2 619	0	13	0	64
**Total Non-Redundant**	4 801	432 134	25 927	469 352	613 250	613 310	613 319	17 237	316 179	14 369	314 642	2 100	6 824
**Graph Density**	0.0035	0.0015	0.0022	0.0029	0.0036	0.0035	0.0035	0.0025	0.0027	0.0032	0.0035	0.0063	0.0014
**Avg. Clustering**	0.0433	0.1445	0.0613	0.1449	0.2331	0.2331	0.2331	0.0448	0.0737	0.1508	0.5512	0.0959	0.0580
**Graph Diameter**	10	9	8	6	7	8	8	8	7	9	10	13	13
**Graph Reciprocity**	0.3678	0.8568	0.5405	0.9946	0.9951	0.9951	0.9951	0.5318	0.9901	0.6178	1.0000	0.8506	1.0000
**Avg. Degree**	9.0927	67.2324	17.6844	104.458	131.5797	131.4856	131.3809	15.3615	82.0258	16.2748	94.4497	9.5906	8.6357
**Avg. Closeness**	0.0969	0.0185	0.052	0.0529	0.0529	0.0529	0.0295	0.0128	0.0116	0.0165	0.0052	0.0093	0.0024
**Betweenness**	3	32 888.11	10	33	34	35	35 044.91	7	28 684.70	6	31 567.57	1 635.56	7 714.01
**Avg. Edge Betw.**	1	1 480.75	1 629.48	980.37	812.19	814.28	814.27	1	1 064.77	1	942.69	463.38	2 354.06
**Avg. Coreness**	4.7751	39.8983	9.1309	54.8258	77.0557	77.0063	76.9456	7.9326	42.0278	8.7366	67.3668	5.1273	4.6300
**Avg. Eccentricity**	6.7272	4.5465	5.6904	4.5666	4.9552	5.8732	5.8691	5.5741	5.1786	6.2693	6.6453	6.8307	7.9259
**Avg. Path Length**	4.1559	2.9473	3.6155	2.8810	2.8969	2.9000	2.9000	3.5936	2.9143	3.8110	3.4375	3.7888	4.1479

Supplementary Table 1.- Topology of the `RPGeNet` interactions graph.

This table shows the total number of nodes (genes/proteins, where adjacent nodes can be calculated by substracting *isolated* from *total nodes*) and edges (interactions, including counts by the type of relation between pairs of adjacent nodes: mutual-, asymmetric- and self-interactions), for every level of the `RPGeNet` database graph (see "`RPGeNet v2` All Sets" block). For comparison purposes, graph stats are provided for the previous `RPGeNet v1` version, as well as those for the interaction networks that can be produced separately from each of the evidence sources (blocks named as "`BioGRID` Only", "`STRING` Only", and "`PPaxe` Only", respectively). Most of the *isolated nodes* correspond to drivers without interaction evidences from the selected sources, and those numbers complement the values from the *adjacent drivers* row. Graph statistics were described in depth on Newman *et al* [1]. In br: *graph density* is the ratio between edges and the total number of possible vertices (nodes); *average clustering coefficient* (or the tendency of nodes to cluster together) is a measure of how complete the neighborhood of a node is, over all the nodes of the network; *diameter* is the maximum distance between two nodes; *reciprocity* is the; *average degree* is the average number of edges per node; *closeness* is the average length of shortest path between a node and every other node in the network; *betweenness* is a measure of the number of times a node is found within the shortest paths between two other nodes; *coreness* defines the shell index of the vertices of a network; *eccentricity* is the maximum of the shortest distances of a node with respect all other nodes in the graph; finally, *average path length* is a measure of the average distance between two nodes. Graph stats produced with `python-igraph` library (v0.7.0, see further details at https://igraph.org/python/).

1. Newman MEJ, Barabási A-L, Watts DJ. The structure and dynamics of networks. 2006. p. 624

		Skeleton	Level1	Level2	Level3	WholeGraph
GRAPH SUMMARY	**Total UNIQUE NODES**	4 018	17 851	18 512	18 527	18 542
	Adjacent Nodes	4 002	17 836	18 497	18 512	18 527
	Isolated Nodes	16	15	15	15	15
	**NODES by Source**					
	BioGRID	3 677	14 831	15 132	15 136	15 139
	STRING	3 580	12 864	13 253	13 263	13 269
	PPaxe	1 407	3 016	3 054	3 056	3 062
	TOTAL	8 664	30 711	31 439	31 455	31 470
	Nodes Source "redundancy"	*215.63%*	*172.04%*	*169.83%*	*169.78%*	*169.72%*
	**Total DIRECTED EDGES**	35 528	932 340	1 217 902	1 218 017	1 218 032
	Mutual [A⇄B]	9 601	462 988	604 652	604 707	604 713
	Assymetric [A⇀B]	16 326	5 074	5 931	5 931	5 931
	Self-loop [A⇀A]	0	1 290	2 667	2 672	2 675
	Total Non-Redundant	25 927	469 352	613 250	613 310	613 319
	Directed Edges "redundancy"	*137.03%*	*198.64%*	*198.60%*	*198.60%*	*198.60%*
	**EDGES by Source**					
	BioGRID all	22 914	518 478	623 643	623 656	623 659
	BioGRID only	*21 334*	*483 667*	*579 207*	*579 220*	*579 220*
	STRING all	12 563	440 111	629 167	629 265	629 271
	STRING only	*10 599*	*402 920*	*582 094*	*582 190*	*582 190*
	PPaxe all	2 277	12 282	13 572	13 578	13 584
	PPaxe only	*1 534*	*8 049*	*8 984*	*8 988*	*8 988*
	TOTAL	37 754	970 871	1 266 382	1 266 499	1 266 514
	Edges Source "redundancy"	*145.62%*	*206.85%*	*206.50%*	*206.50%*	*206.50%*
EVIDENCE SUMMARY	**TOTAL EVIDENCES**	75 447	2 371 305	3 209 677	3 209 856	3 209 871
	**By Class**					
	Genetic evidences	257	6 018	7 062	7 063	7 063
	Avg. evids x directed edge	*0.007*	*0.006*	*0.006*	*0.006*	*0.006*
	Physical evidences	70 561	2 342 154	3 177 567	3 177 739	3 177 748
	Avg. evids x directed edge	*1.986*	*2.512*	*2.609*	*2.609*	*2.609*
	Unknown evids (PPaxe)	4 629	23 133	25 048	25 054	25 060
	Avg. evids x directed edge	*0.130*	*0.025*	*0.021*	*0.021*	*0.021*
	**By Source**					
	BioGRID	31 726	705 485	842 798	842 815	842 818
	Physical interactions	*31 469*	*699 467*	*835 736*	*835 752*	*835 755*
	Genetic Interactions	*257*	*6 018*	*7 062*	*7 063*	*7 063*
	Avg. evids x directed edge	*0.893*	*0.757*	*0.692*	*0.692*	*0.692*
	STRING	39 092	1 642 687	2 341 831	2 341 987	2 341 993
	Avg. evids x directed edge	*1.100*	*1.762*	*1.923*	*1.923*	*1.923*
	PPaxe	4 629	23 133	25 048	25 054	25 060
	Avg. evids x directed edge	*0.130*	*0.025*	*0.021*	*0.021*	*0.021*
EDGES with STRING SCORE	**With any STRING score**	**12 563**	**440 111**	**629 167**	**629 265**	**629 271**
	With "experimental" score	2 696	75 075	109 191	109 231	109 235
	With "database" score	9 112	371 666	541 032	541 082	541 084
	With "text-mining" score	8 803	248 133	345 610	345 682	345 686
	With "co-expression" score	2 227	107 246	157 974	158 008	158 010
	With "neighborhood" score	0	0	0	0	0
	With gene-"fusion" score	16	1 136	2 384	2 384	2 384
	With "co-occurrence" score	124	6 320	8 749	8 755	8 757

Supplementary Table 2.- Source origin and redundancy of evidences for RPGeNet core network interactions.

First block accounts for the number of nodes and edges supported by each input source at each graph level of the core network. Second block provides information about distinct evidences used to weight the interactions. Number of interactions on the core network with STRING scores is provided on the last block to complement evidences (also shown in the interaction information pop-up cards like the one shown in Figure 5 right panel). RPGeNet models interactions as directed edges between nodes, and thus, represents undirected interactions between genes as two separate interactions. The "Total Interactions" column shows the total number of edges stored in the database (without taking into account the number of evidences), counting reciprocal interactions twice (A⇀B is different from B⇀A). The "Non-redundant Interactions" column refers to the number of interactions in RPGeNet independently of the direction, and thus, the pair A⇀B and B⇀A is only counted once. The Non-redundant interactions count criteria is equivalent to the one used by the BioGRID database (https://wiki.thebiogrid.org/doku.php/build_3.5.171).

	Skeleton Total # Nodes	Skeleton Total # Edges	WholeGraph Total # Nodes	WholeGraph Total # Edges
**Run Together (`RPGeNet v2`)**	4 018	35 528	18 542	1 218 032
**Overlapping Sets**	4 767	46 610	18 539	1 217 974
`BioGRID only`	2 031	22 757	4 956	579 165
`STRING only`	1 389	19 650	2 598	582 196
`PPaxe only`	136	2 994	113	8 994
`BioGRID ∩ STRING`	585	549	7 839	43 029
`BioGRID ∩ PPaxe`	45	68	145	544
`STRING ∩ PPaxe`	185	486	623	3 183
`BioGRID ∩ STRING ∩ PPaxe`	396	106	2 265	863
**Total from `BioGRID`**	3 057	23 480	15 205	623 601
**Total from `STRING`**	2 555	20 791	13 325	629 271
**Total from `PPaxe`**	762	3 654	3 146	13 584

Supplementary Table 3.- Interaction sources overlap against the `RPGeNet` skeleton and wholegraph networks.

To provide an estimate of the overlap for the evidences gathered from each source, this table shows the intersection of total number of nodes and edges for the skeleton and wholegraph networks produced when the `RPGeNet` pipeline is run over each of the interactions sources separately. First row has the totals for the whole network that is produced when combining all the sources when running the full pipeline to create the graph levels integrated on the `RPGeNet v2` database, already shown on the previous Supplementary Tables. See also Supplementary Table 4 for further details about edges defined from the analysis of each separate interaction sources.

	Edges Summary				
	Total Directed	Mutual [A⇌B]	Assymetric [A⇀B]	Self-loop [A⇀A]	Total Non-Redundant
**BioGRID**					
Skeleton	23 480	6 243	10 994	0	17 237
WholeGraph	623 601	307 422	6 138	2 619	316 179
**STRING**					
Skeleton	20 791	6 422	7 947	0	14 369
WholeGraph	629 271	314 629	0	13	314 642
**PPaxe**					
Skeleton	3 654	1 554	546	0	2 100
WholeGraph	13 584	6 760	0	64	6 824
**RPGeNet v2 Core Network**					
Skeleton	35 528	9 601	16 326	0	25 927
WholeGraph	1 218 032	604 713	5 931	2 675	613 319

Supplementary Table 4.- Edges classification for the standalone pipeline analyses over each separate interaction sources versus RPGeNet core network.

The “Total Interactions” column shows the total number of edges stored in the database (without taking into account the number of evidences), counting reciprocal interactions twice (A⇀B is different from B⇀A). The “Non-redundant Interactions” column refers to the number of interactions in RPGeNet independently of the direction, and thus, the pair A⇀B and B⇀A is only counted once. Further details on all graph levels for the RPGeNet core network are provided on Supplementary Table 2.

**Driver Genes**	**Predicted Interactions**	**Putative Interactors**
DTHD1 *	10	*AIPL1, CHD3, CNGB1, IFI27, MACC1, PID1, SH3BP4, UNC5A, UNC5C, UNC5CL*
IMPG2 *	10	*C2orf71, C8orf37, EYS, FAM161A, PRCD, TMEM252, TTC8, TULP1, ZNF408, ZNF513*
KCNJ13 *	10	*COL11A1, COL4A3, EDNRB, GJA5, IGSF11, KCNE3, KCNK5, KCNN4, KCNQ1, PNP*
MIR204	0	-
MT-TH	0	-
MT-TL1	0	-
MT-TP	0	-
PLA2G5	10	*ALOX5, ENSG00000168970, PLA2G15, PLA2G16, PLA2G4D, PLA2G7, PLD1, PLA2G4A, PTGS1, PTGS2*
PRCD *	10	*C2orf71, CERKL, CNGB1, FRMD6, IMPG2, PDE6A, PDE6B, RPE65, SLC4A3, ZNF513*
RAX2	10	*AKTIP, C8orf37, CRX, DPRX, FIZ1, GUCA1A, LEUTX, NRL, PITPNM3, PLAC9*
RDH12	10	*RBP1, RBP2, RBP5, RETSAT, ALDH1A1, ALDH1A2, LRAT, AOX1, CYP26A1, BCO1*
SLC7A14 *	10	*ANKS4B, CAPN9, EIF5A2, GART, MFSD9, NMNAT2, RASD2, RPL22L1, SLC15A5, SLCO1B7*
SRD5A3	10	*SRD5A1, AKR1C1, AKR1C2, AKR1C3, AKR1D1, DOLK, HSD3B2, HSD17B3, HSD17B6, CYP17A1*
TRNT1	10	*ACOT13, ELAC1, ELAC2, FARS2, LARS2, PNPT1, TRIT1, TRMT10C, YARS, YARS2*
TTPA	10	*ATCAY, BNIP2, CYP4F2, EIF3K, FXN, PRUNE2, SLC6A11, SRR, RRS1, TRIM37*
C1QTNF5 (*CTPR5*)	4	*PATE4, TNFAIP8L1, ENSG00000235718, ENSG00000259159*

Supplementary Table 5.- Predicted interactions for unconnected drivers genes on RPGeNet core network.

Driver genes can be unconnected to the core interactions whole-network graph because there are no known experimentally validated interactions that link them to the rest of nodes from the whole-network graph. Number of predicted interactions and putative interactors were retrieved from STRING database. The predicted interactions were not included into the whole core network on this release. Bottom row shows a driver gene, *C1QTNF5* (formerly identified as *CTPR5*), which is aggregated by the protocol to the growing graph at level 1 expansion yet it only has a validated interaction to itself from that point to the whole-graph. From seven unconnected driver genes of the RPGeNet previous version (2), five are still listed on this table—those marked with * —, while the updated interaction evidences made possible to connect the other two (*HGSNAT* and *PCARE*, the latter formerly identified as *C2ORF71*).

**Non-Driver Genes**	**Predicted Interactions**	**Putative Interactors**
DEFB106A	10	*DEFB103A, DEFB103B, DEFB104A, DEFB104B, DEFB105A, DEFB105B,* ***DEFB106B****, DEFB107A, DEFB107B, DEFB108B*
DEFB106B	10	*DEFB103A, DEFB103B, DEFB104A, DEFB104B, DEFB105A, DEFB105B,* ***DEFB106A****, DEFB107A, DEFB107B, DEFB108B*
DFNB32	0	-
DFNA37	0	-
MFSD2A	10	*CDC5L, ERVFRD-1, KIAA1919, MFSD5, MFSD10, MFSD11, SLC25A33, SVOP, SVOPL, SYSP2L*
MFSD2B	10	*AP1S3, C9orf66, C16orf59, C16orf70, DDRGK1, EXOC3L4, MYLK3, RFX4, SAMD14, SUSD1*
PDSS1	10	*COQ2, COQ6, COQ9, DHDDS, FDPS, FDFT1, FNTA, FNTB, GGPS1,* ***PDSS2***
PDSS2	10	*COQ2, COQ6, COQ9, DHDDS, FDPS, FDFT1, FNTA, FNTB, GGPS1,* ***PDSS1***
SLC22A7	10	*ABCB11, ABCG2, ENSG00000257046, SLC10A1, SLC22A8, SLCO1A2, SLCO1B1, SLCO1B3, SLCO2B1, UCK2*
SLC22A10	10	*OR5AR1, OR10X1, SLC7A13, SLC28A1, SLC35G4P, SLCO1A2, SLCO1B7, SLCO3A1, SLCO4A1, SLCO4C,*
ADGRL4 (*ELTD1*)	10	*CLEC14A, EMCN, GSTO1, KNG1, LGALS8, MYCT1, NAA10, NEUROD6, PHACTR1, ZNF366*
SLC24A2	10	*CNGA3, CRKL, GUCA1C, HHIP, KCNIP1, MAP3K4, RCVRN, SLC6A1, SH3GL2, TRIM4*
TMEM196	10	*BTBD17, CDCA7L, DNAH11, ITGB8, MACC1, RAPGEF5, SP4, TMEM63C, TMEM184C, TMEM207*

Supplementary Table 6.- Predicted interactions for "unconnected" non-driver genes on `RPGeNet` core network.

Non-driver genes can be unconnected to the core interactions whole-network graph because there are no known experimentally validated interactions that link them from the whole-graph network level to the rest of nodes at any level of the `RPGeNet` core network. Number of predicted interactions and putative interactors were retrieved from `STRING` database. The predicted interactions were not included into the whole core network on this release. The last three genes have self-interactions, while the others are connected only among them in pairs at the whole-graph level (as for instance, *DEFB106A* and *DEFB106B*).
